# Discovery of a New CDK4/6 and PI3K/AKT Multiple Kinase Inhibitor Aminoquinol for the Treatment of Hepatocellular Carcinoma

**DOI:** 10.3389/fphar.2021.691769

**Published:** 2021-07-15

**Authors:** Zhong-Kun Xia, Wei Wang, Jian-Ge Qiu, Xi-Nan Shi, Hong-Jian Li, Rong Chen, Kun-Bin Ke, Chao Dong, Ying Zhu, Shi-Guo Wu, Rong-Ping Zhang, Zhuo-Ran Meng, Hui Zhao, Peng Gu, Kwong-Sak Leung, Man-Hon Wong, Xiao-Dong Liu, Feng-Mei Zhou, Jian-Ying Zhang, Ya-Ting Yao, Si-Jia Wang, Chun-Yang Zhang, Yan-Ru Qin, Marie Chia-mi Lin, Bing-Hua Jiang

**Affiliations:** ^1^ School of Basic Medical Sciences, Academy of Medical Science, Zhengzhou University, Zhengzhou, China; ^2^ Department of Pathology, Yunnan University of Chinese Medicine, Kunming, China; ^3^ XingYi People’ Hospital, Xingyi, China; ^4^ CUHK-SDU Joint Laboratory on Reproductive Genetics, School of Biomedical Sciences, Chinese University of Hong Kong, Hong Kong, China; ^5^ Department of Physiology, Yunnan University of Chinese Medicine, Kunming, China; ^6^ Department of Urology, The First Affiliated Hospital of Kunming Medical University, Kunming, China; ^7^ Department of the Second Medical Oncology, The Third Affiliated Hospital of Kunming Medical University, Yunnan Tumor Hospital, Kunming, China; ^8^ Department of Cadre Medical Branch, The Third Affiliated Hospital of Kunming Medical University, Kunming, China; ^9^ Department of Teaching and Research Section of Formulas of Chinese Medicine, Yunnan University of Chinese Medicine, Kunming, China; ^10^ School of Chinese Materia Medica and Yunnan Key Laboratory of Southern Medicinal Utilization, Yunnan University of Chinese Medicine,Kunming, China; ^11^ Department of Computer Science and Engineering, Chinese University of Hong Kong, Hong Kong, China; ^12^ College of Chemistry, Chemical Engineering and Materials Science, Shandong Normal University, Jinan, China; ^13^ Department of Oncology, The First Affiliated Hospital of Zhengzhou University, Zhengzhou, China; ^14^ Department of Pathology, Anatomy and Cell Biology, Thomas Jefferson University, Philadelphia, PA, United States

**Keywords:** aminoquinol, hepatocellular carcinoma, CDK4/6, RB, PI3K

## Abstract

**Background:** Hepatocellular carcinoma (HCC) is a lethal malignancy lacking effective treatment. The Cyclin-dependent kinases 4/6 (CDK4/6) and PI3K/AKT signal pathways play pivotal roles in carcinogenesis and are promising therapeutic targets for HCC. Here we identified a new CDK4/6 and PI3K/AKT multi-kinase inhibitor for the treatment of HCC.

**Methods:** Using a repurposing and ensemble docking methodology, we screened a library of worldwide approved drugs to identify candidate CDK4/6 inhibitors. By MTT, apoptosis, and flow cytometry analysis, we investigated the effects of candidate drug in reducing cell-viability,inducing apoptosis, and causing cell-cycle arrest. The drug combination and thermal proteomic profiling (TPP) method were used to investigate whether the candidate drug produced antagonistic effect. The *in vivo* anti-cancer effect was performed in BALB/C nude mice subcutaneously xenografted with Huh7 cells.

**Results:** We demonstrated for the first time that the anti-plasmodium drug aminoquinol is a new CDK4/6 and PI3K/AKT inhibitor. Aminoquinol significantly decreased cell viability, induced apoptosis, increased the percentage of cells in G1 phase. Drug combination screening indicated that aminoquinol could produce antagonistic effect with the PI3K inhibitor LY294002. TPP analysis confirmed that aminoquinol significantly stabilized CDK4, CDK6, PI3K and AKT proteins. Finally, *in vivo* study in Huh7 cells xenografted nude mice demonstrated that aminoquinol exhibited strong anti-tumor activity, comparable to that of the leading cancer drug 5-fluorouracil with the combination treatment showed the highest therapeutic effect.

**Conclusion:** The present study indicates for the first time the discovery of a new CDK4/6 and PI3K/AKT multi-kinase inhibitor aminoquinol. It could be used alone or as a combination therapeutic strategy for the treatment of HCC.

## Introduction

Hepatocellular carcinoma (HCC) is the most common type of liver cancer, with only 30–40% of the patients eligible for curative treatments at the time of disease diagnosis (Liu et al., 2015). Surgical resection is the first line treatment, followed by liver transplantation and percutaneous ablation. However, there is a high frequency of tumor recurrence (Yang et al., 2015).

Targeted therapy for advanced HCC is a promising strategy, and HCC displays multiple drug targets. The multi-kinase inhibitor sorafenib has been used as the first-line agent in clinical practice since 2007. Recently, another multiple kinase inhibitor lenvatinib was approved to be the first line treatment. In addition, regorafenib and cabozantinib were recommended as second-line treatment for patients with advanced HCC who are resistant to sorafenib (Kudo 2020). However, most HCCs are resistant to currently available conventional chemotherapy and radiotherapy. Therefore, the development of new and more effective therapies against HCC is urgently needed.

Cyclin-dependent kinases (CDK) belongs to the family of serine/threonine protein kinase, which play pivotal roles in cell cycle regulation (Malumbres 2014), transcription, metabolism, neuro-physiological processes, cell differentiation and development (Asghar et al., 2015). In tumors cells, elevated CDK activities directly or indirectly increase cell proliferation, genomic instability (DNA mutations and chromosome deletion, etc.) and chromosome instability (chromosome number changes), that are critical for the development and progression of cancer (Malumbres and Barbacid 2009). Among them, CDK4/6 activities are necessary for the regulation of cell cycle in G1 phase. It has been well documented that the expression levels of CDK4/6 are significantly higher in many tumors (Yamamoto et al., 1995; Kim et al., 2000; Cohen 2002; Li et al., 2002). In HCC, 66.7% of patients were reported to have elevated CDK4 (Kim et al., 2000) and 46% have elevated CDK6 (Che et al., 2012).

PI3K/AKT signal pathway is one of the major intracellular signaling pathways which regulates diverse cellular functions, including cell proliferation, differentiation, translation regulation of protein synthesis, cell migration, and angiogenesis. PI3K-AKT-mTOR usually promotes survival by inhibiting pro-apoptotic factors and activating anti-apoptotic factors (Miricescu et al., 2020). The expressions of PI3K and AKT are elevated in HCC, and are significantly associated with reduced overall survival. Therefore, drugs targeting both CDK4/6 and PI3K/AKT signal pathways should be an excellent therapeutic strategy for HCC.

In this study, we applied a mixed computational and experimental strategy to repurpose approved small molecule drugs to identify inhibitor of CDK4/6 for HCC (Berman et al., 2000; Irwin and Shoichet 2005; Irwin et al., 2012; Li et al., 2012; Li et al., 2014; Huang and Wong 2016). We discovered aminoquinol as a new CD4/6 inhibitor with high anti-cancer activities in human liver cancer HepG2 and Huh7 cells. In addition, it also exhibited significant activity in Hep3B cells which lacks functional Rb gene, suggesting that it has other anti-cancer mechanisms in addition to CDK4/6. Through drug combination screening, we found that aminoquinol could produce weak synergistic effects with 5-Fu and the multi-kinase inhibitor soranfenib, however, clearly displayed antagonistic effects with the PI3K inhibitor LY294002. These results strongly suggested that it may also inhibited PI3K activity. Using Western blotting analysis, we demonstrated that aminoquinol treatment significantly decreased the expressions of key proteins in the CDK4/6 as well as the PI3K/AKT pathways. Thermal proteome profiling (TPP) (Jafari et al., 2014) analysis further suggested that proteins in both CDK4/6 and PI3K/AKT pathways are the binding targets of aminoquinol. Finally, in BALB/C nude mice subcutaneously xenografted with Huh7 cells, we demonstrated the *in vivo* anti-cancer effects by aminoquinol. Taken together, the present study indicates for the first time that aminoquinol is a new CDK4/6 and PI3K/AKT multi-kinase inhibitor and a potential candidate drug for the treatment of human HCC.

## Materials and Methods

### Ethics Statement

This study was approved by the laboratory animal ethics committees of Zhengzhou University.

### Chemicals and Anti-Bodies

Lifibrate, Nizofenone, Pimozide, Trifluperidol, Tosufloxacin, Cloricromene, Sulconazole, Sertindole, Aminoquinol, 5-Fuwere purchased from Sigma-Aldrich. Anti-cyclinD, anti-CDK2/4/6, anti-Rb, phosphorylated (pho)-CDK4/6, pho-Rb, AKT, pho-AKT and GAPDH were obtained from Cell Signaling Technology, Inc (Danvers, MA, United States). PI3K, mTOR, pho-mTOR were obtained from ZEN BIO.

### Cell Lines and Cell Culture Experimental Conditions

The human liver cancer Huh7, HepG2 and Hep3B cell lines were obtained from the American Type Culture Collection (Manassas, VA, United States). These cell lines were cultured in DMEM medium (GE Healthcare Life Sciences, Shanghai, China) containing 10% fetal bovine serum (FBS) (Invitrogen Life Technologies, Carlsbad, CA, United States) at 37°C in 5% CO2 and 95% humidified air. Cells were plated in 96-well, 24-well, or 6-well plates (Corning Incorporated, Corning, NY, United States) with medium containing 0.125% FBS for 24 h and then treated with medium containing 10% FBS and the test compounds at various concentrations and incubated for various times as indicated.

### MTT and CCK-8 Assays

For the MTT assay (Sigma-Aldrich), cells were plated at an initial density of 9×10^3^ cells/well in 96-well plates and incubated with 0.5 mg/ml MTT (Sigma-Aldrich) for 4 h. The medium was then discarded and 200 μl dimethylsulfoxide (Sigma-Aldrich) was added to dissolve the formed formazan crystals. The absorbance was measured at 570 nm with a Synergy 2 microplate reader (Bio-Tek Instruments, Inc., Winooski, VT, United States) according the standard protocol. CCK-8 assay was performed as described in the CCK-8 Kit (Dojindo Laboratories). Cells were seeded in 96-well plate, treated with various drugs for indicated time prior to the addition of CCK-8 solution and OD values were measured at 450 nm using a microplate reader.

### Cell Cycle Analysis

Cells (4×10^4^) were seeded in 24-well plates in DMEM medium containing 0.125% FBS for 24 h, and then treated with DMEM medium containing 10% FBS and various dose of aminoquinol for indicated time at 37°C. At the end of experiments, cells were fixed in ice-cold 70% ethanol and stained using a Coulter DNA-Prep Reagents kit (Beckman Coulter, Brea, CA, United States). Cellular DNA content of 1×10^4^ cells from each sample was determined using an EPICS xL4 flow cytometer (Beckman Coulter). The cell cycle phase distribution was analyzed using ModFit LT 2.0 software (Verity Software House, Topsham, ME, United States). All data were obtained from two separate experiments of which each was performed in triplicate.

### Cell Apoptosis Analysis

Cells were plated at 24-well plates with DMEM containing 0.125% FBS for 24 h and then treated with 10% FBS medium containing aminoquinol at concentrations 5 and 10 μM for 48 h. To quantify the apoptosis, the occurrence of apoptotic cells was determined by staining cells with both annexinV and propidium iodide (PI) Following manufacturer’s instructions (Life Technologies). Briefly, cells were trypsinized with 0.25% trypsin in the absence of ethylenediaminetetraacetic acid (EDTA), washed with PBS twice, and resuspended in 500 μl of binding buffer at a concentration of 2 × 10^5^–10^6^ cells/ml. Two microliters of annexin V-EGFP and 5 μl of PI were added to the suspension followed by 5–15 min of incubation in the dark, and the cells were analyzed using flow cytometry (CyFlow Space/Partec, Germany).

### Western Blot Analysis

Cells were plated on 6-well plates in DMEM medium containing 0.125% FBS for 24 h, and then treated with medium containing 10% FBS medium and various concentrations of aminoquinol for 6 h. Cells were harvested and the protein concentration was measured using a bicinchoninic acid Protein Assay kit (Thermo Fisher Scientific, Waltham, MA, United States). Equal amounts (10 μg protein) of cell lysates were resolved using 10% SDS-PAGE and transferred onto nitrocellulose membranes (Sigma-Aldrich). After blocking with 5% skim milk, blots were treated with the primary antibody at 4°C overnight, then with horseradish peroxidase-labeled secondary antibody. The proteins were measured using enhanced chemiluminescence detection system (Thermo Fisher scientific, United States). The primary antibodies were purchased commercially: anti-cyclin D1 (CST, no.2978), anti-CDK2 (CST, no.2546), anti-CDK4 (CST, no.12790), anti-phospho-CDK4 (CST, no.5884), anti-CDK6 (Abcam, no.13331), anti-phospho-CDK6 (ZEN BIO, no.530326), anti-Rb (CST, no.9313), anti-phospho-Rb (CST, no.9301), anti-KRAS (Proteintech, no.12063-1-AP), anti-NRAS (Proteintech, no.10724-AP), anti-c-Raf (CST, no.9422), anti-MEK1/2 (ZEN BIO, no.200424), anti-ERK1/2 (ZEN BIO, no.340750), anti-PI3K (ZEN BIO, no.380849), anti-AKT (CST, no.4685S), anti-phospho-AKT (CST, no.4060S), anti-mTOR (ZEN BIO, no.385034), anti-phospho-mTOR (ZEN BIO, no.385033), anti-GAPDH (CST, no.5174). All of the antibodies were diluted by 1:1,000 dilution.

### Quantitate the Synergy of Drug Combination

We quantitated the synergy of drug combination according to the Chou-Talalay method (Chou, 2011). Huh7 cells (5 × 10^3^cells/well) were plated in 96-well plates, cells were treated with indicated concentrations of aminoquinol and 5-Fu (Wang et al., 2018), sorafenib (Bollard et al., 2017), or LY294002 (Teo et al., 2017) for 72 h. Cell viability was determined by CCK-8 assay and the absorbance values measured at 450 nm using microplate reader. The combined effect was analyzed by CompuSyn software (www.combosyn. com), and the multiple drug dose-effect were calculated using the Median Effects methods described by Chou and Talalay to determine the fraction affected (Fa), and the combination index (CI). The fraction affected Fa = 1- (average OD value of experimental group/average OD value of control group), which represents the inhibition rate. The combination index CI = (D)1/(Dx)1+(D)2/(Dx)2, Dosage as a single drug (D), Dosage used in the combination drug (Dx). The definitions of CI are the following: 1) CI = 1 represents additive effect, 2) CI < 1 represents synergistic effect, and 3) CI > 1 represents antagonism (Chou, 2006; Chou, 2010; Chou, 2011).

### Thermal Proteome Profiling Analysis

TPP analysis were conducted as described previously (Jafari et al., 2014) (Franken et al., 2015). Huh7 cells were plated on 6-well plates in DMEM medium containing 10% FBS medium. After washing with PBS, cells were scraped off, suspended in the lysis buffer, then lysed with three freeze thaw cycles using liquid nitrogen. The lysate was centrifuged at 10800×*g* for 20 min in 4°C, the supernatant collected, and adjusted to 1 mg ml^−1^ (by BCA assay). The lysates were treated with 100 μM aminoquinol or DMSO at 25°C for 20 min with gentle mixing, and divided into 10 fractions for thermal profiling. Fractions were heated at the indicated temperatures (35–75°C) for 3 min, incubated for 3 min at room temperature, centrifuged at 15,000×*g* for 5 min at room temperature, and the supernatants were analyzed by Western blots.

### Evaluation of the Anti-cancer Activity of Aminoquinol *in vivo* in Nude Mice Xenografted With Huh7 Cells

Female BALB/C nude mice (n = 20, 4–5 weeks old; weighing 15 g; Vital River Laboratory Technology Co. Ltd., Beijing, China), were housed under pathogen-free conditions with a 12 h light/dark cycle, in an environment containing 50–80% humidity at 15–27°C, and cared for in accordance with the guidelines of the laboratory animal ethics committee of Zhengzhou University (Zhengzhou, China). The cages, food, and water of the mice were sterilized. To establish the xenograft model, 1 × 10^6^ Huh7 cells in 0.2 ml phosphate-buffered saline were injected subcuta-neously into the right flank of the mice (n = 5) and the tumor size was measured every day using a caliper. One week after inoculation, when the tumors grew to a volume of 80–100 mm^3^, mice were randomly divided into four groups (5 mice/group) and intraperitioneal injected with PBS (control), aminoquinol (35 mg/kg), 5-Fu (10 mg/kg), or aminoquinol (35 mg/kg) + 5-Fu (10 mg/kg), daily for 21 days. Tumor volume was calculated using formula V = ab^2^/2 (a = longest axis; b = shortest axis). At 21 days, mice were sacrificed by cervical dislocation, tumors excised and weighed, their images captured, and the tissues fixed for immunohistochemistry studies.

### Immunohistochemistry

Tumor tissues were fixed in 10% formalin, embedded in paraffin, sliced into 4 μm sections, deparaffinized, dehydrated, antigen retrieved, blocked with 5% goat serum, and incubated with the primary antibodies: anti-RB1 (CST, no.9313, 1: 500), anti-CDK4 (CST, no.12790, 1: 500), anti-CDK6 (CST, no.13331, 1: 500), anti-PI3K (ZEN BIO, no.220742, 1: 500), anti-AKT (CST, no.4685S, 1: 500), anti-phospho-AKT (CST, no.4060S, 1: 500), anti-phospho-mTOR (ZENBIO, no.380411, 1: 500) and anti-ki67 (SAB, no.48871, 1: 500). The slides were washed and incubated with biotinylated anti-mouse or anti-rabbit secondary antibodies. The peroxidase reaction was visualized using 3, 3′-diaminobenzidine tetrahydrochloride (DAB) and counterstained with hematoxylin. To quantitate the staining intensity, five random fields were chosen, and the numbers of total cells and positive cells were counted in each section under a microscope at 400× magnification. The percentage of positive cell populations from the five random fields was analyzed for statistical significance.

### Statistical Analysis

Data were obtained from at least three experiments. Values are expressed as the mean ± standard deviation. Statistical analysis was performed by Student’s t-test, and the results were analyzed using SPSS 16.0. *p* < 0.05 was considered to indicate a statistically significant difference between values.

## Results

### Discovery of Aminoquinol as a CDK4/6 Inhibitor and Determined Its Activity in Reducing the Viability of HepG2 and Huh7 Cells

To identify candidate CDK4/6 inhibitors, we used structure-based virtual screening method to screen for CDK4/6 inhibitor as described previously (Shi et al., 2018). Briefly, five X-ray crystallographic structures of CDK4 and eight X-ray crystallographic structures of CDK6 in complex with ligands were collected from the Protein Data Bank (PDB) (Berman et al., 2000; Huang and Wong 2016). A total of 3,167 drugs that have been approved by worldwide authorities were selected to constitute a library of compounds to screen, and their structures were collected from the ZINC database (Irwin and Shoichet 2005) (Irwin et al., 2012). These drugs were individually docked to the ATP binding pocket of CDK4/6, and sorted in the ascending order of their predicted binding free energy. The free and open-source docking software idock v2.2.1 (Li et al., 2014) developed by our group was applied to dock all of the compounds onto all of the CDK4/6 structures to predict their binding conformations as well as their binding affinities. The compounds were sorted in an ascending order according to their average predicted binding free energy, and the high-scoring compounds were manually examined based on in silico estimations of binding strength, appropriate molecular weight and other drug-like properties, complementary matching of molecular shape, plus some sense of intuition from a computational chemist’s experience. Finally, high-scoring compounds were shortlisted and 10 commercially available compounds (Supplementary Table 1) (Bueno 1965; Janssen et al., 1968; Ross 1970; Arnold et al., 1979; Dejana et al., 1982; Lassus et al., 1983; Fujimaki et al., 1988; Matsumoto et al., 1994; Muscatello et al., 2014) were purchased for subsequent wet-lab validations.

**FIGURE 1:**
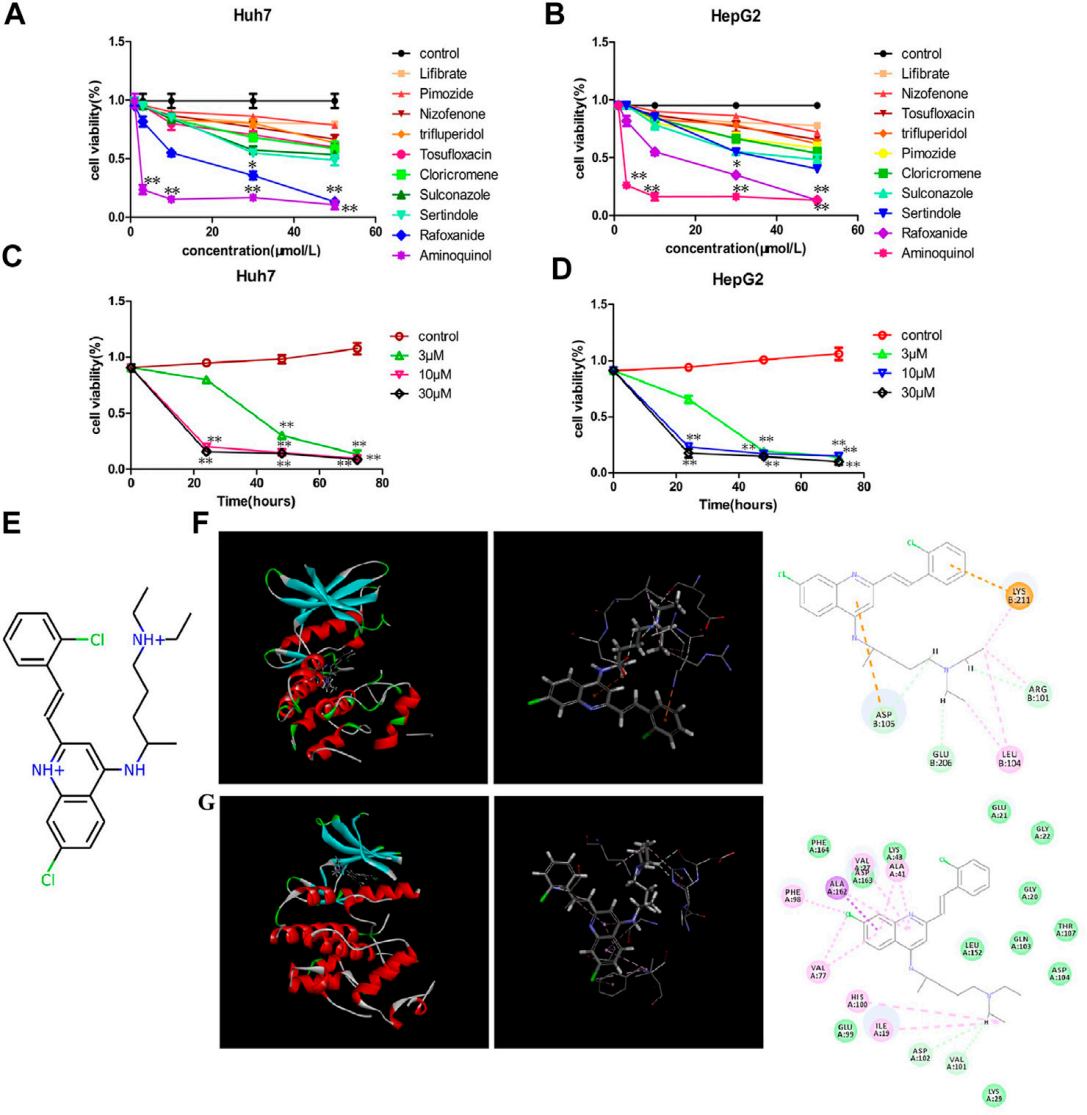
The effect of aminoquinol on the cell viability in human hepatocarcinoma Huh7 and HepG2 cell lines **(A)** The effect of ten candidate compounds on the cell viability in Huh7 cells and **(B)** in HepG2 cells **(C)** Time-response curves for different doses of aminoquinol in Huh7 cells and **(D)** in HepG2 cells **(E)** The predicted two-dimensional chemical structure of aminoquinol **(F)** Computer docking analysis of the interaction of aminoquinol with CDK4 and **(G)** with CDK6 *, ** indicate significant difference at *p* < 0.05, *p* < 0.01, respectively.

These ten compounds (Lifibrate, Nizofenone, Pimozide, TRIFLUPERIDOL, Tosufloxacin, Cloricromene, Sulconazole, Sertindole, Rafoxanide and Aminoquinol) were tested for their ability to decrease the viability of Huh7 (Figure 1A) and HepG2 (Figure 1B) cells. Aminoquinol was found to be the most effective drug in both cell lines. Aminoquinol reduced cell viability significantly (*p* < 0.05) in a dose and time dependent manner (Figure 1C and Figure 1D), with IC50 value equal to 1.47 μM for HepG2 and 2.13 μM for Huh7 cells. The predicted two-dimensional chemical structure of aminoquinol is shown in Figure 1E. Aminoquinol was stabilized through specific interactions such as hydrogen bonding, as well as nonspecific interactions such as hydrophobic interactions with residues in the drug-binding pocket of CDK4 and CDK6. Computer docking analysis predicted that aminoquinol interact with CDK4 (Figure 1F) through three hydrogen bonds with ARG101, ASP105 and GLU206, two π−π stacking with ASP105 and LYS211, and one hydrophobic contact with LEU104. It interacts with CDK6 (Figure 1G) through four hydrophobic contacts with ILE19, ALA41, VAL77 and ALA162, two hydrogen bonds with VAL101 and ASP102, and two salt bridges with VAL77 and PHE98.

### Aminoquinol Caused Cell Cycle Arrest in G1 Phase and Induced Cell Apoptosis

**FIGURE 2:**
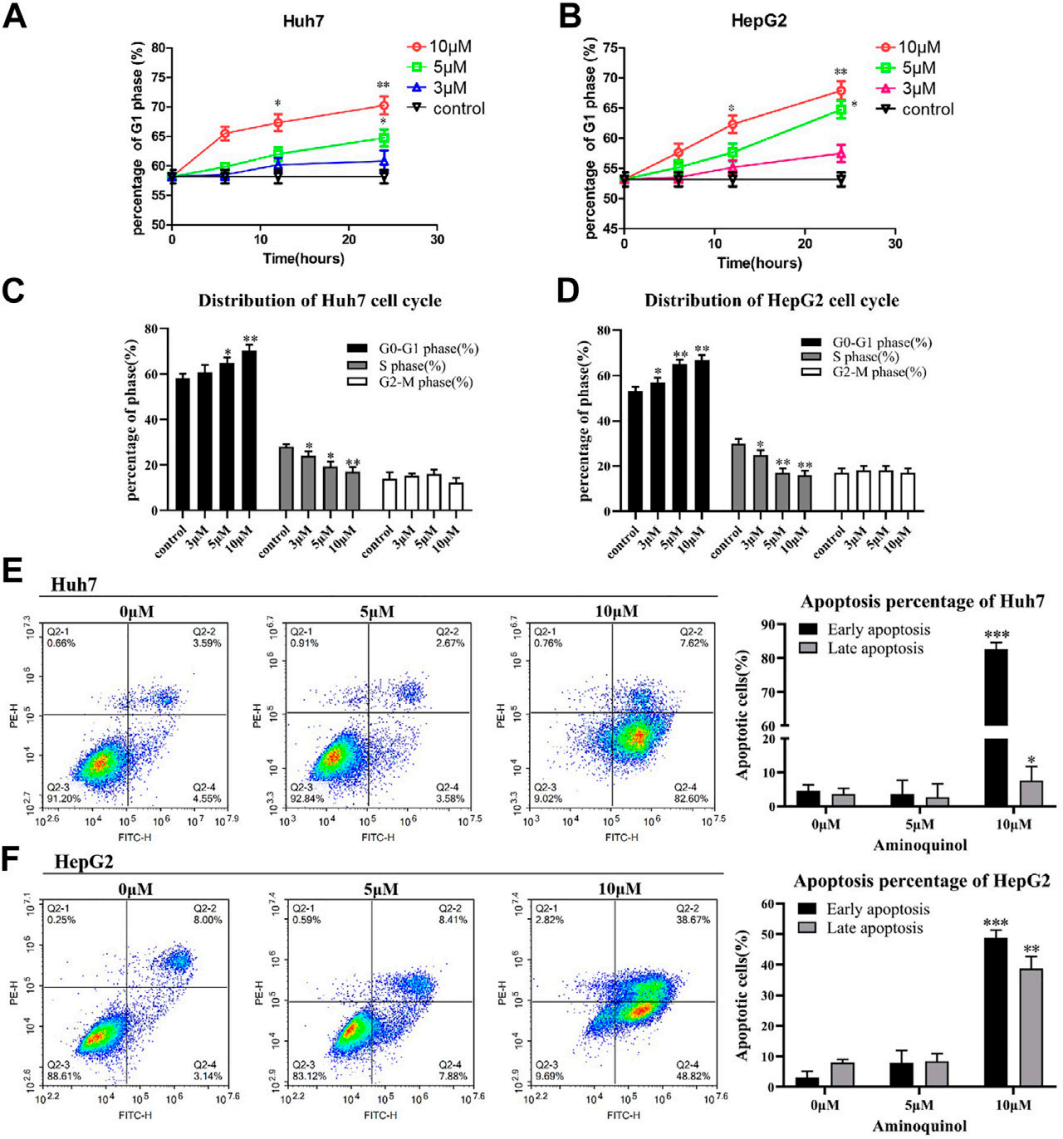
Aminoquinol treatment caused cell cycle G1 arrest and induced apoptosis in Huh7 and HepG2 cells **(A)** Aminoquinol (3, 10 and 30 μM) treatment induced the accumulation of cells in G1 phase in a dose-and time-dependent manner in Huh7 and **(B)** HepG2 cells **(C)** At 24 h after treatment, aminoquinol significantly (*p* < 0.05) increased cell populations in the G1 phase and decreased the cell populations in S-phase in Huh7 and **(D)** HepG2 cells **(E)** At 48 h after treatment. aminoquinol (10 μM) significantly increased the percentage of early and late apoptosis in Huh7 and **(F)** HepG2 cells as compared to the control (*p* < 0.05), *, ** indicate significant difference at *p* < 0.05, *p* < 0.01, respectively.

To assess the effects of aminoquinol on cell-cycle progression, cells were treated with aminoquinol (3, 5, 10 μM) for 6, 12 or 24 h, and its effects on the cell cycle profile were determined by flow cytometry. Aminoquinol induced the accumulation of cells in G1 phase as compared to the control group in a dose-and time-dependent manner in Huh7 (Figure 2A) and HepG2 (Figure 2B) cells. At 24 h after treatment, it significantly (*p* < 0.05) increased cell populations in the G1 phase and decreased the cell populations in S-phase in both Huh7 and HepG2 cells (Figure 2C and Figure 2D). We also investigated whether aminoquinol could induce cell apoptosis as tumors often re-grow after stop dosing with cell cycle inhibitor. As shown in Figure 2E and Figure 2F, aminoquinol (10 μM) significantly increased the percentage of both early and late apoptosis in Huh7 (Figure 2E) and HepG2 (Figure 2F) cells as compared to the control (*p* < 0.05), at 48 h after treatment.

### Aminoquinol Treatment Decreased the Expression of CDK4/6, Rb, CyclinD, Pho-Rb, Pho-CDK4/6, but Not CDK2 in HepG2 and Huh7 Cells

**FIGURE 3:**
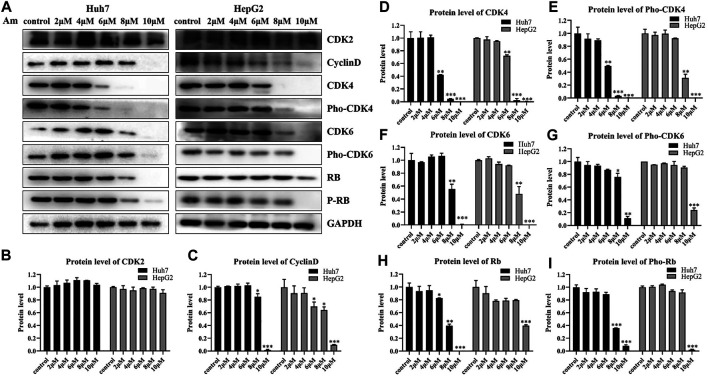
Aminoquinol treatment decreased the expressions of CDK4/6, Rb, cyclinD, pho-Rb, pho-CDK4/6, but not CDK2 in HepG2 and Huh7 cells **(A)** The western blot analysis of Huh7 and HepG2 cells treated with different concentrations of aminoquinol (0, 2, 4, 6, 8, 10 μM) for 6 h **(B–I)** The quantified data of protein expressions for **(B)** CDK2, **(C)** CyclinD, **(D)** CDK4, **(E)** pho-CDK4, **(F)** CDK6, **(G)** pho-CDK6, **(E)** Rb and **(I)** pho-Rb. Data were presented as means ± standard deviation.*p < 0.05, **p < 0.01, ***p < 0.001, significantly different from the control group.

To support the notion that aminoquinol is a CDK4/6 inhibitor, western blot analysis (Figure 3A) was used to verify the effects of aminoquinol (0, 2, 4, 6, 8, 10 μM) on the expressions of key proteins involved in cell cycle progression in Huh7 and HepG2 cells after 6 h of treatment. Amninoquinol dose-dependently decreased the expressions of CDK4 (Figure 3D), pho-CDK4 (Figure 3E), CDK6 (Figure 3F), pho-CDK6 (Figure 3G), cyclinD (Figure 3C), Rb (Figure 3H), and pho-Rb (Figure 3I), while it did not cause significant change in CDK2 (Figure 3B) expression. Quantitatively, aminoquinol appears to be most effective for reducing the expression of CDK4 (Figure 3D). These results suggested that amninoquinol inhibited CDK4/6 phosphorylation, reduced their complex with cyclinD, subsequently led to the reduction of Rb phosphorylation and the activation of E2F, and effectively inhibited the transition of G1 to S phase.

### The Synergy of Aminoquinol Combined With 5-Fu, Sorfafenib and LY294002 in Reducing Cell Viability

**FIGURE 4:**
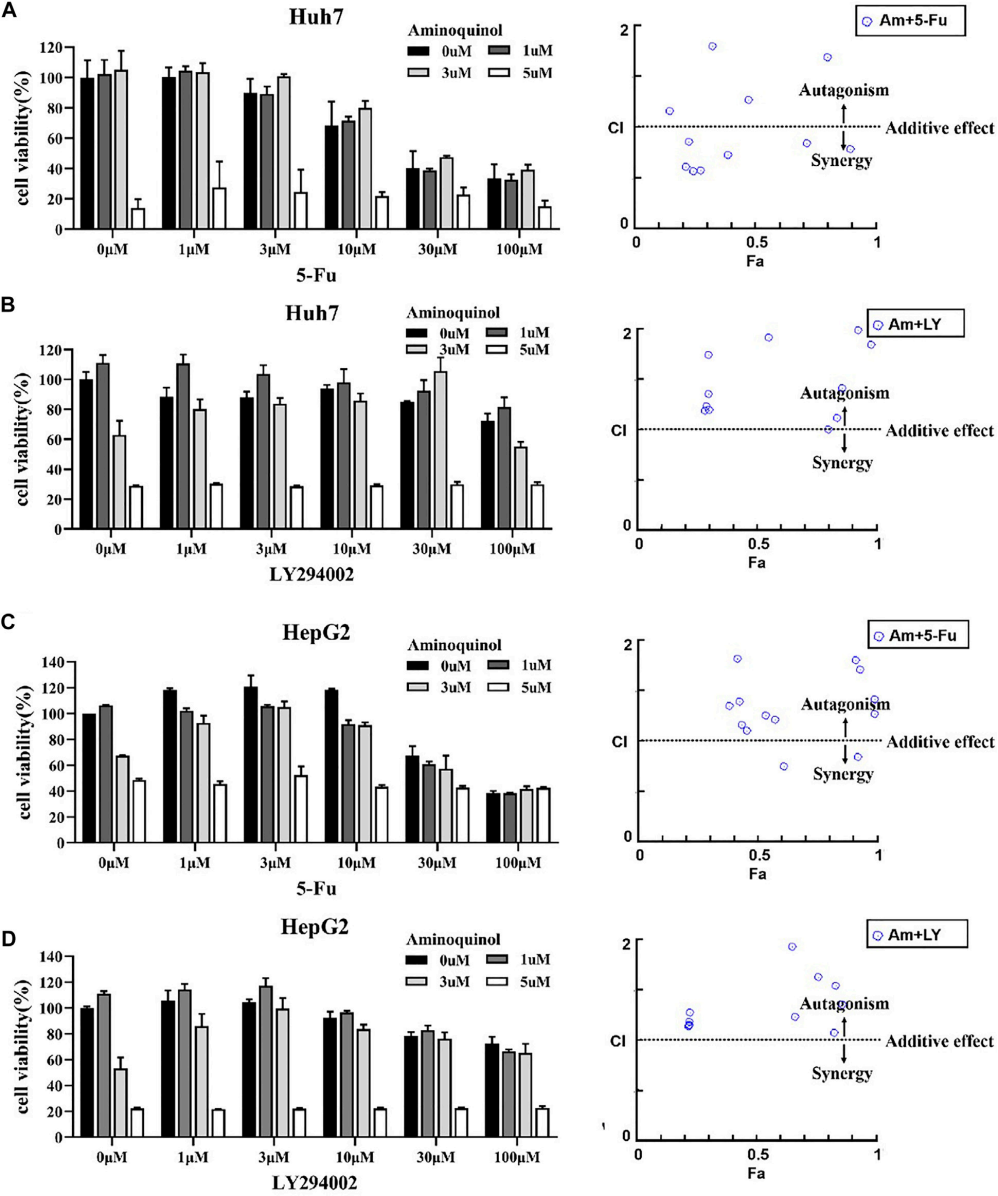
The antagonistic or synergistic effect of combinating aminoquinol with 5-Fu, sorfafenib, and LY294002 in reducing cell viability in HCC cells **(A, B)**, left figures) Huh7 cells or **(C, D)**, left figures) HepG2 cells were treated for 72 h with aminoquinol (0, 1, 3, 5 μM) in combination with 5-Fu (0, 1, 3, 10, 30, 100 μM), or the PI3K inhibitor LY294002 (0, 1, 3, 10, 30, 100 μM), and cell viability determined **(A–D)**, right figures) The fraction affected (Fa)-combination index (CI) plots of aminoquinol in combination with 5-Fu or LY294002. The combined effects and the specific combination index (CI) were analyzed by CompuSyn software. CI > 1 indicated the antagonistic effect, CI = 1 indicated additive effect, and CI < 1 indicated synergistic effect.

The actions of CDK inhibitors on cell cycle arrest requires an intact functional RB. We found that aminoquinol could effectively reduce the viability of the Rb negative Hep3B cells (IC50 = 5.34 μM), suggesting that it has other anti-cancer mechanisms in addition to CDK4/6. To identify the additional mechanisms, Chou-Talalay method were used to evaluate the antagonistic or synergistic effect of aminoquinol (0, 1, 3, 5 μM) in combination with 5-Fu (0, 1, 3, 10, 30, 100 μM), the multi-kinase inhibitor sorfafenib (0, 1, 3, 10, 30, 100 μM), and the PI3K inhibitor LY294002 (0, 1, 3, 10, 30, 100 μM). Briefly, Huh7 (Figures 4A, B) and HepG2 (Figures 4C, D) cells were plated in 96-well plates (5 × 10^3^cells/well), and treated with indicated concentrations of aminoquinol plus 5-Fu, sorafenib or LY294002 for 72 h. Cell viability was determined by the CCK-8 assay (Figures 4A–D left figure). The specific combination index (CI) values of the drug combination are shown in Supplementary Table 2–7. In aminoquinol and 5-Fu (Figure 4A, Figure 4C, right figure) or sorafenib (Supplementary Figure 2A, B, right figure) combination treatments, most of the fraction affected-combination index (Fa) and CI plot, the CI values were less than 1, suggesting a weak synergistic effect. Importantly, we found that the fraction affected-combination index (Fa) and CI plot in the aminoquinol and LY294002 combination, indicated a clear antagonistic effect with all of the CI > 1 in Huh7 (Figure 4B, right figure) and HepG2 (Figure 4D, right figure) cells. These results strongly suggested that aminoquinol itself may possess PI3K inhibitor activity.

### Aminoquinol Reduced the Expressions of Key Proteins in the PI3K/AKT/mTOR Pathway

**FIGURE 5:**
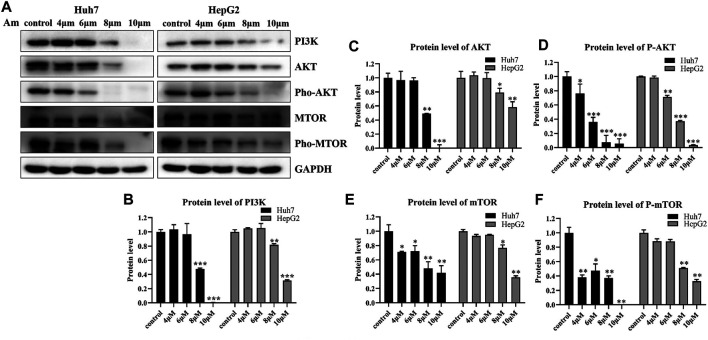
Aminoquinol reduced the expressions of key proteins in the PI3K/AKT/mTOR pathway **(A)** Western blot analysis of Huh7 and HepG2 cells treated with indicated concentrations of aminoquinol for 6 h **(B–F)** The quantified data of **(B)** PI3K, **(C)** AKT, **(D)** p-AKT, **(E)** mTOR, and **(F)** p-mTOR. Data were presented as means ± standard deviation. *p < 0.05, **p < 0.01, ***p < 0.001, significantly different from the control group.

To validate the effect of aminoquinol on the PI3K signal pathway, western blot analysis (Figure 5A) was conducted. Our results showed that aminoquinol (0, 4, 6, 8, 10 μM) treatment significantly and dose dependently decreased the protein expressions of PI3K (Figure 5B), AKT (Figure 5C), pho-AKT (Figure 5D), mTOR (Figure 5E), and pho-mTOR (Figure 5F), in Huh7 and HepG2 cells. Meanwhile, it had no significant effect on the expressions of Ras, Raf, MEK and ERK (Supplementary Figure 3). These results were consistent with what is expected of an PI3K/AKT inhibitor.

### Thermal Proteome Profiling Analysis Suggested the Binding of Aminoquinol With CDK4/6, PI3K and AKT in Huh7 Cells

**FIGURE 6:**
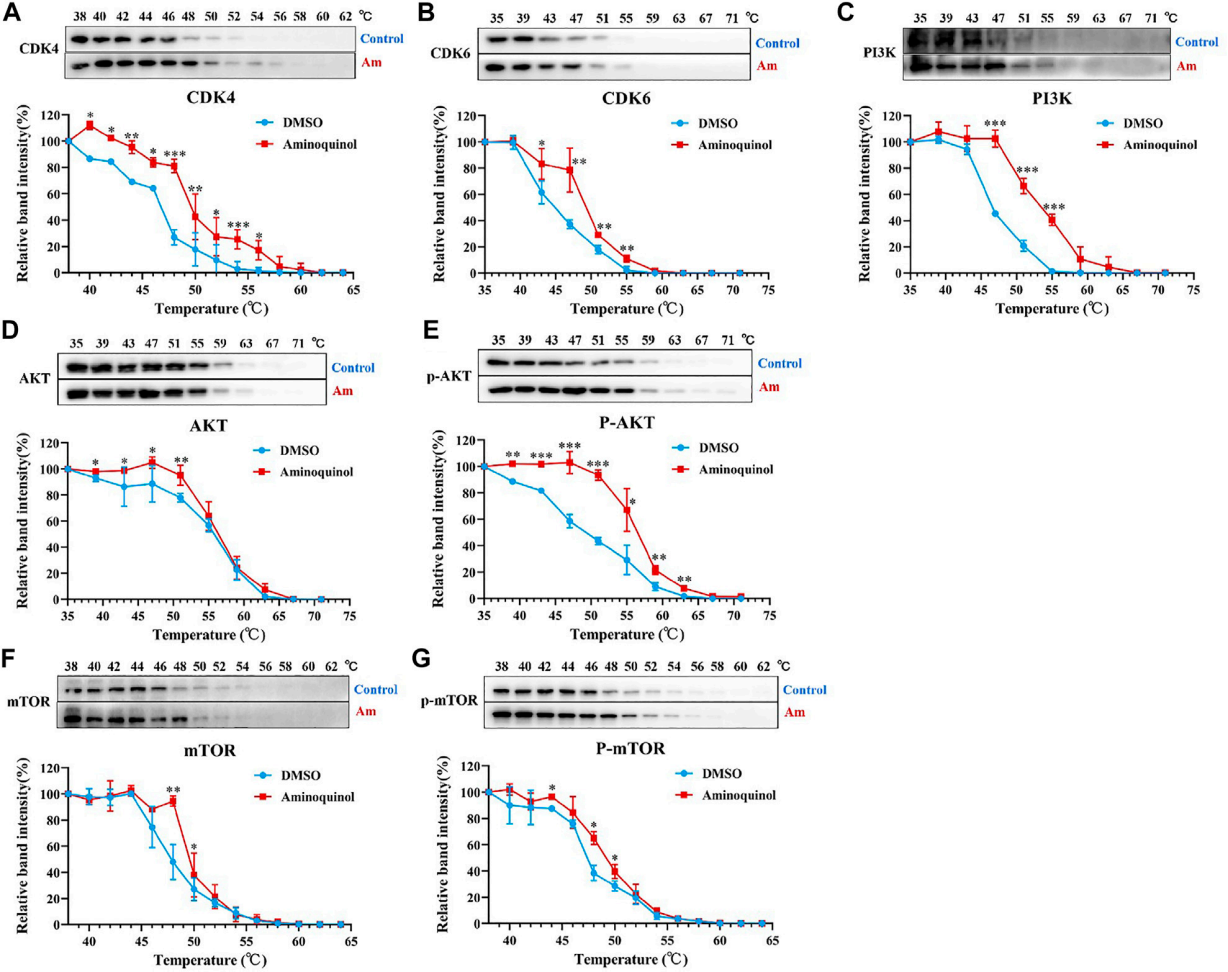
Thermal proteome profiling (TPP) analysis suggested the binding of aminoquinol with CDK4/6, PI3K and p-AKT in Huh7 cells. Huh7 cell lysate was treated with aminoquinol (100 μM) or vehicle (DMSO) for 20 min and incubated at temperatures ranging from 35 to 75°C. Proteins from each fraction were analyzed via western blotting. Quantification was based on the intensity of bands. Thermal denaturation curves were obtained from all proteins in control (blue) and aminoquinol-treated (red) samples **(A–C, E)** The representative pictures of western blotting and thermal denaturation curves of **(A)** CDK4, **(B)** CDK6, **(C)** PI3K and **(E)** p-AKT, indicating thermal stabilization **(D, F–G)** The thermal stability of **(D)** AKT, **(F)** mTOR and **(G)** p-mTOR, indicating weak increases in thermal stability. All experiments were performed in triplicate, and data were given as the AVE ± SEM. *p < 0.05, **p < 0.01, ***p < 0.001, significantly different from the control group.

To further validate the molecular targets of aminoquinol, thermal proteome profiling (TPP) analysis were performed. Huh7 cells were lysed and treated for 20 min with vehicle (DMSO) or 100 μM aminoquinol to ensure a complete saturation of ligand binding and maximizes the shift in detected protein denaturation, and then heated at temperatures ranging from 35 to 75°C to denature and precipitate unbound proteins, as protein which bound to aminoquinol will be more stable and could not be precipitated at high temperature. The thermally aggregated proteins were then removed by centrifugation, and soluble protein fraction which contains ligand-bound proteins were collected and analyzed by western blot analysis. As shown in Figure 6, aminoquinol significantly improved the thermal stability of its main targets CDK4 (Figure 6A), CDK6 (Figure 6B), PI3K (Figure 6C), and pho-AKT (Figure 6E), suggesting direct bindings of aminoquinol and these proteins. The thermal stability of AKT (Figure 6D), mTOR (Figure 6F), and pho-mTOR (Figure 6G) showed weak increases, suggesting weak binding or indirect interaction with aminoquinol (Franken et al., 2015).

### Aminoquinol Reduced the Tumor Growth *in vivo* in BALB/C Nude Mice Xenografted With Huh7 Cells

**FIGURE 7:**
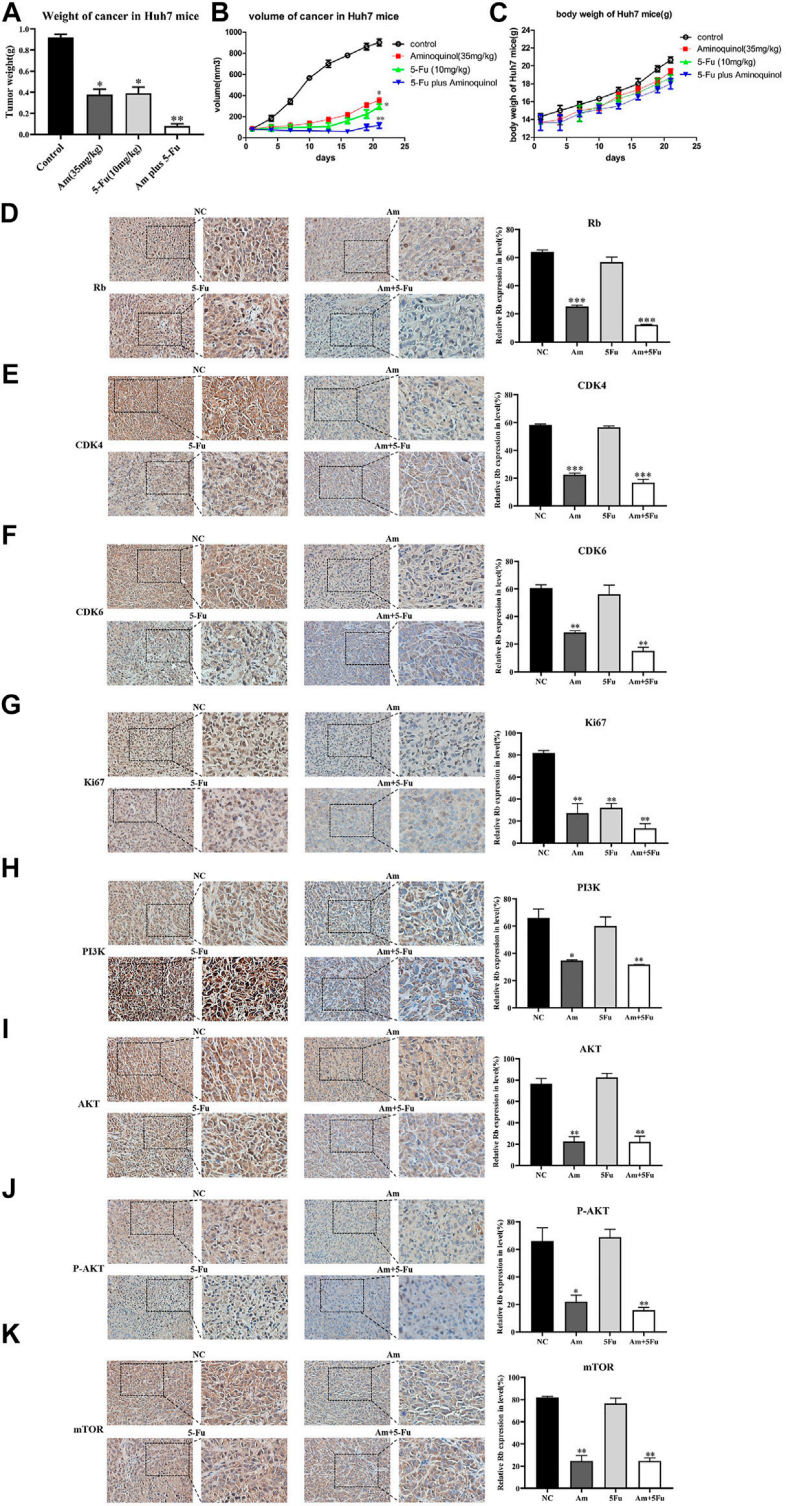
Aminoquinol treatment inhibited tumor growth *in vivo* in BALB/C nude mice xenografted with Huh7 cells. BALB/C nude mice xenografted with Huh7 cells were treated with aminoquinol (35 mg/kg), 5-Fu (10 mg/kg), aminoquinol (35 mg/kg) plus 5-Fu (10 mg/kg) and PBS for 21 days daily by intraperitoneal injection **(A)** Tumor weight at 21 days after treatment **(B)** Tumor volumes during the experiment **(C)** The body weight of the mice during the experiment **(D–K)** The representative pictures and quantified data of immunohistochemistry staining of the xenografted tumor tissues for the expressions of **(D)** Rb, **(E)** CDK4, **(F)** CDK6, **(G)** ki67, **(H)** PI3K, **(I)** AKT, **(J)** P-AKT, and **(K)** mTOR. Data are expressed as the mean ± SD. *p < 0.05, **p < 0.01, ***p < 0.001, significantly different from the control PBS treatment group.

The *in vivo* anti-tumor activity of aminoquinol were evaluated in BALB/C nude mice xenografted with Huh7 cells. Huh7 cells were subcutaneously injected into the armpits of BALB/C nude mice. When the tumors grew to 80–100 mm^3^ (7 days after inoculation), mice were randomly divided into four groups (5 mice/group), and treated with daily intraperitoneal administration of 1) control (PBS), 2) aminoquinol (35 mg/kg, i. p.), 3) 5-Fu (10 mg/kg, i. p.), and aminoquinol (35 mg/kg, i. p.) plus five- Fu (10 mg/kg, i. p.) for 21 days, and the tumor volume recorded. At the end of the experiment, mice were sacrificed by cervical dislocation, tumor tissues excised, weighed, images captured, and immunohistochemistry analysis performed. Aminoquinol and 5-Fu treatments significantly reduced tumor weight (Figure 7A) and tumor volume (Figure 7B), with comparable curative effects. Of note, administration of combined aminoquinol plus 5-Fu produced the highest therapeutic effect. At the same time, all treatments had no significant effect on the body weight of the mice during the whole experiment (Figure 7C). Immunohistochemistry staining of tumor tissues showed that aminoquinol treatment significantly reduced the expressions of Rb, CDK4, CDK6, PI3K, pho-AKT and pho-mTOR (Figures 7D–K), as compared to the control group. The 5-Fu treatment did not produce any obvious effect.

### Clinical Significance

**FIGURE 8:**
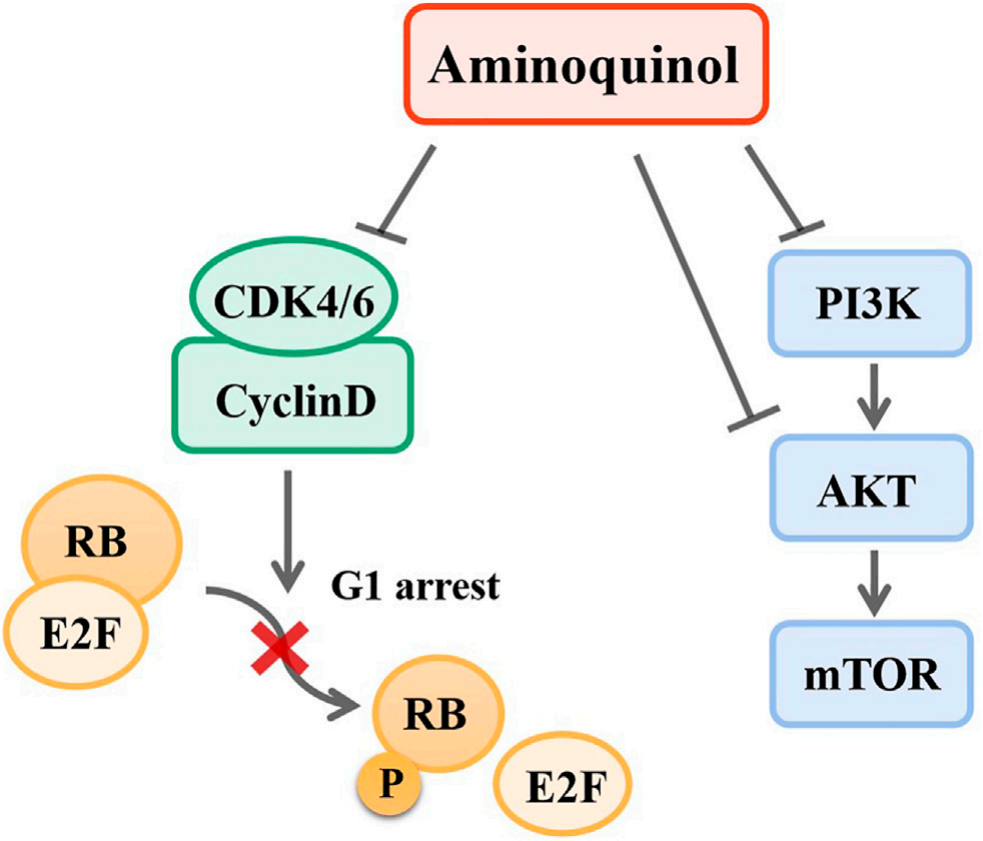
Mechanisms of aminoquinol. Aminoquinol inhibited CDK4/6 phosphorylation and the complex with cyclinD, blocked the phosphorylation of RB and the releases of pRB via its association with transcription factor E2F, and subsequently inhibited the cell cycle to proceed from G1 to S-phase. In addition, aminoquinol also suppressed the signal pathway PI3K/AKT/mTOR, therefore inhibited cell proliferation and promoted apoptosis.

Taken together, our results suggested that aminoquinol acted through direct binding to CDK4/6 and PI3K/p-AKT (Figure 8). We analyzed the expressions of CDK4/6 and PI3K/AKT/mTOR in normal human tissues and liver cancer patient tissues through ualcan. As shown in Supplementary Figure 1A, B, in liver cancer tissues, the expressions of CDK4 (p = 1E-12) and CDK6 (*p* = 5.74E-14) were significantly increased. Kaplan-Meier analysis indicated that only elevated expression of CDK4 (Supplementary Figure 1C) is significantly associated with reduced overall patient survival (OS) (*p* = 6.20E-07), but there was no significant difference in CDK6 (Supplementary Figure 1D). For the PI3K/AKT/mTOR pathway, significantly elevated expression of the PI3K (*p* = 1.62E-12) (Supplementary Figure 1E), AKT (*p* = 0.0049) (Supplementary Figure 1F) and mTOR (*p* = 1E-12) (Supplementary Figure 1G) were observed in liver cancer patient tissues as compared to normal liver tissues. Kaplan-Meier analysis indicated that the elevated expressions of PI3K (*p* = 0.0025) (Supplementary Figure 1H) and AKT (*p* = 0.0025) (Supplementary Figure 1I) are significantly associated with reduced overall survival in liver cancer patients, while mTOR levels had no significant association with survival (Supplementary Figure 1J).

## Discussion

Cell cycle progression is regulated in a sequential and highly organized manner through the interaction of CDKs and cyclins (Malumbres and Barbacid 2009). During the G1 to S phase transition, the cyclinD-CDK4/6 complexes are sequentially activated and the retinoblastoma gene (Rb) is hyper-phosphorylated (Reed 1997; Cobrinik 2005), then releases the transcription factor E2F, which in turn facilitates the transcription of numerous cell cycle-related genes and make cells enter the S phase (Meraldi et al., 1999)**.** The expressions of CDK4/6 are often elevated in many tumors (Yamamoto et al., 1995; Kim et al., 2000; Cohen 2002; Li et al., 2002; Che et al., 2012). Theoretically, CDK inhibitor should be a broad spectrum and effective therapeutic drug for HCC as well as many other cancers. However, the efficacy of CDK4/6 inhibitor to HCC and other cancers are very limited (Sherr et al., 2016; Bollard et al., 2017). The discovery of more effective and less toxic CDK inhibitors, or drugs that targeting both CDKs and other major molecular targets are urgently needed.

Currently, the combination of CDK 4/6 inhibitor palbociclib and fulvestant is FDA approved (Vidula and Rugo, 2016) clinically for the treatment of hormone receptor-positive and HER2-negative metastatic breast cancer (Lin et al., 2005). Palbociclib is a highly selective inhibitor of CDK4 and CDK6 kinases (Fry et al., 2004) in cells with functionally intact RB (Matsushime et al., 1992; Ewen et al., 1993; Kato et al., 1993), but not in RB absent Hep3B and BT549 (Asghar et al., 2015; Sherr et al., 2016). Results from an extensive preclinical study (Bollard et al., 2017) supports the use of palbociclib, alone or in combination with sorafenib, for HCC treatment. The adverse side effects of Palbociclib included neutropenia, infections, fatigue and gastrointestinal toxicity, which limited capacity of Palbociclib for the treatment of HCC (Bollard et al., 2017).

To discover more effective and less toxic CDK inhibitors, we have used computer-aided strategy to discover two CDK2 inhibitors, Adapaline and Fluspirilene for liver cancer and colon cancer (Shi et al., 2015a; Shi et al., 2015b), a CDK4/6 inhibitor Rafoxanide for skin cancer but not very effective for HCC (Figures 1A, B) (Shi et al., 2018). In addition, we have recently identified a CDK2/4/6 triple inhibitor (manuscript in preparation). In this study, we reported that aminoquinol is a new CDK4/6 inhibitor. Furthermore, it also inhibited the PI3K/AKT signal pathways, making it the first reported CDK4/6 plus PI3K/AKT muti-kinase inhibitor. Recent data indicated that the PI3K-AKT-mTOR signaling pathway plays an important role in the self-renewal of cancer stem cells and resistance to chemotherapy or radiation therapy (Park et al., 2020; Hou et al., 2021). Therefore, aminoquinol may be more effective for HCC and other cancers.

Considering the diverse genomic dysregulations observed in HCC, the combination of CDK inhibitor with inhibitors of multiple additional molecular targets should be more effective in the treatment of HCC and other cancers (Sobhani et al., 2019)**.** As the loss of RB constituted the main mechanism of inherent drug resistance and acquired resistance in human liver cancer (Rivadeneira et al., 2010; Bollard et al., 2017). The observation that aminoquinol is equally effective on liver cancer cells which lack RB gene, may make it clinically more effectively against drug resistance.

Furthermore, aminoquinol displayed synergistic effect when used in combination with 5-Fu and sorfafenib (targets VEGFR-1, 2, 3, RET/PTC, and BRAF). (Bollard et al., 2017; Teo et al., 2017; Wang et al., 2018). 5-Fluorouracil (5-Fu) is a widely employed antineoplastic agent that acts as anti-metabolite (Wang et al., 2018; Moracci et al., 2021). Sorafenib has been used as a first-line drug in clinical practice for patients with advanced liver cancer for more than a decade (Bollard et al., 2017; Kudo 2020). Further studies will optimize the best combination of aminoquinol with sorafenib and other targeted drugs to provide additional synergistic efficacy against HCC and other cancers.

At present, aminoquinol is consider as an antiprotozoal drug, mainly used for acute necrotising form of cutaneous leishmaniasis (Shmushovich 1967). Originally used as an anti-plasmodium drug approved by FDA, aminoquinol it is relatively safe in the therapy of human. In our study, we did not observe significant changes in the body weight of the BALB/C nude mice administered (i.p.) with aminoquinol (35 mg/kg) for 21 days. The reported LD50 of aminoquinol equals to 125 mg/kg (i.p.) in mice (Keithly 1982). In comparison, mice received (150 mg/kg, oral) palbociclib showed a slight loss in body weight in the Huh7 xenograft model, with LD50 of Palbociclib in rats equal to 369 mg/kg (oral) (Flaherty et al., 2012). Aminoquinol has been reported to have acute oral toxicity and poor solubility. Further studies are required to improve efficacy and reduce toxicity. These strategies include the use of biodegradable materials as a carrier for targeted drug delivery to cancer cells, or combining with other small molecule drugs to reduce the dosage.

Taken together, as a new CDK4/6 and PI3K/AKT multi-kinase inhibitor, aminoquinol is a potential drug for HCC treatment alone or in combination with 5-Fu, sorafenib and potentially other targeted anti-cancer drugs. Optimization of best treatment combinations for the treatment of HCC and other cancers warrant further investigations.

## Data Availability

The original contributions presented in the study are included in the article/Supplementary Material, further inquiries can be directed to the corresponding authors.
