# Supplementary figures and images for: Discovery of a New CDK4/6 and PI3K/AKT Multiple Kinase Inhibitor Aminoquinol for the Treatment of Hepatocellular Carcinoma
Source: Front Pharmacol. 2021 Jul 15;12:691769. doi: 10.3389/fphar.2021.691769 (PMC8320333; doi:10.3389/fphar.2021.691769)

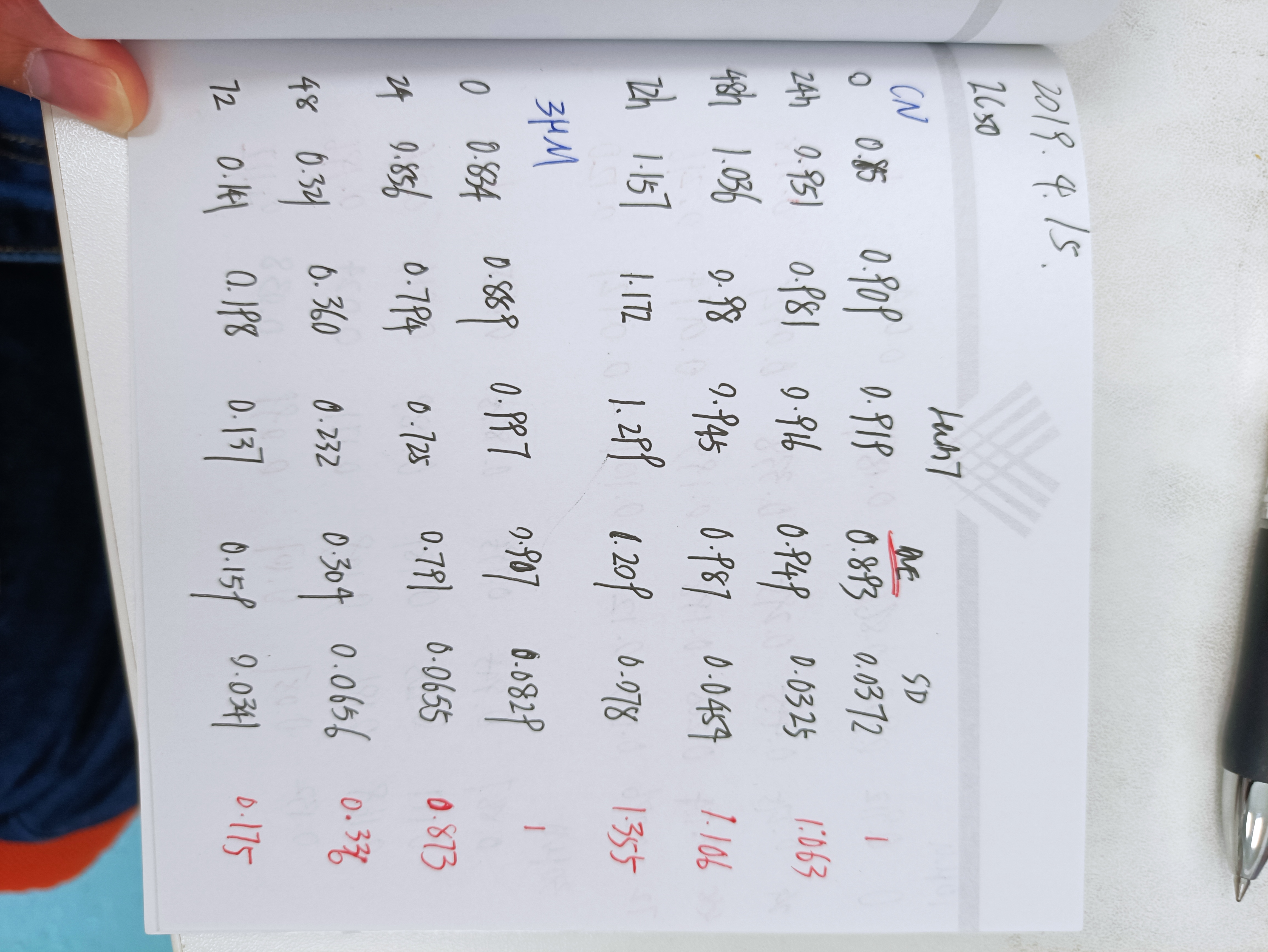

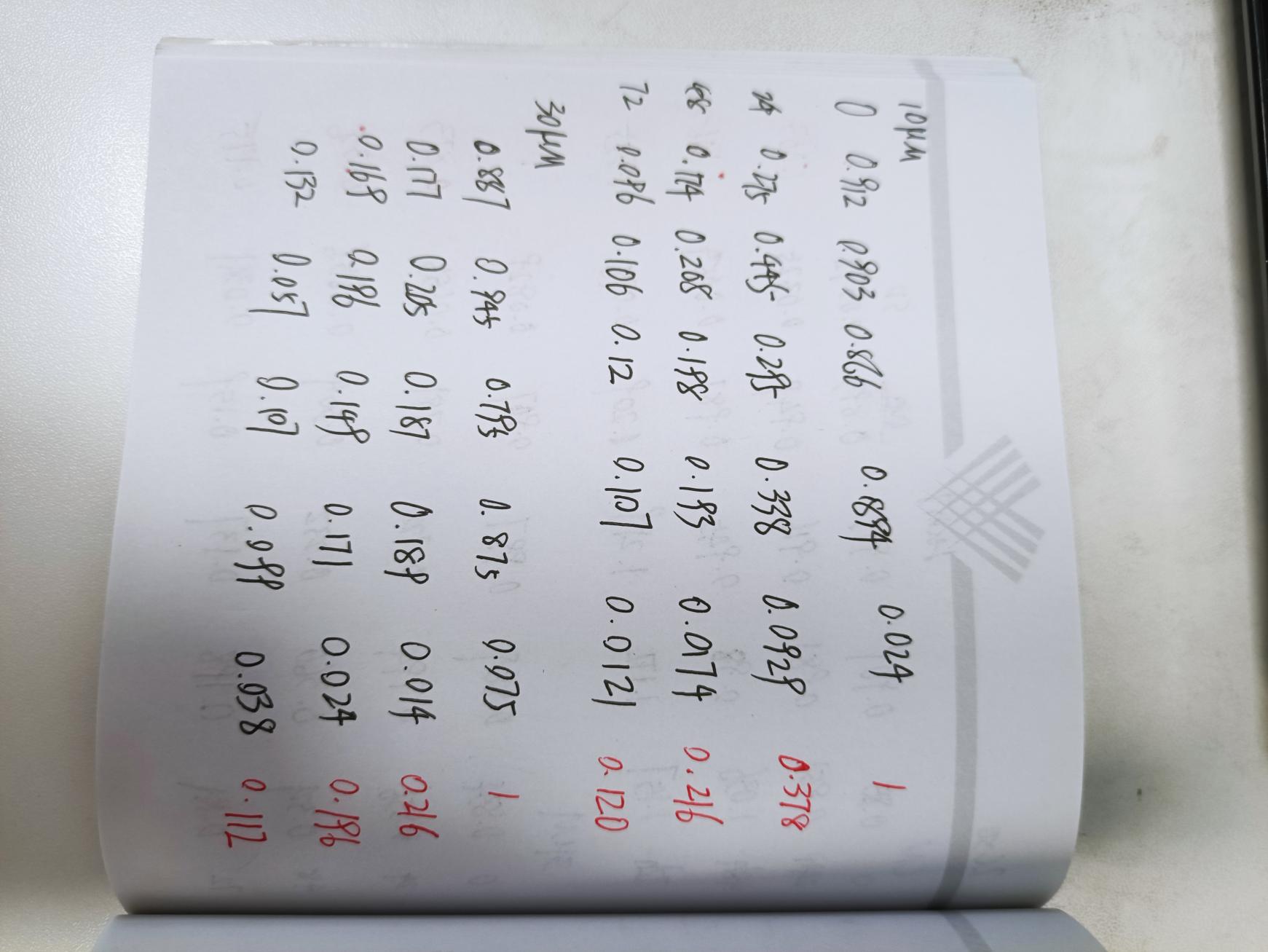


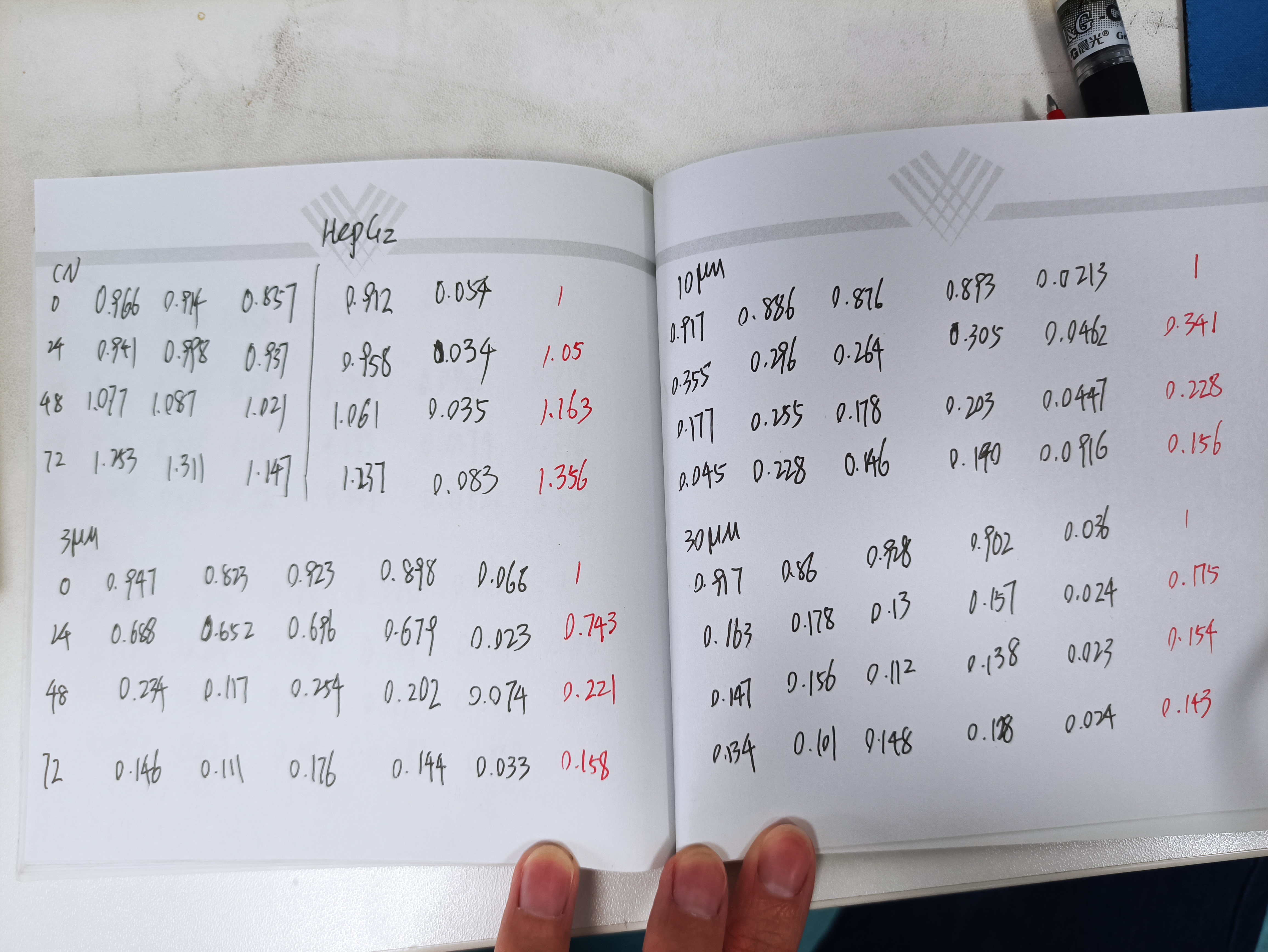

Supplement: Supplementary file 1 [file DataSheet1.ZIP › Original data of Aminoquinol (2)/Original data of Aminoquinol/CCK8.docx]

**Cell cycle**


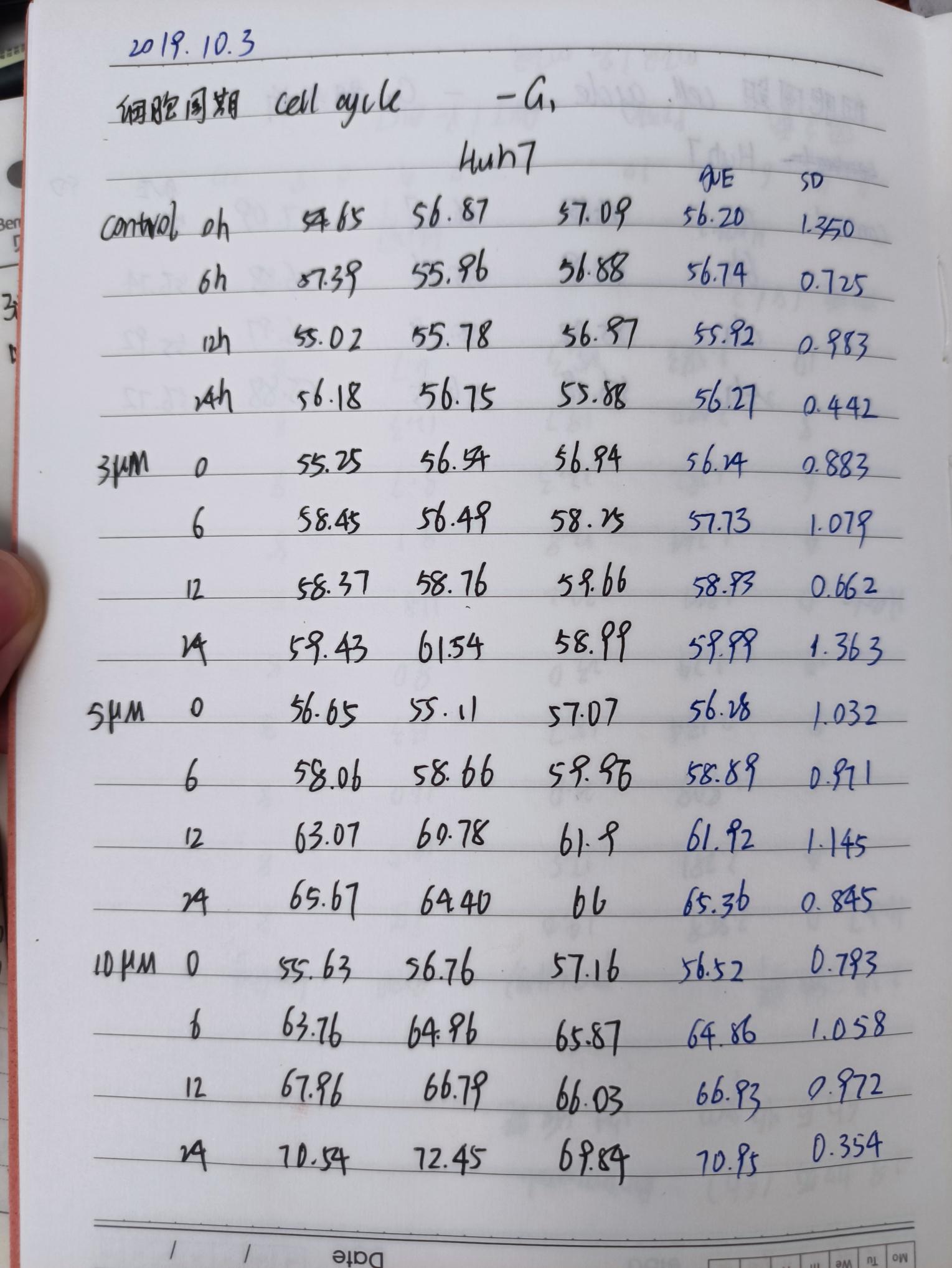

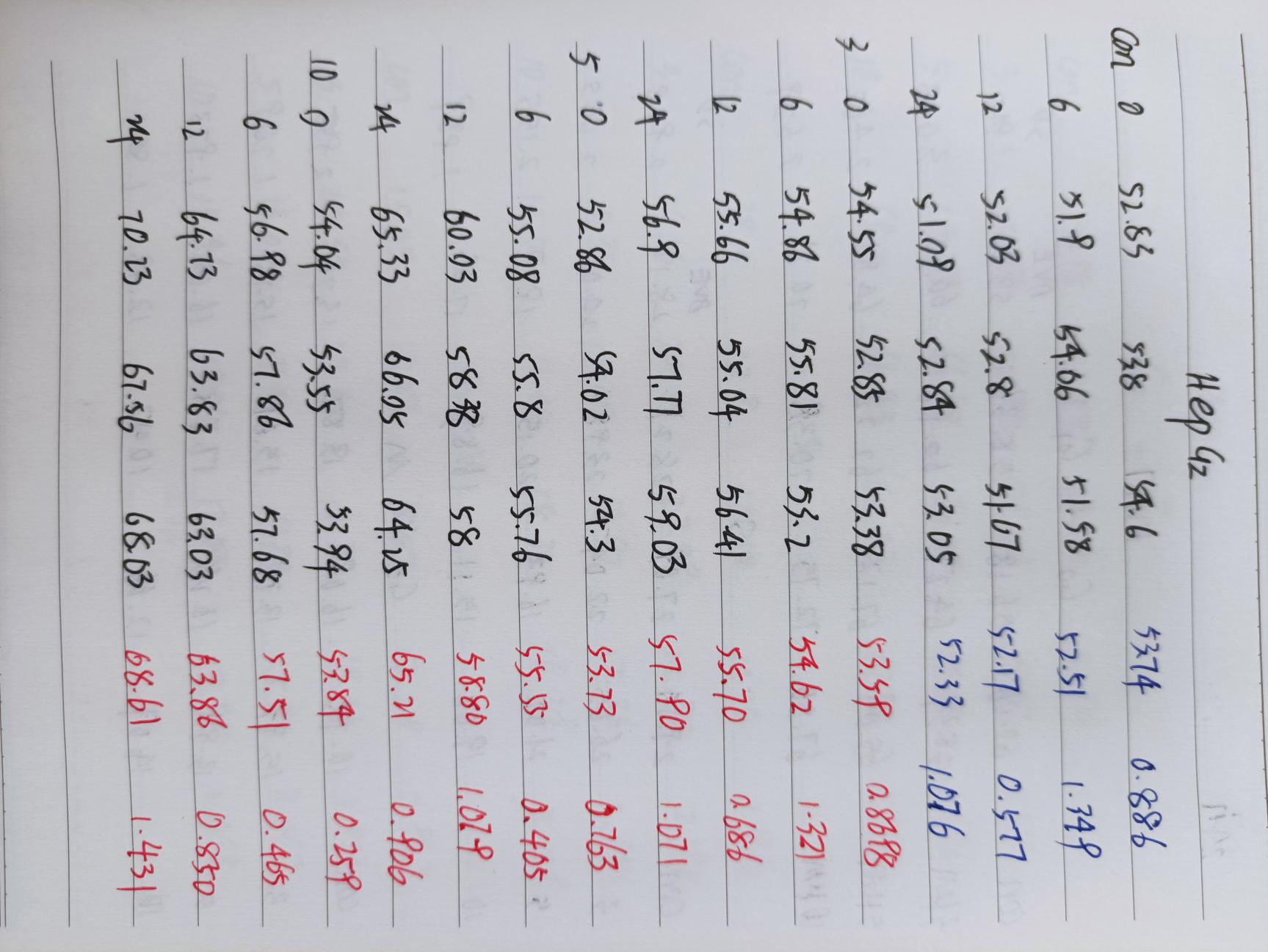

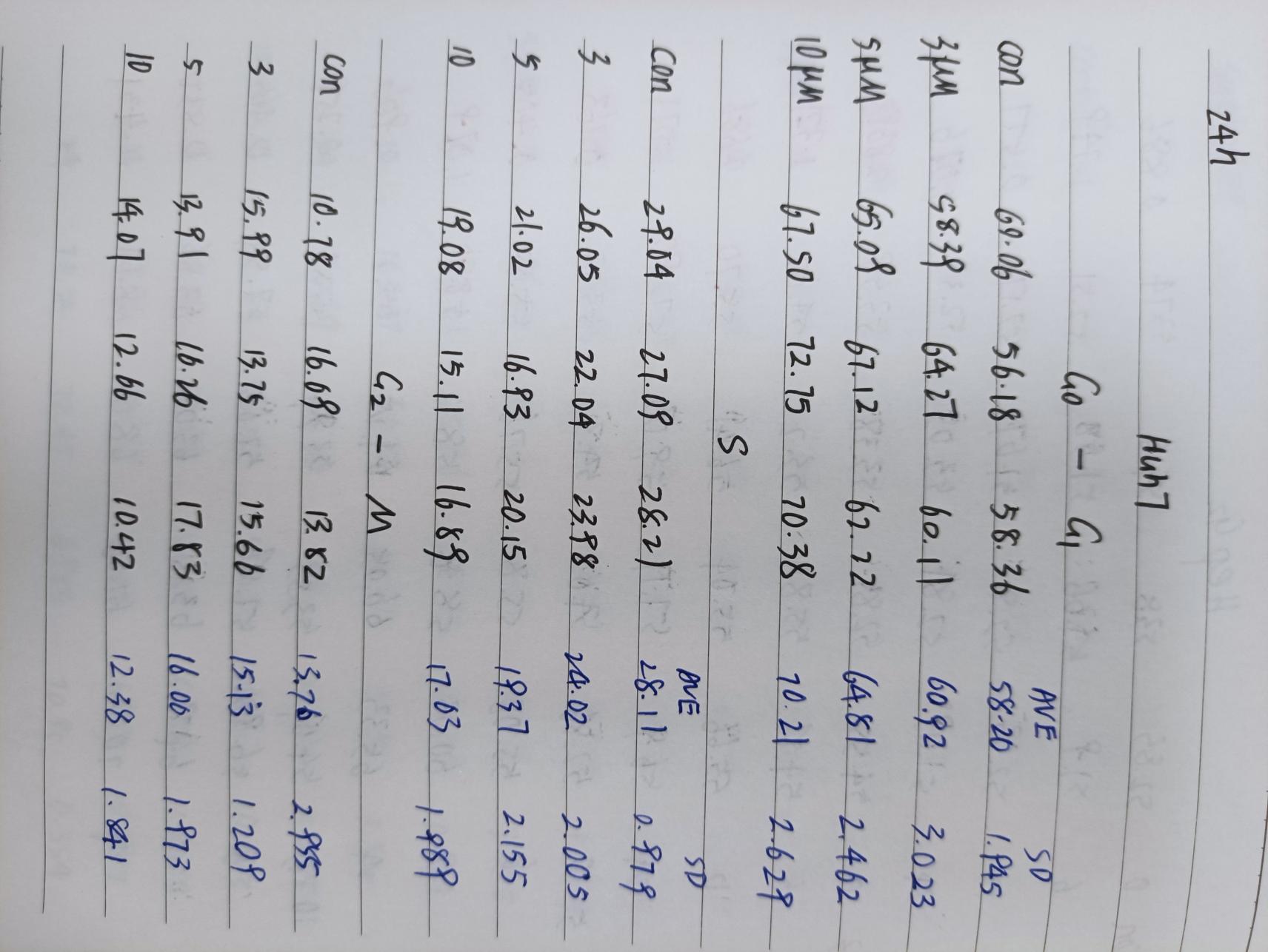


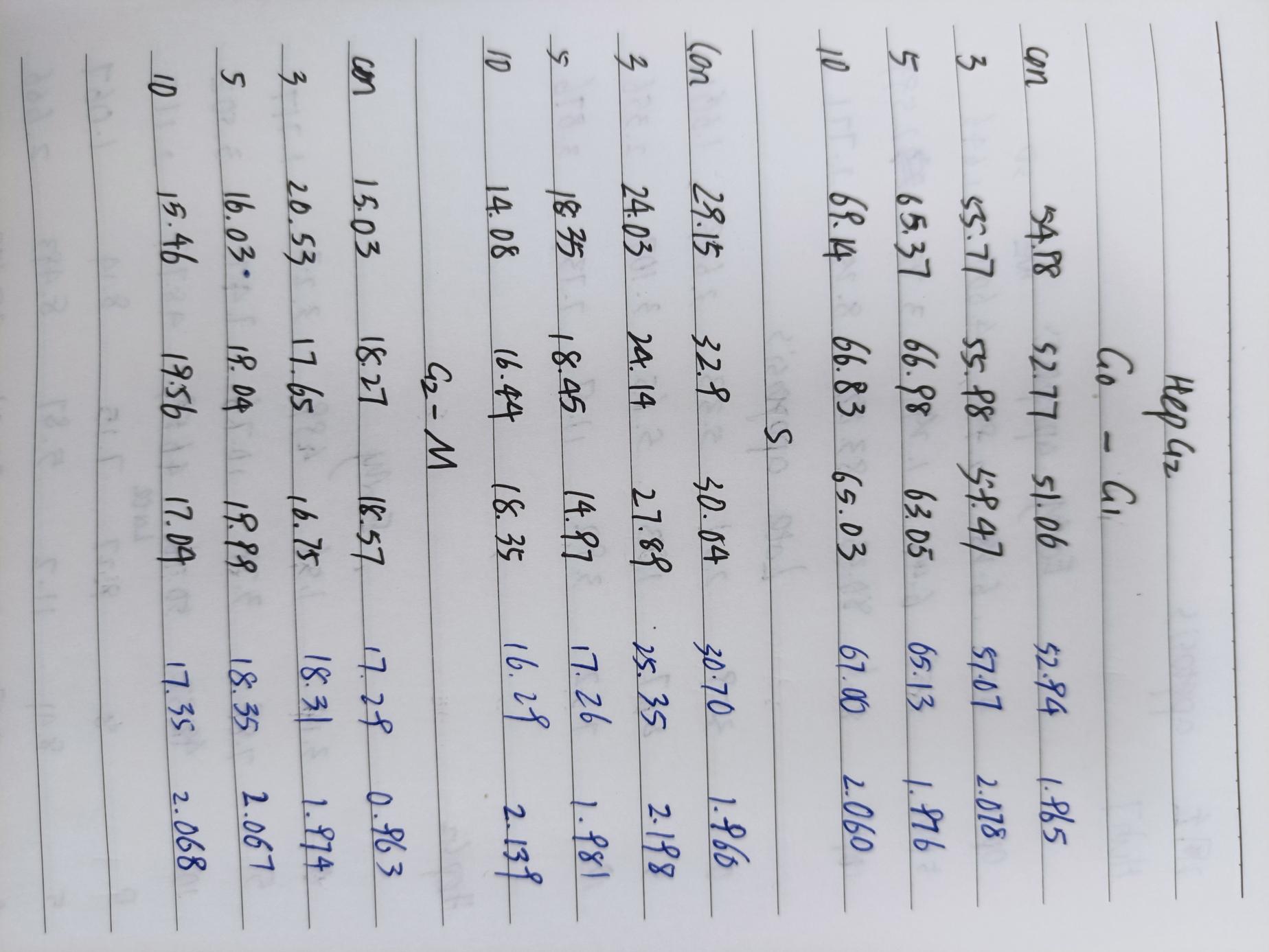


**Apoptosis**

**
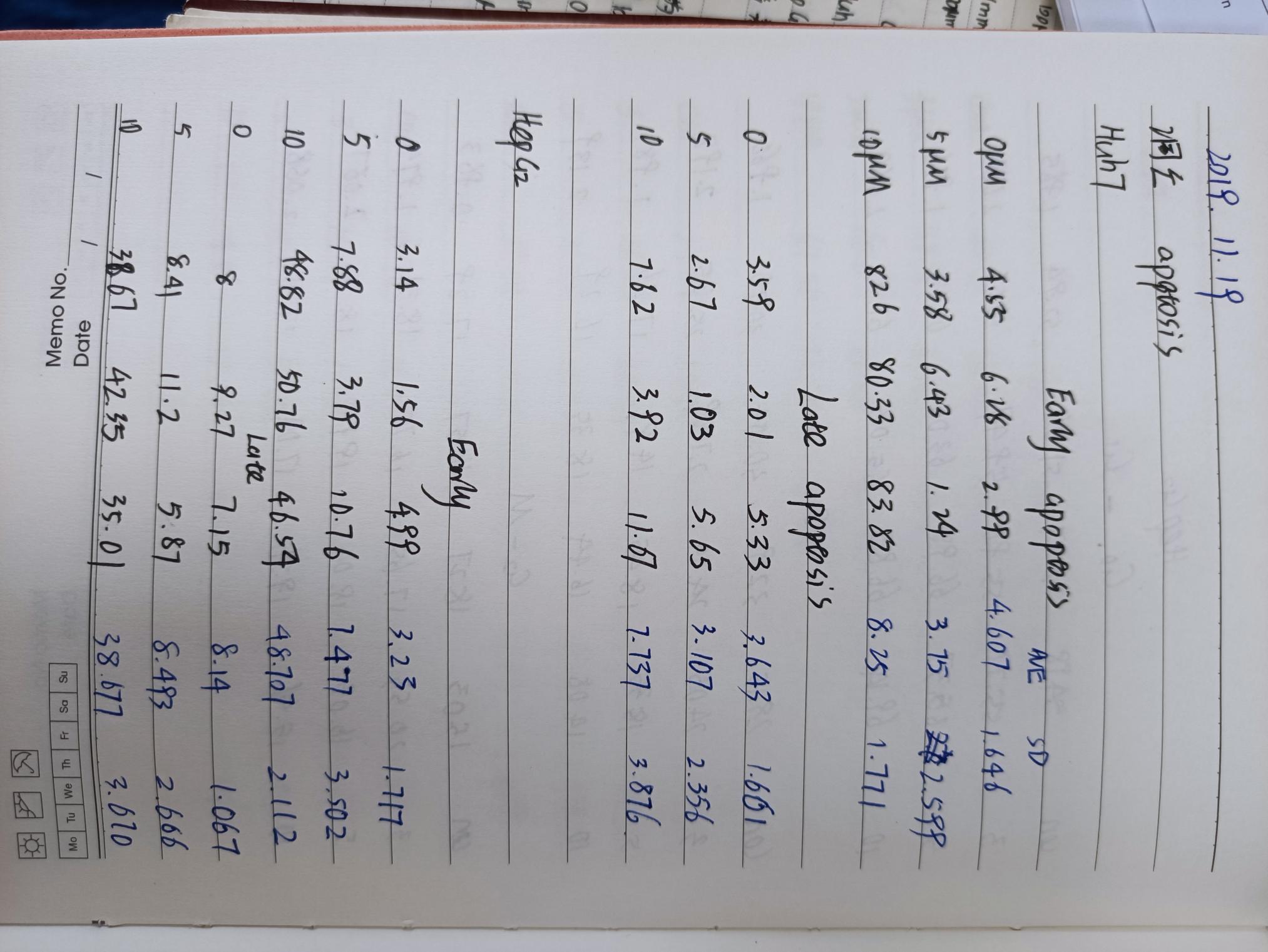
**

Supplement: Supplementary file 1 [file DataSheet1.ZIP › Original data of Aminoquinol (2)/Original data of Aminoquinol/Cell cycle-apoptosis.docx]

**IHC**


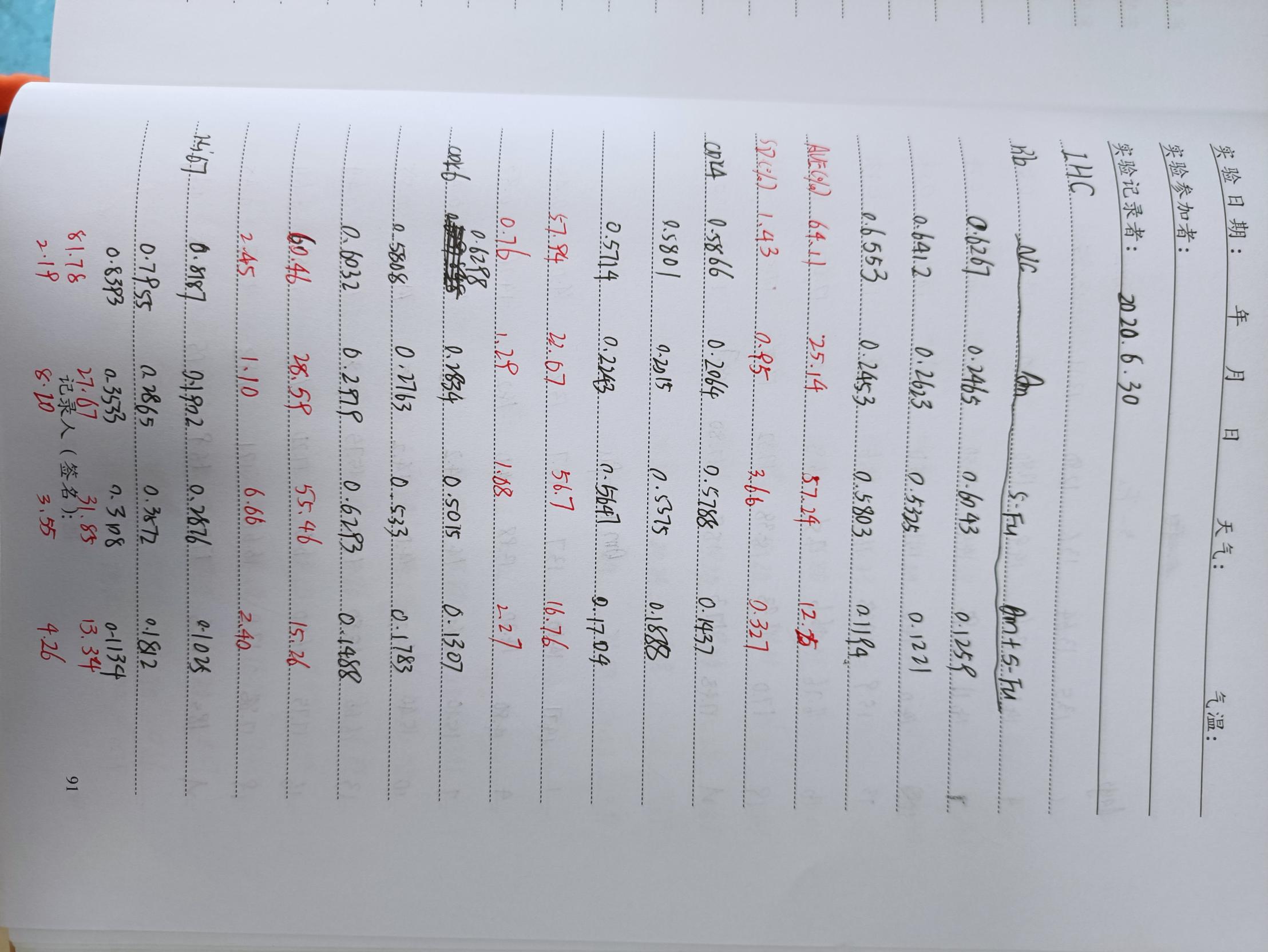

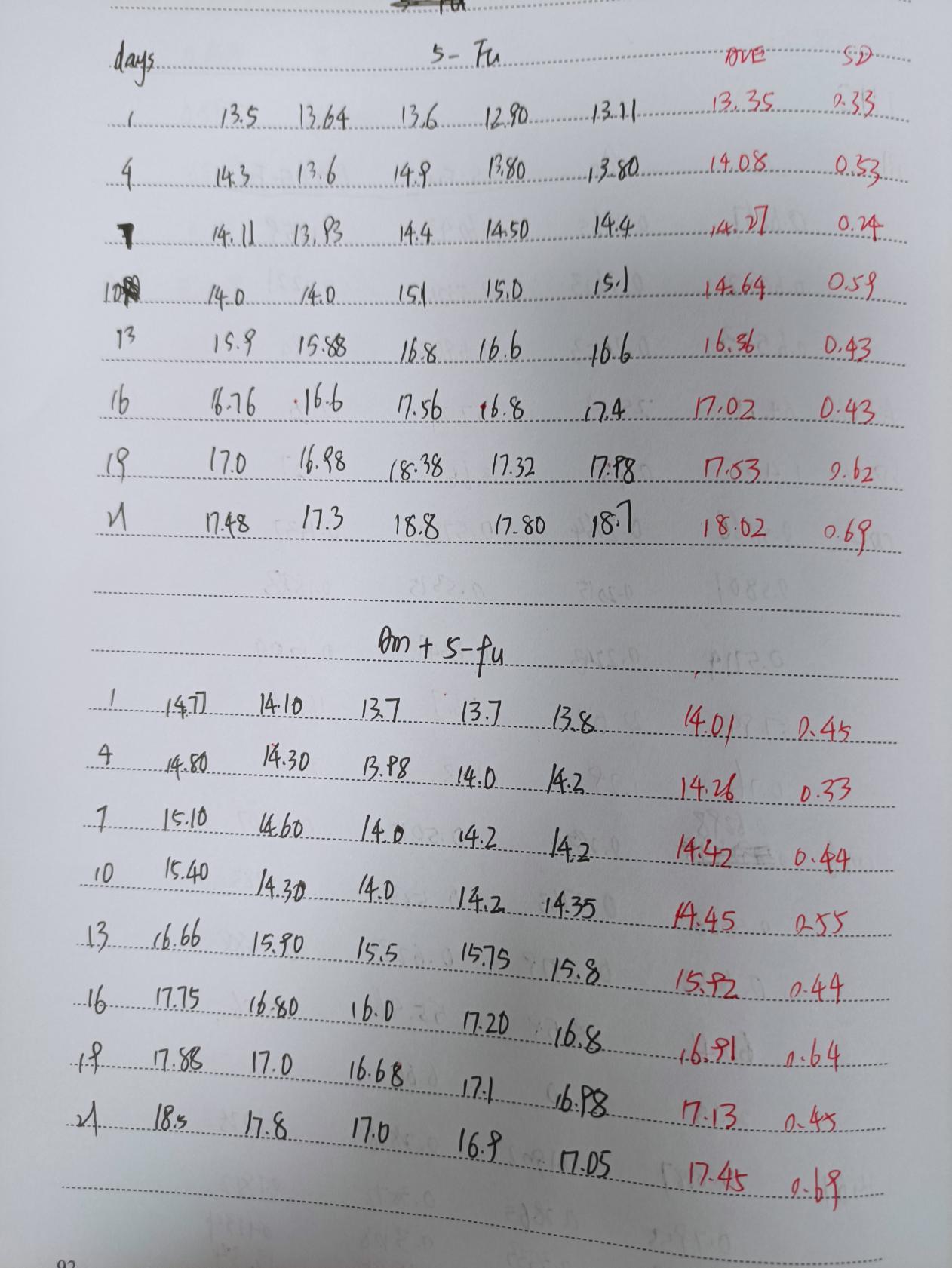


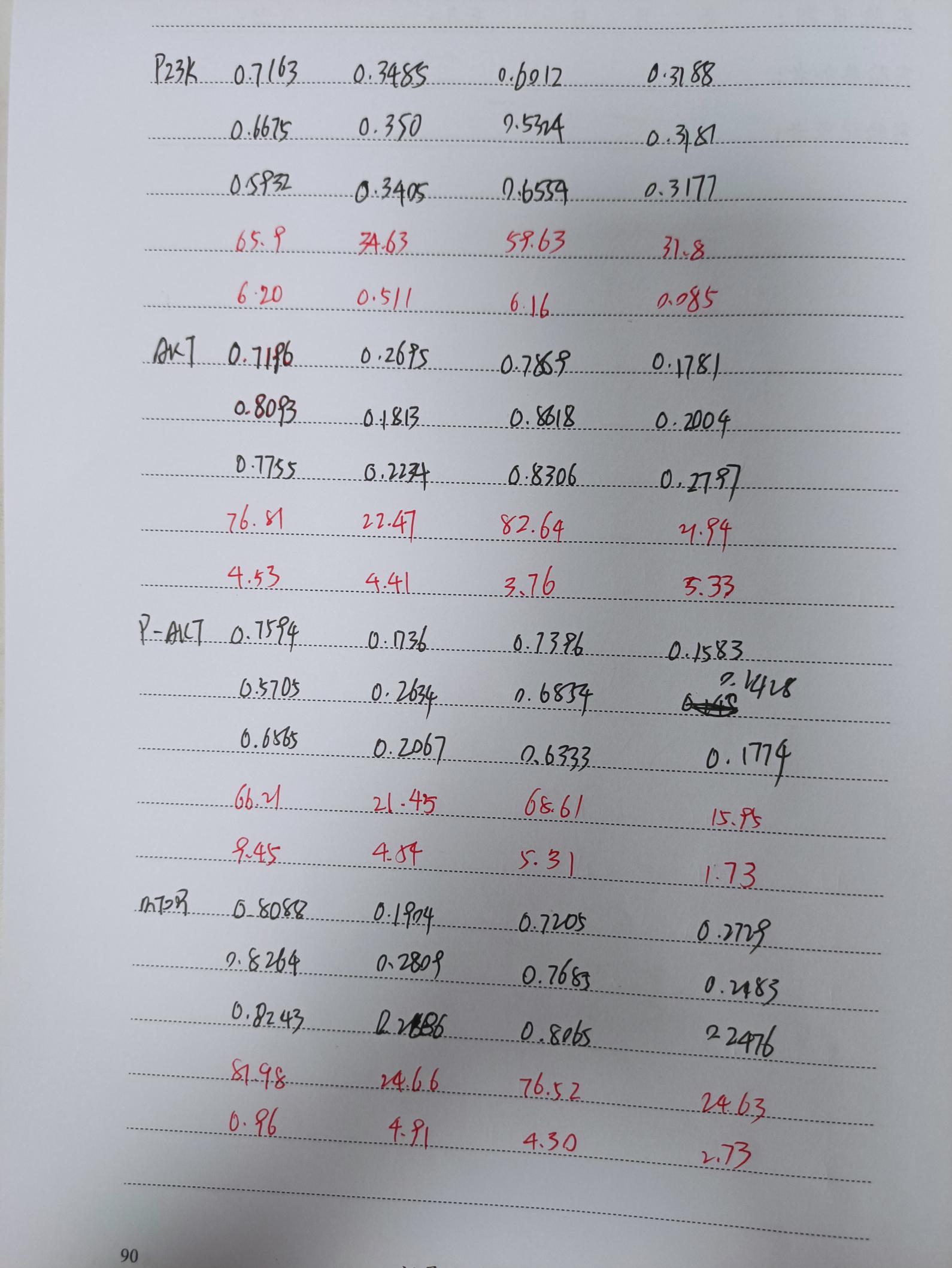

Supplement: Supplementary file 1 [file DataSheet1.ZIP › Original data of Aminoquinol (2)/Original data of Aminoquinol/In vivo/IHC.docx]

**Weight**


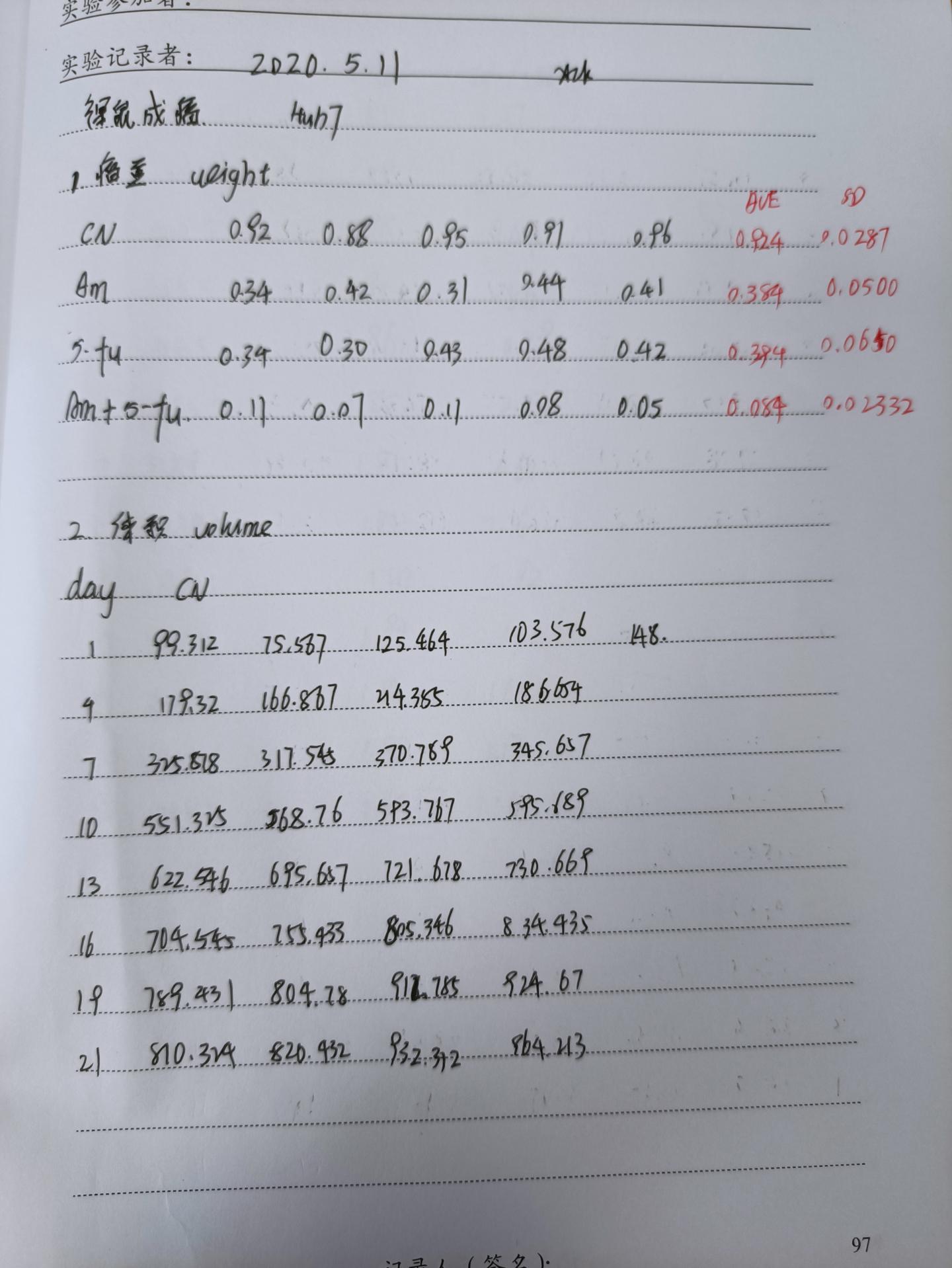


**volume**
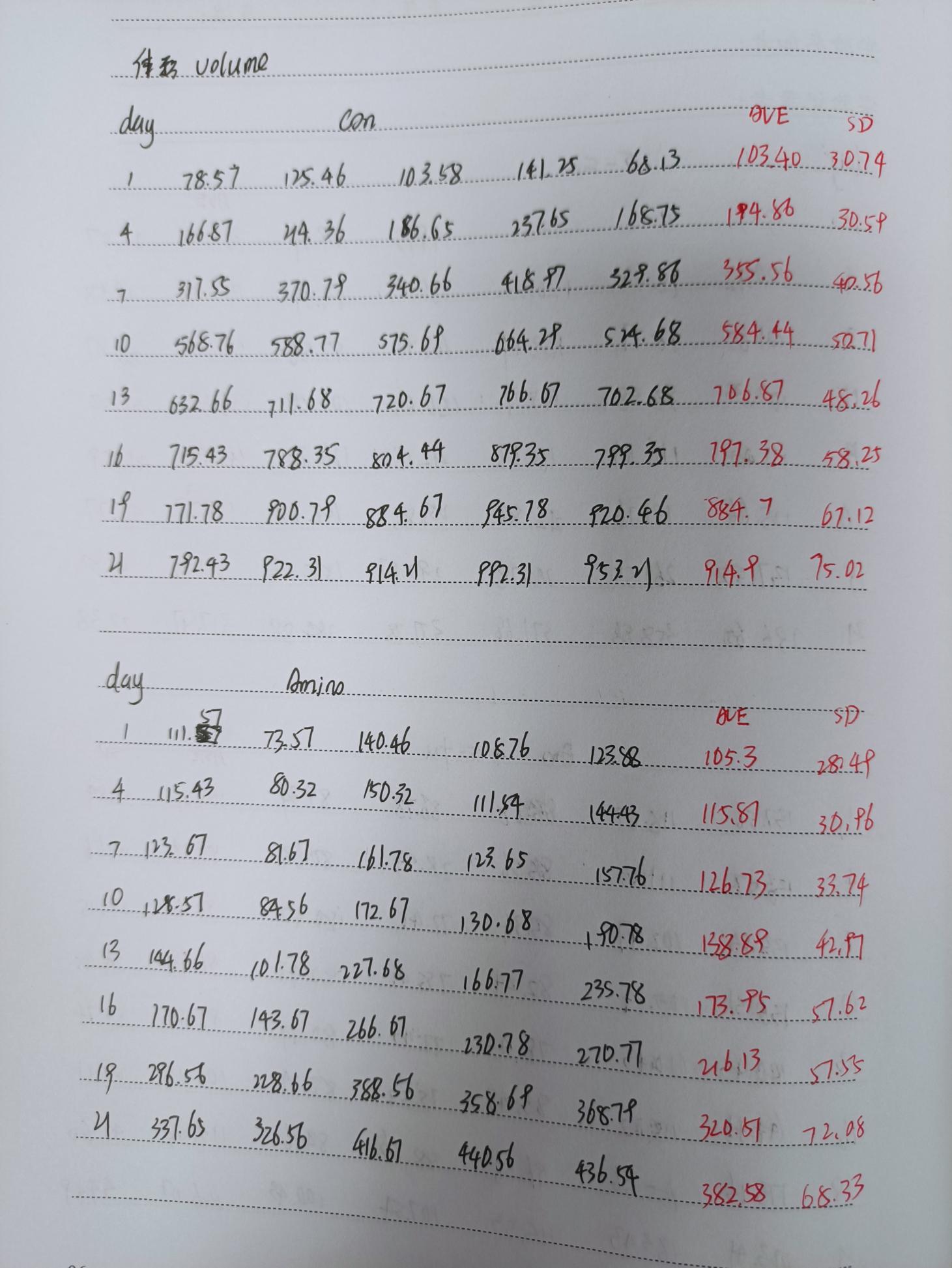

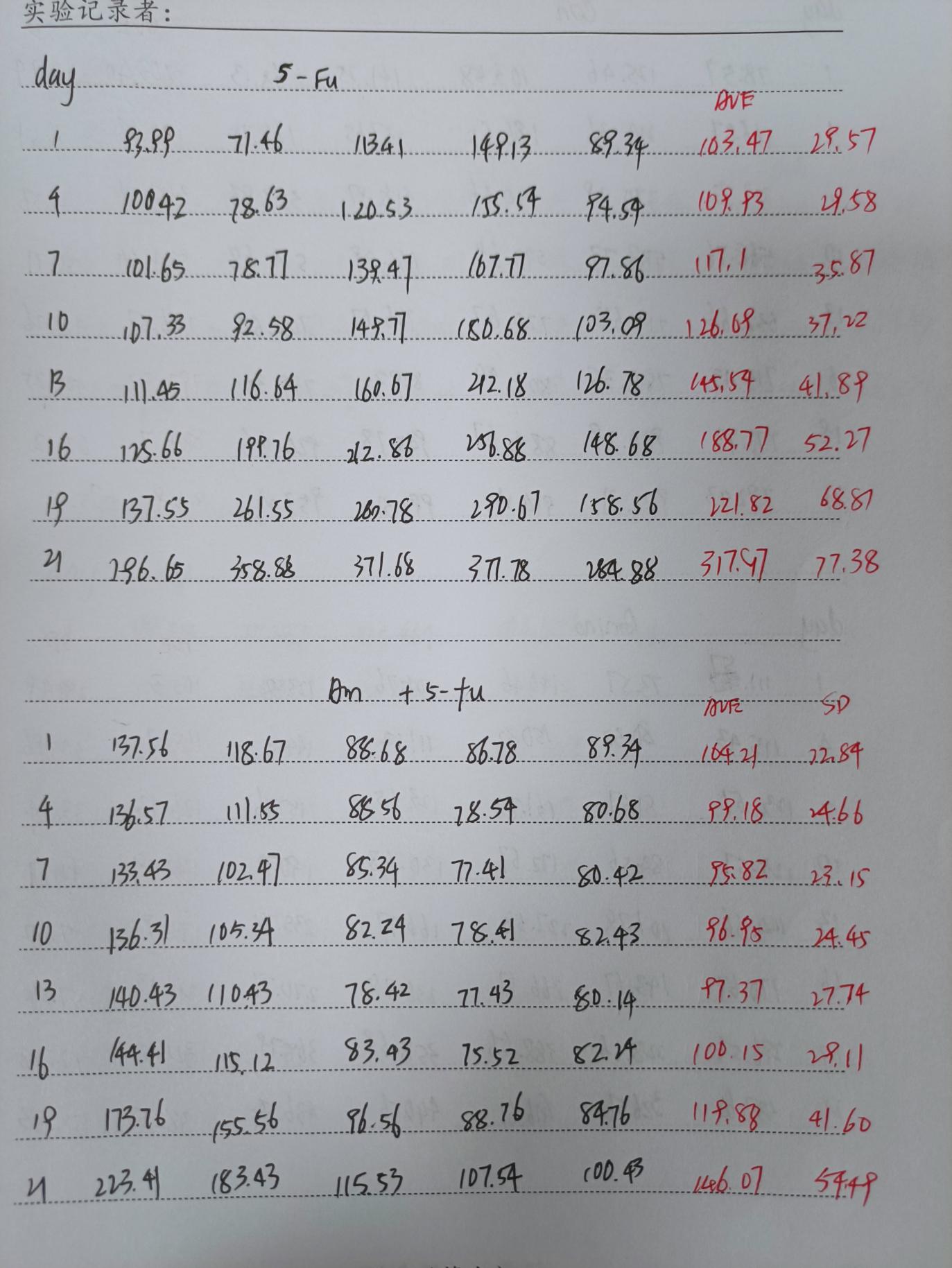


**Body weight**

**
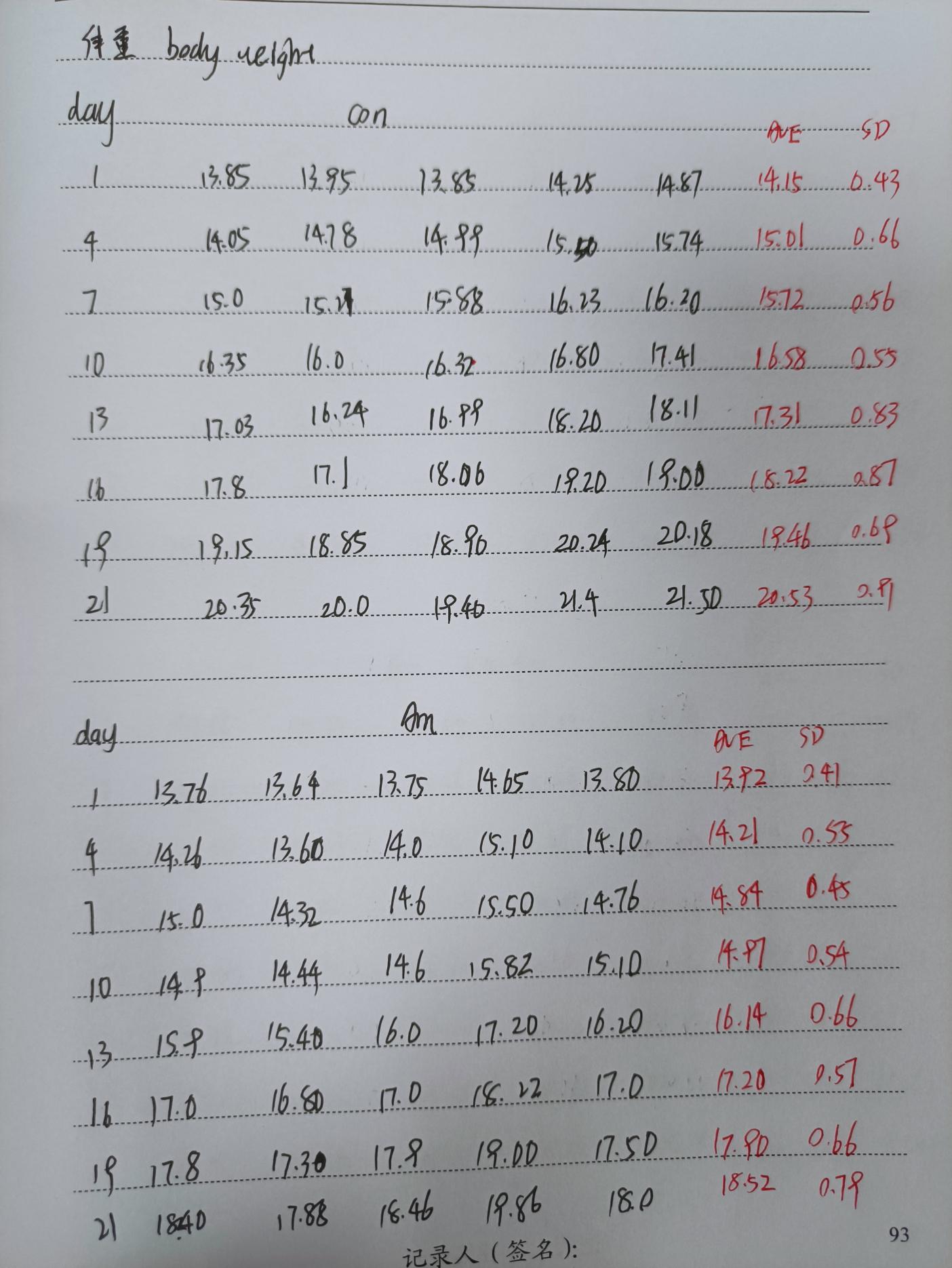

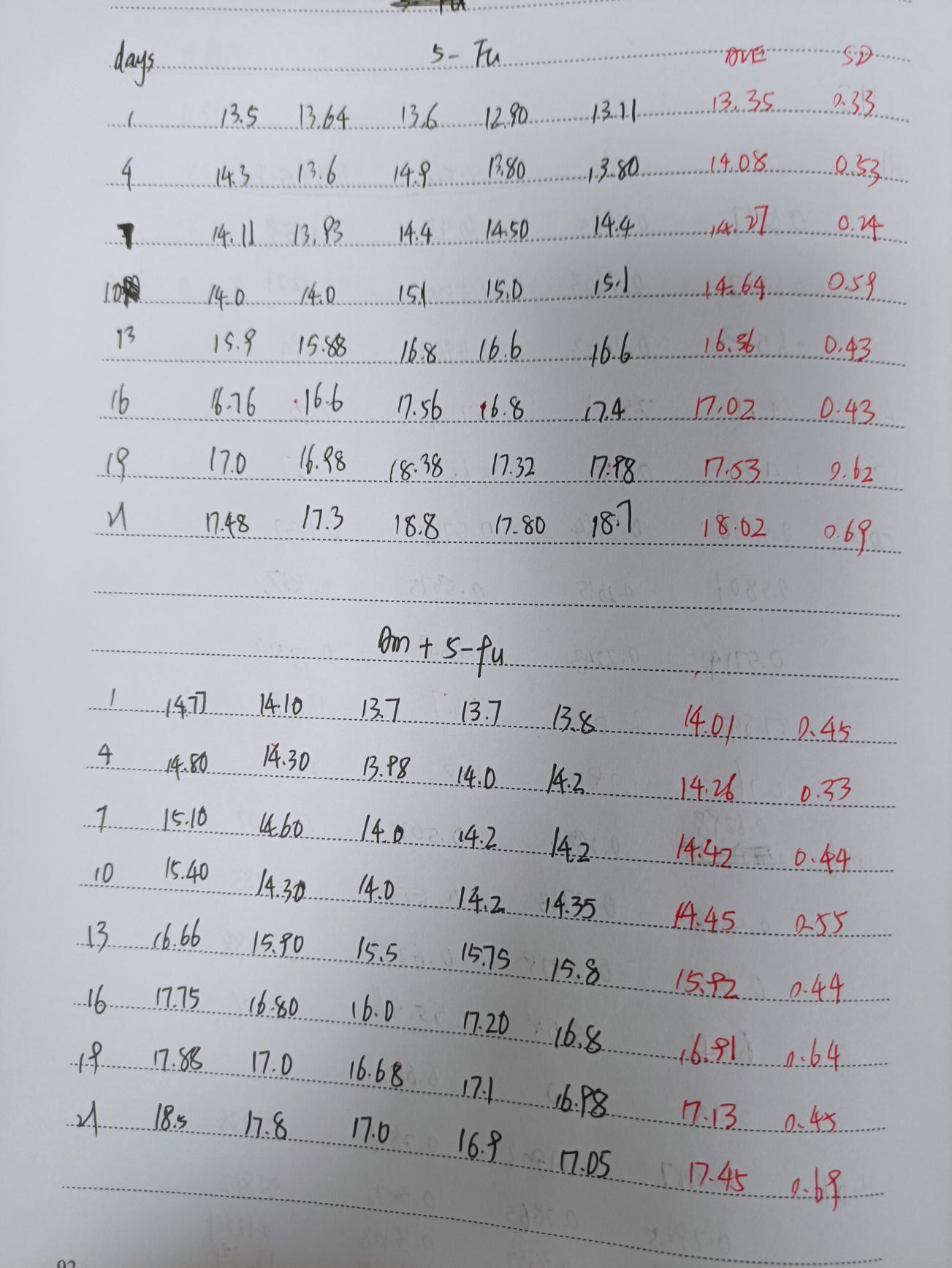
**

Supplement: Supplementary file 1 [file DataSheet1.ZIP › Original data of Aminoquinol (2)/Original data of Aminoquinol/In vivo/Tumor.docx]

**CDK**

**Huh7**

**
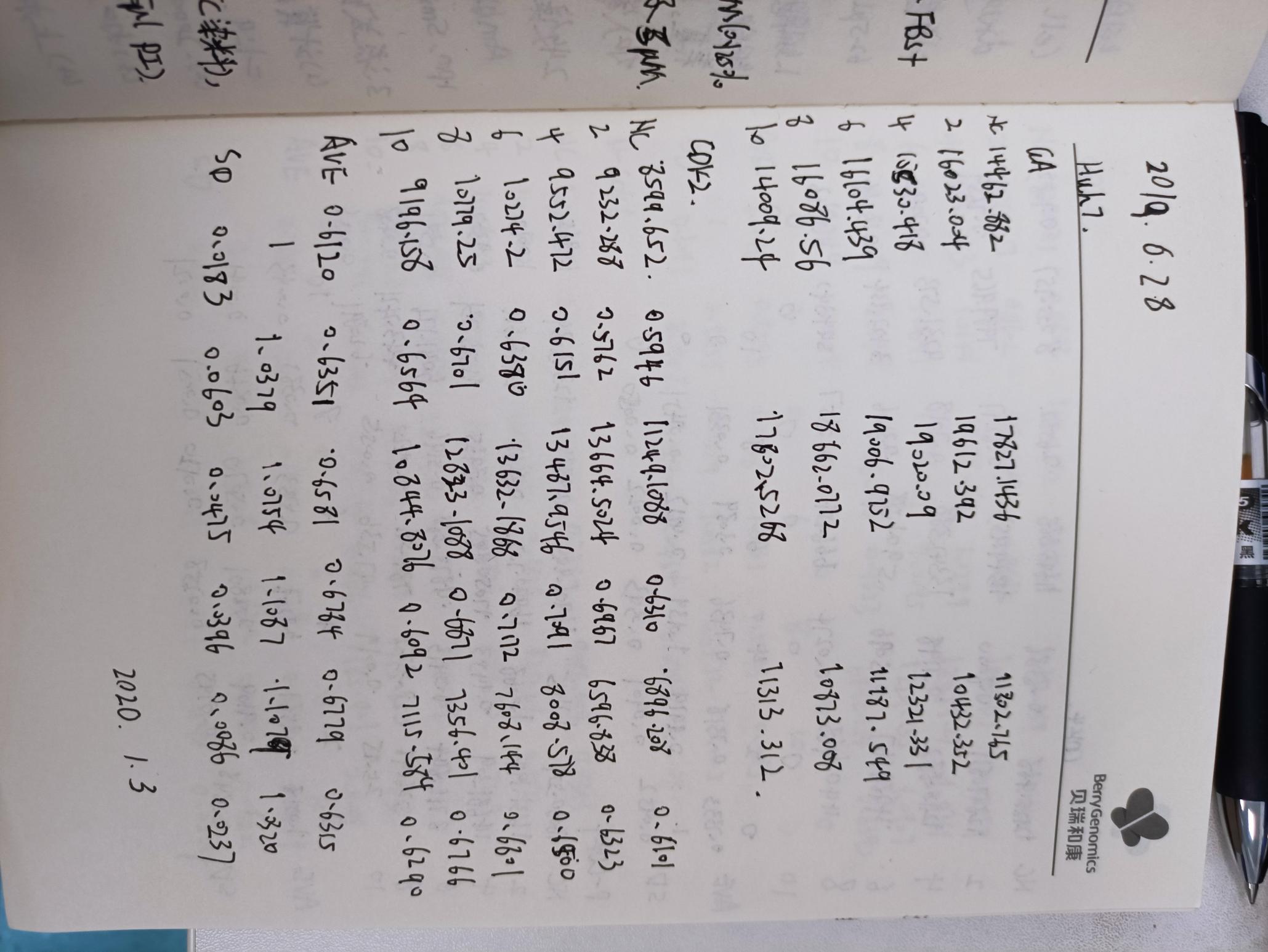
**


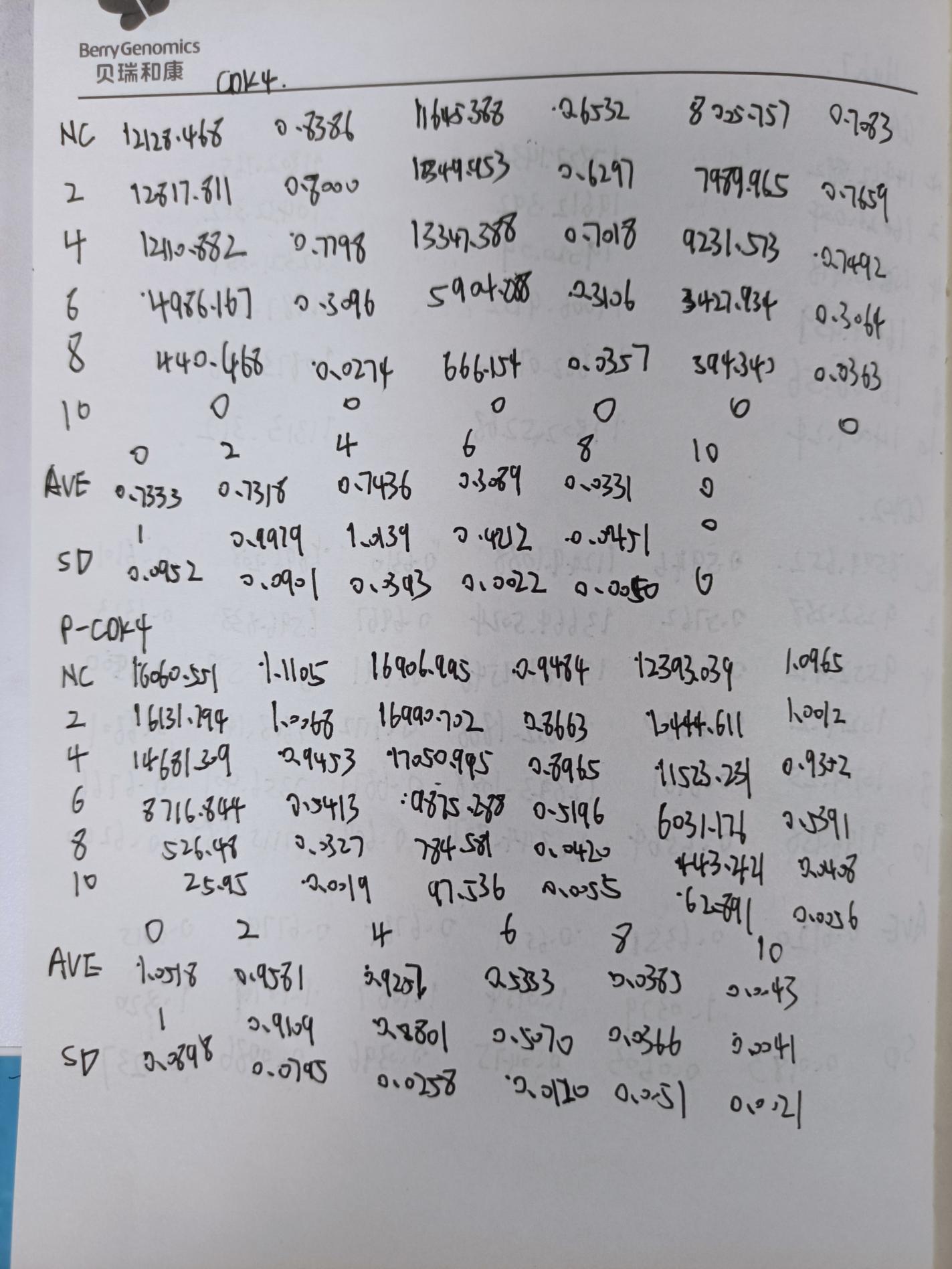

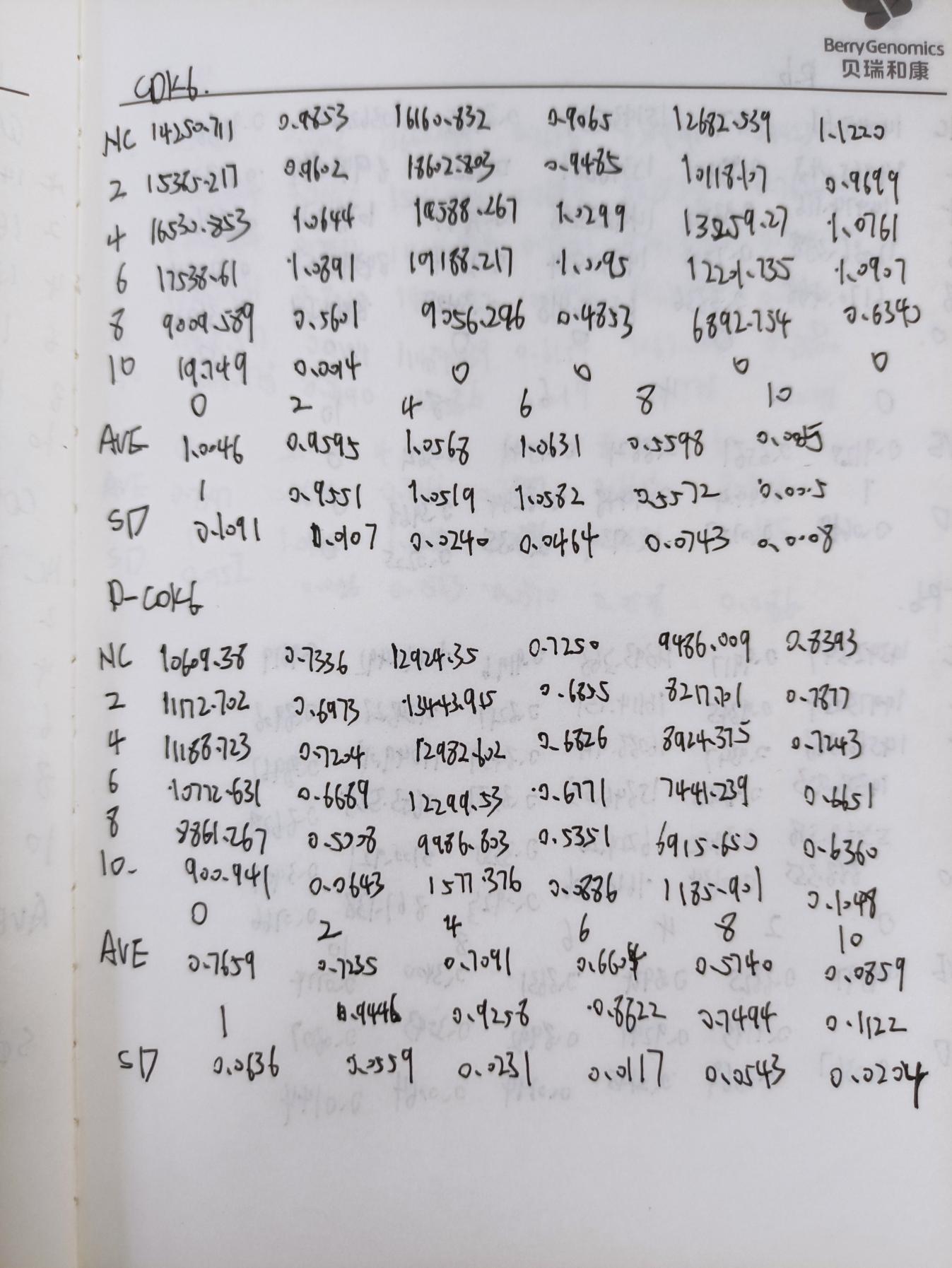

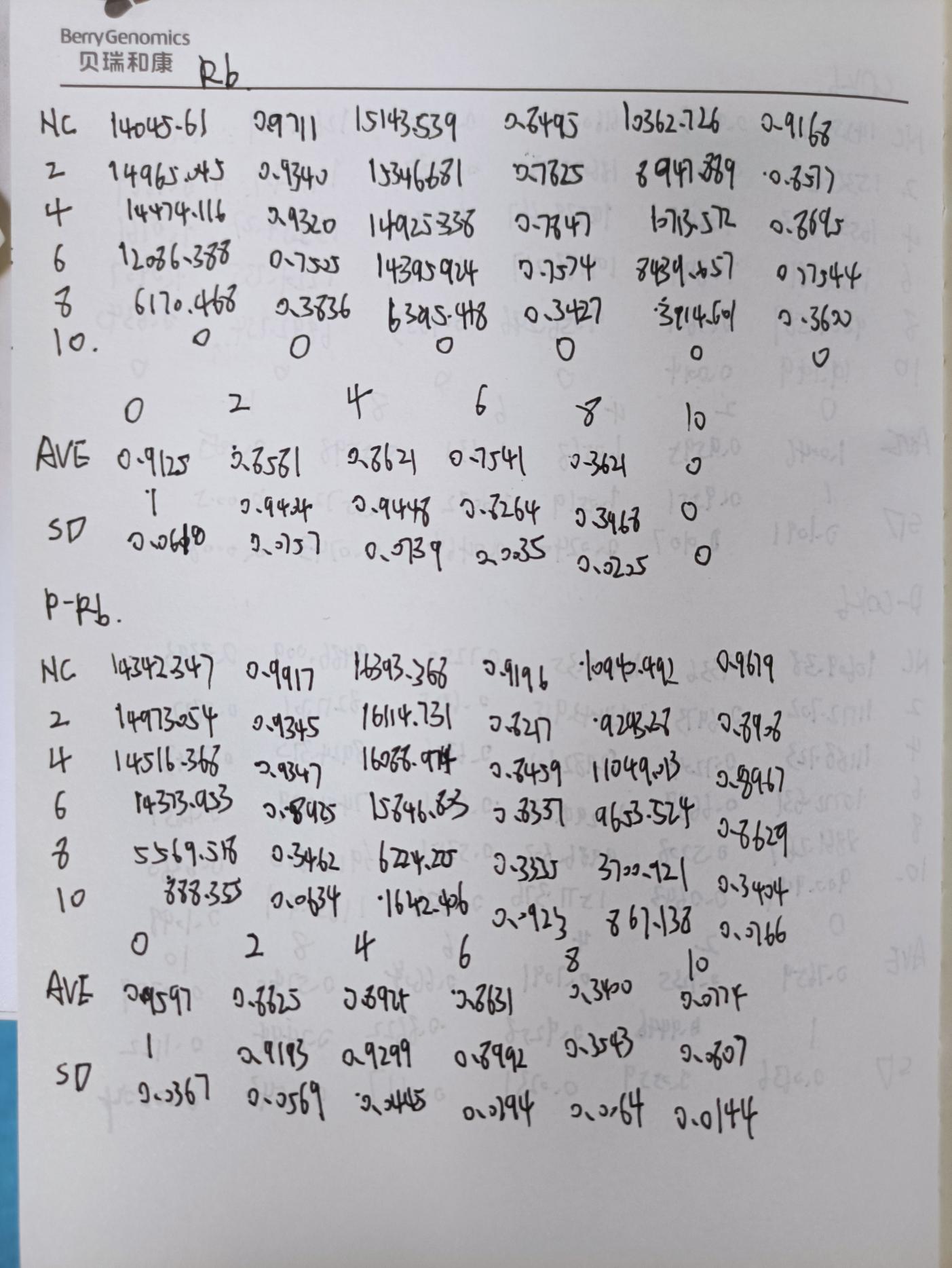


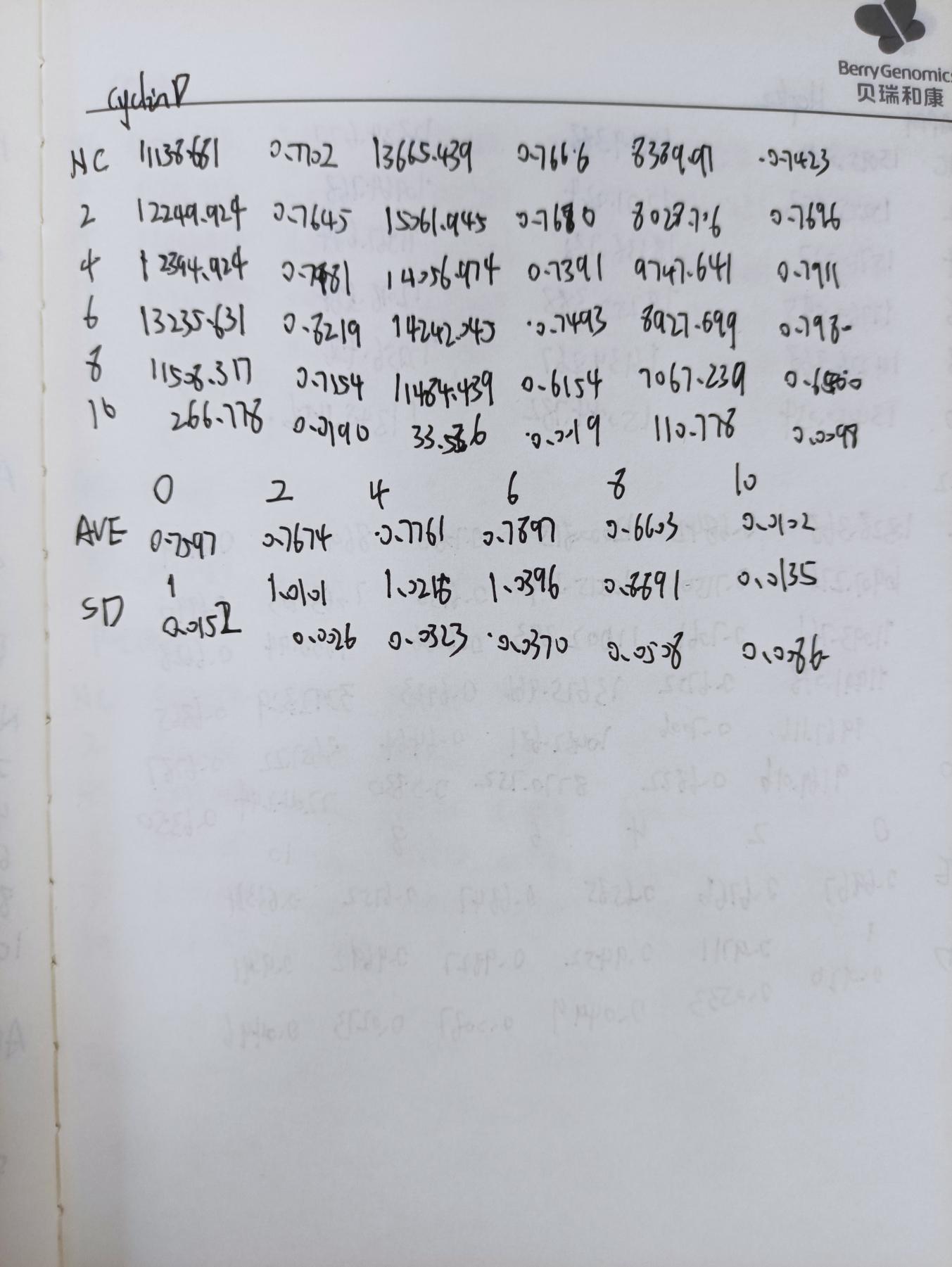


**HepG2**


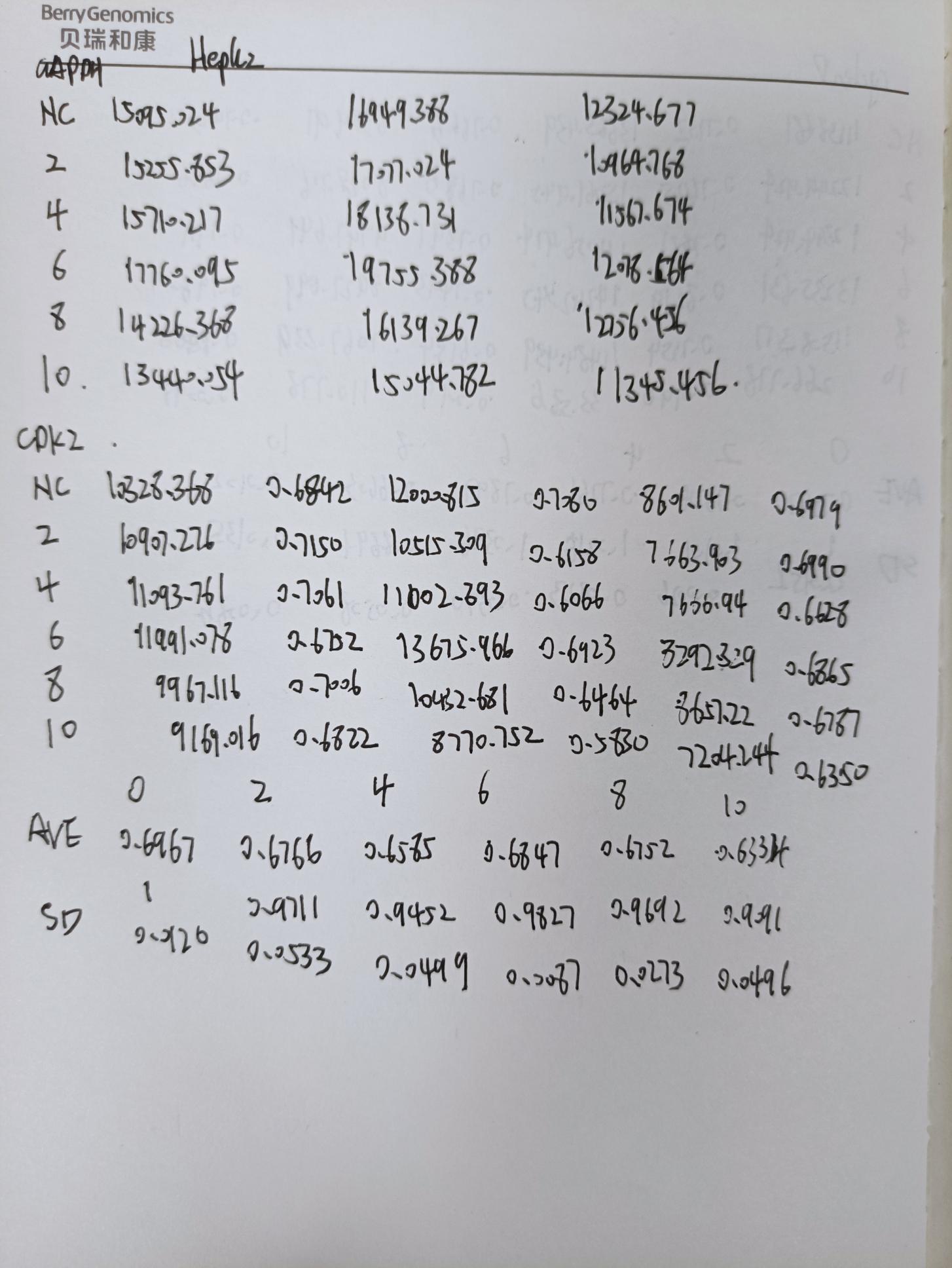

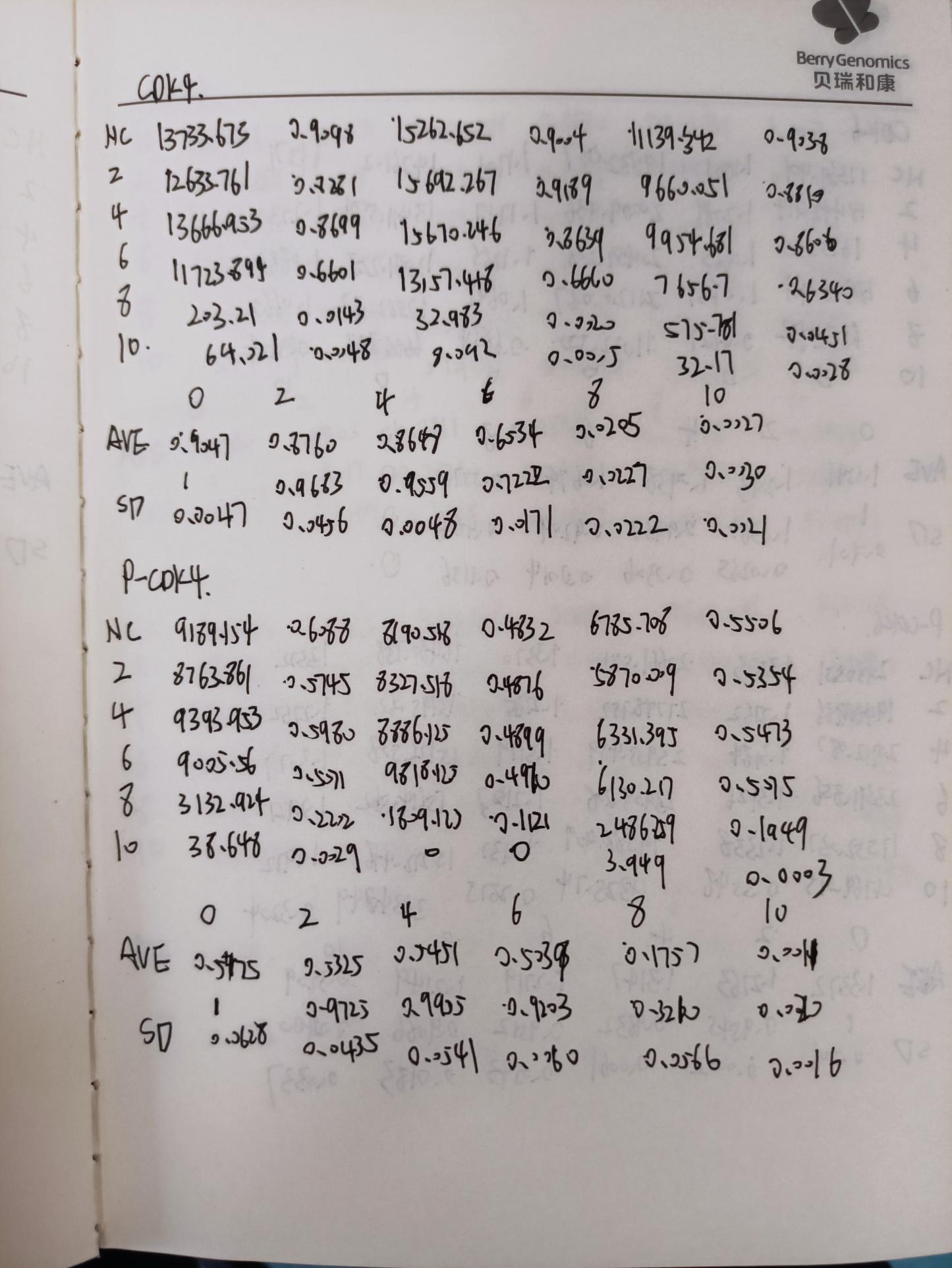


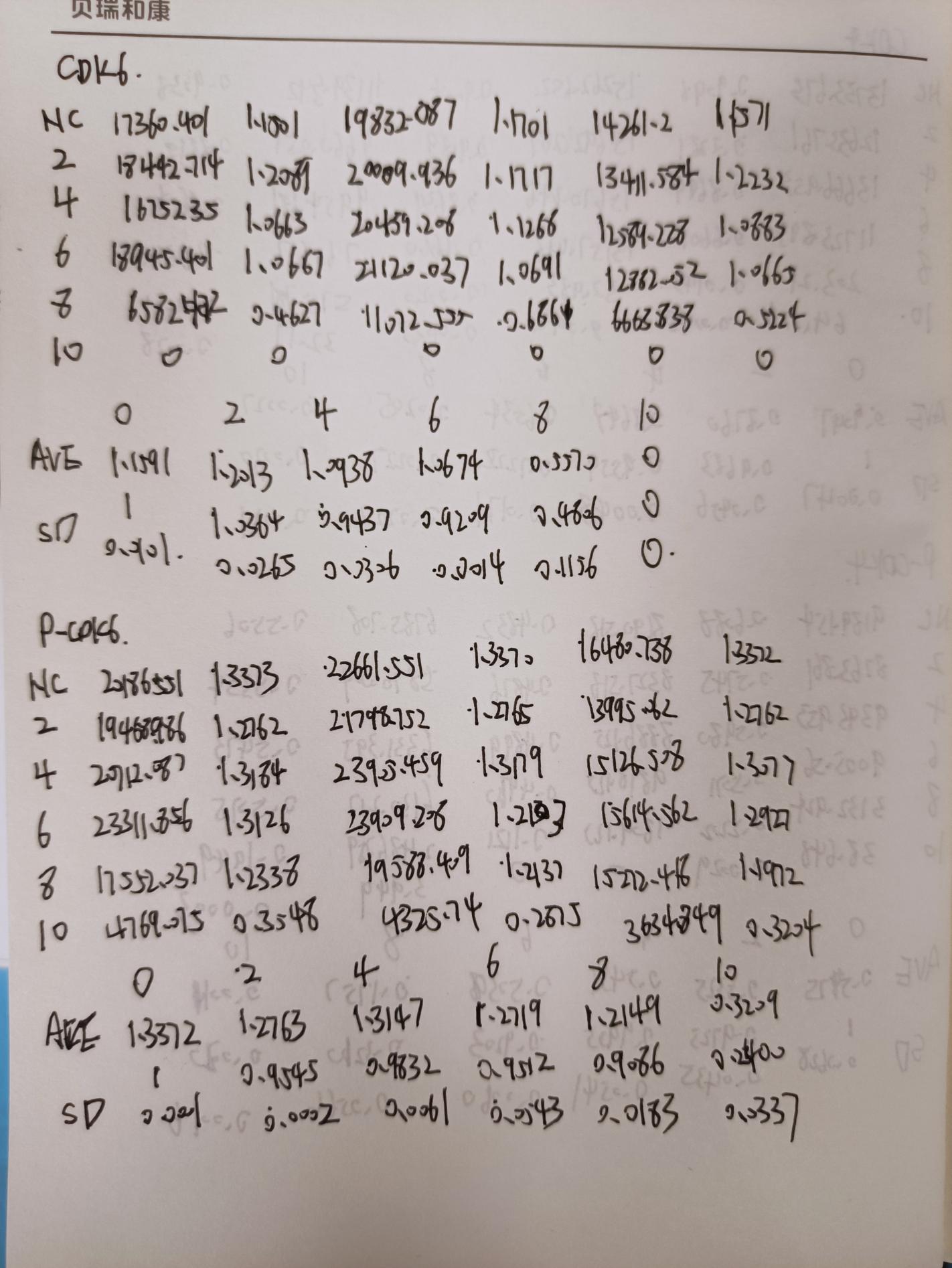


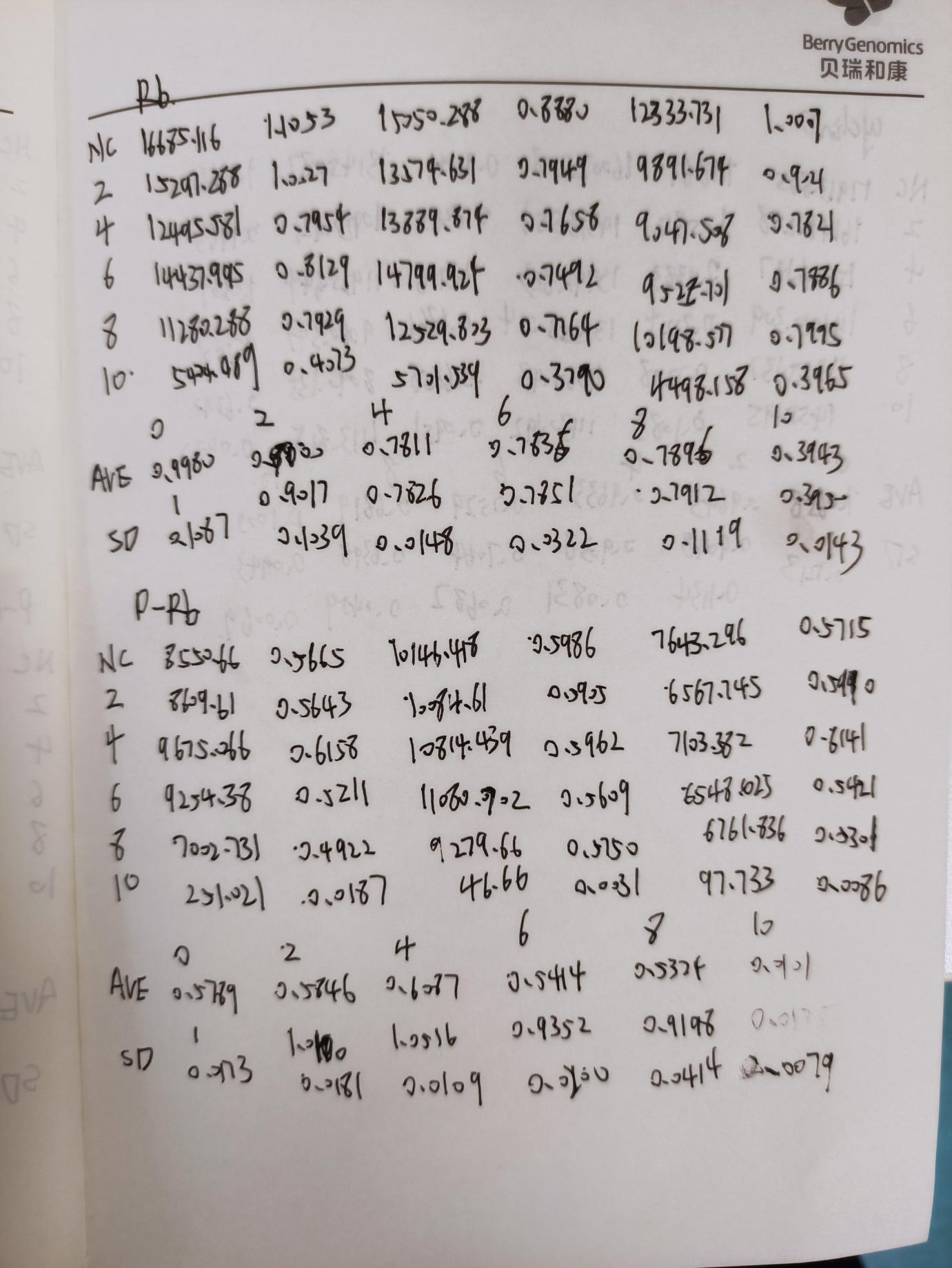


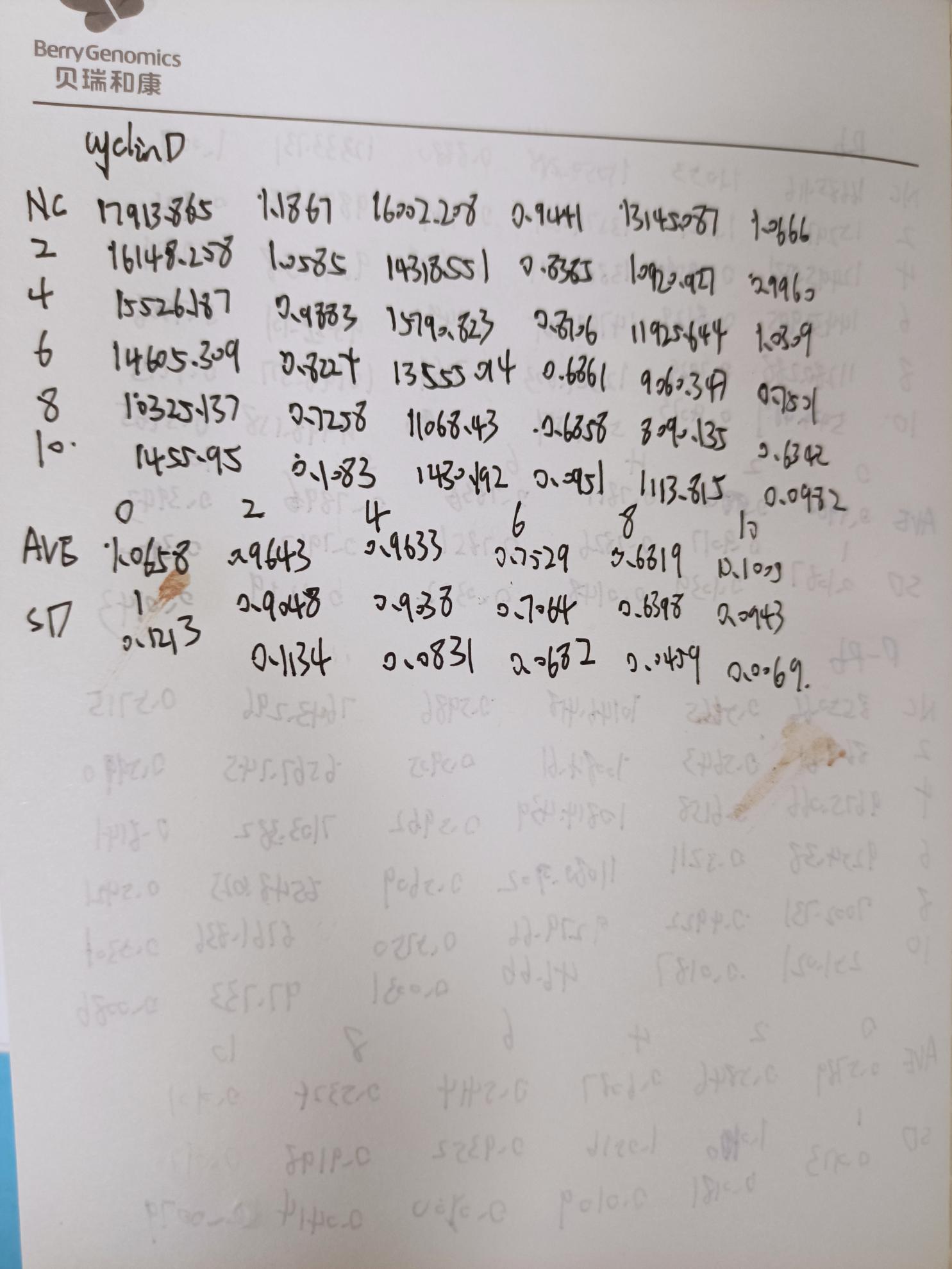

Supplement: Supplementary file 1 [file DataSheet1.ZIP › Original data of Aminoquinol (2)/Original data of Aminoquinol/Western Blot/CDK.docx]

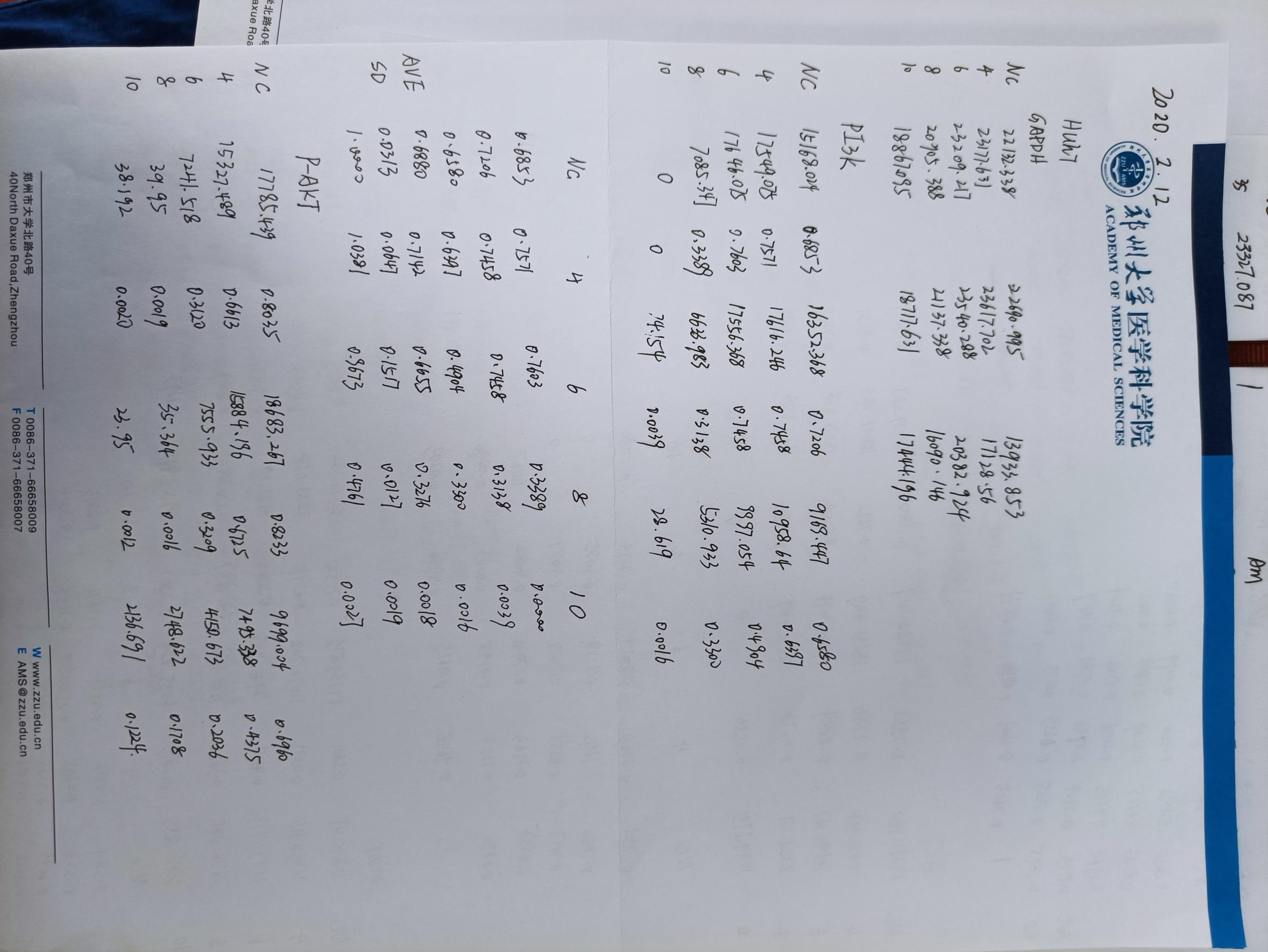

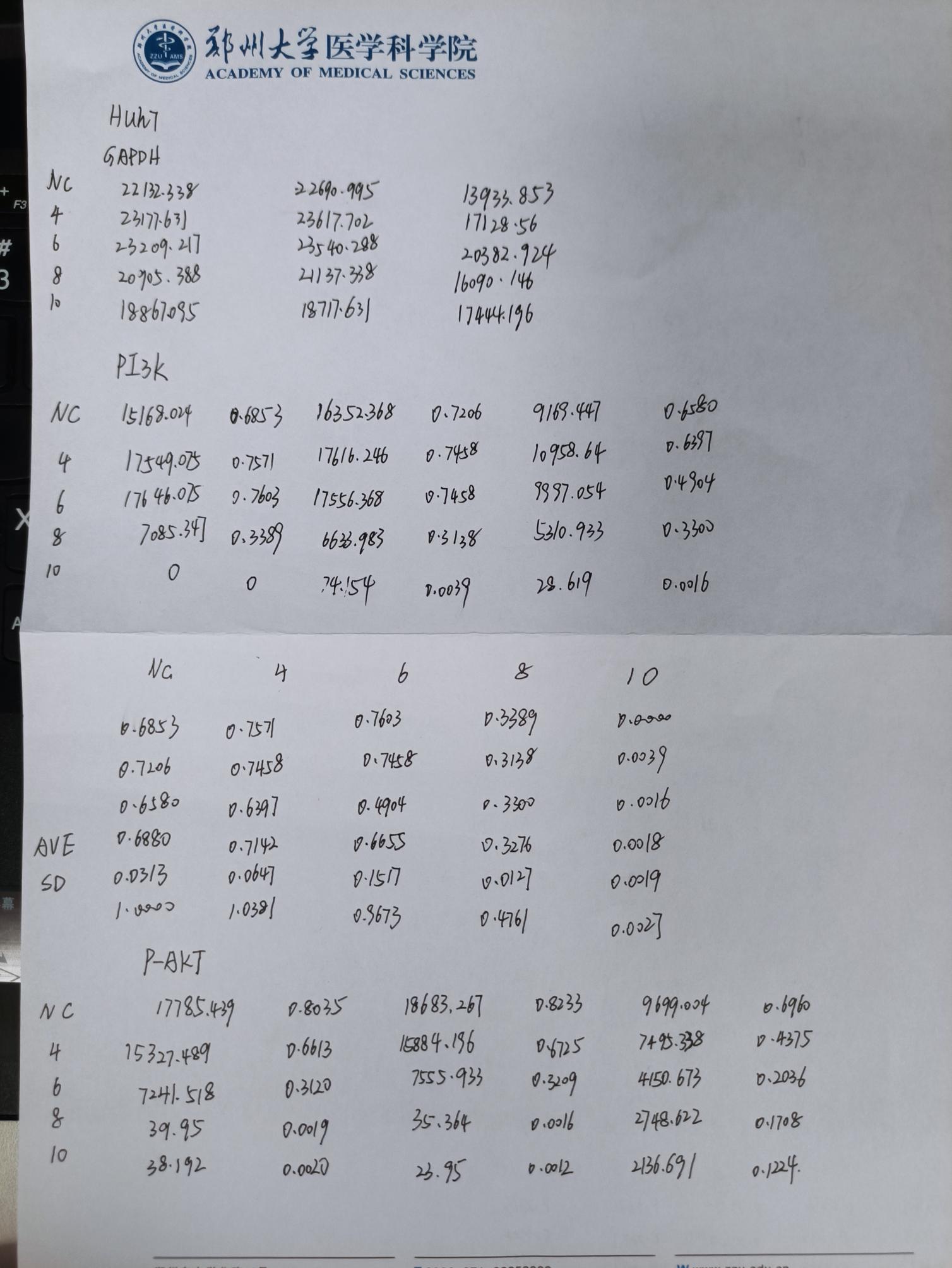


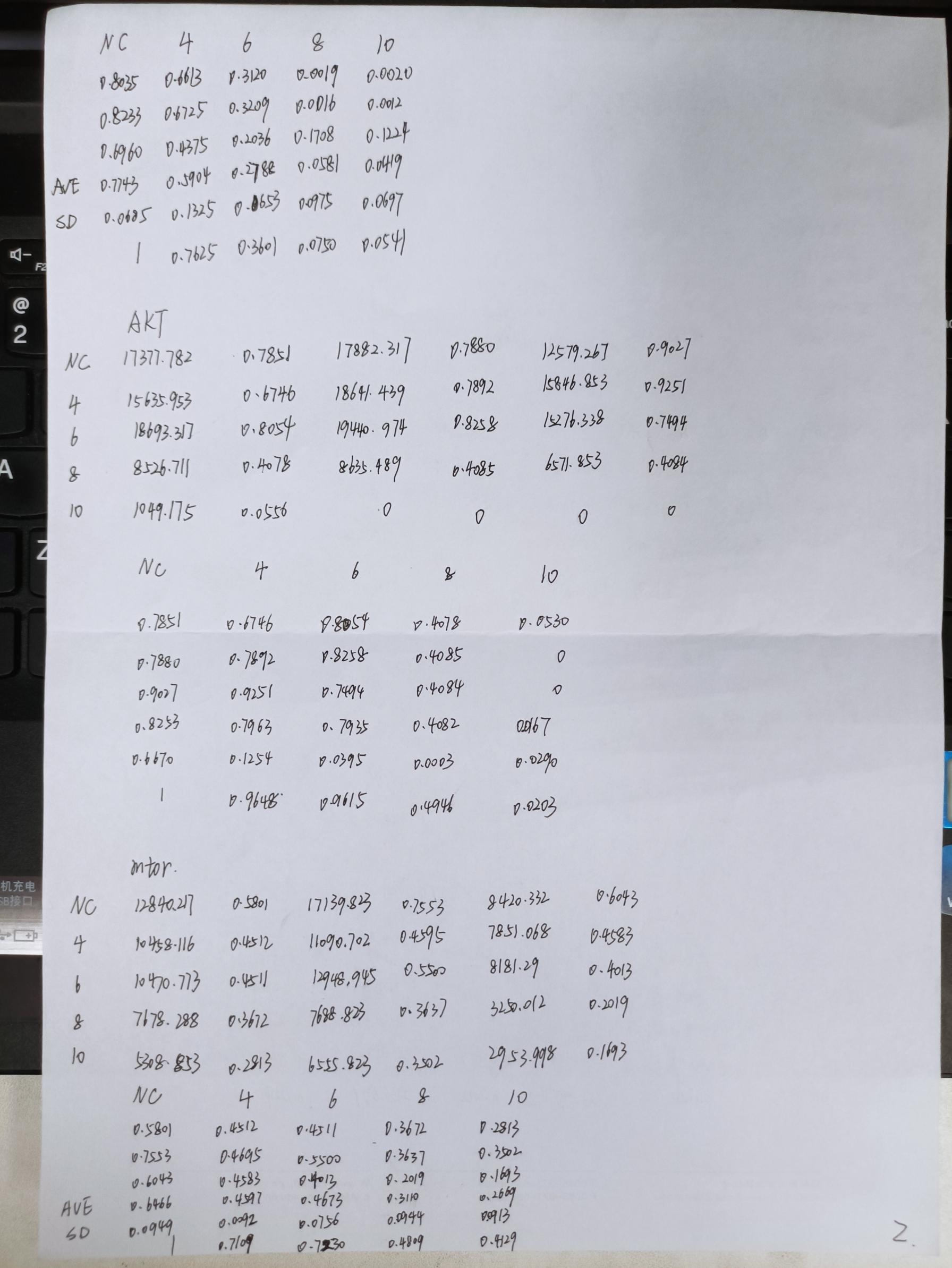

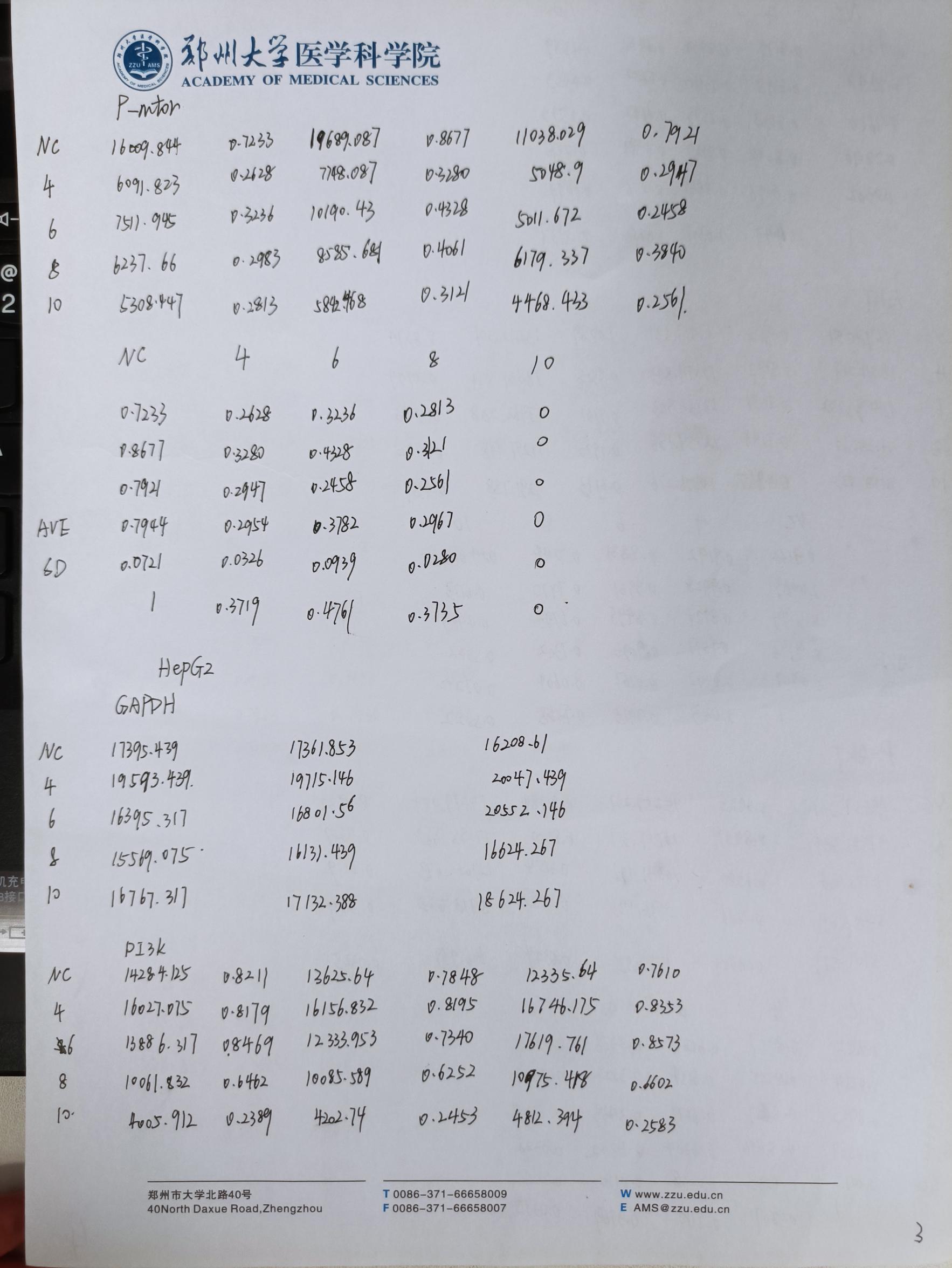

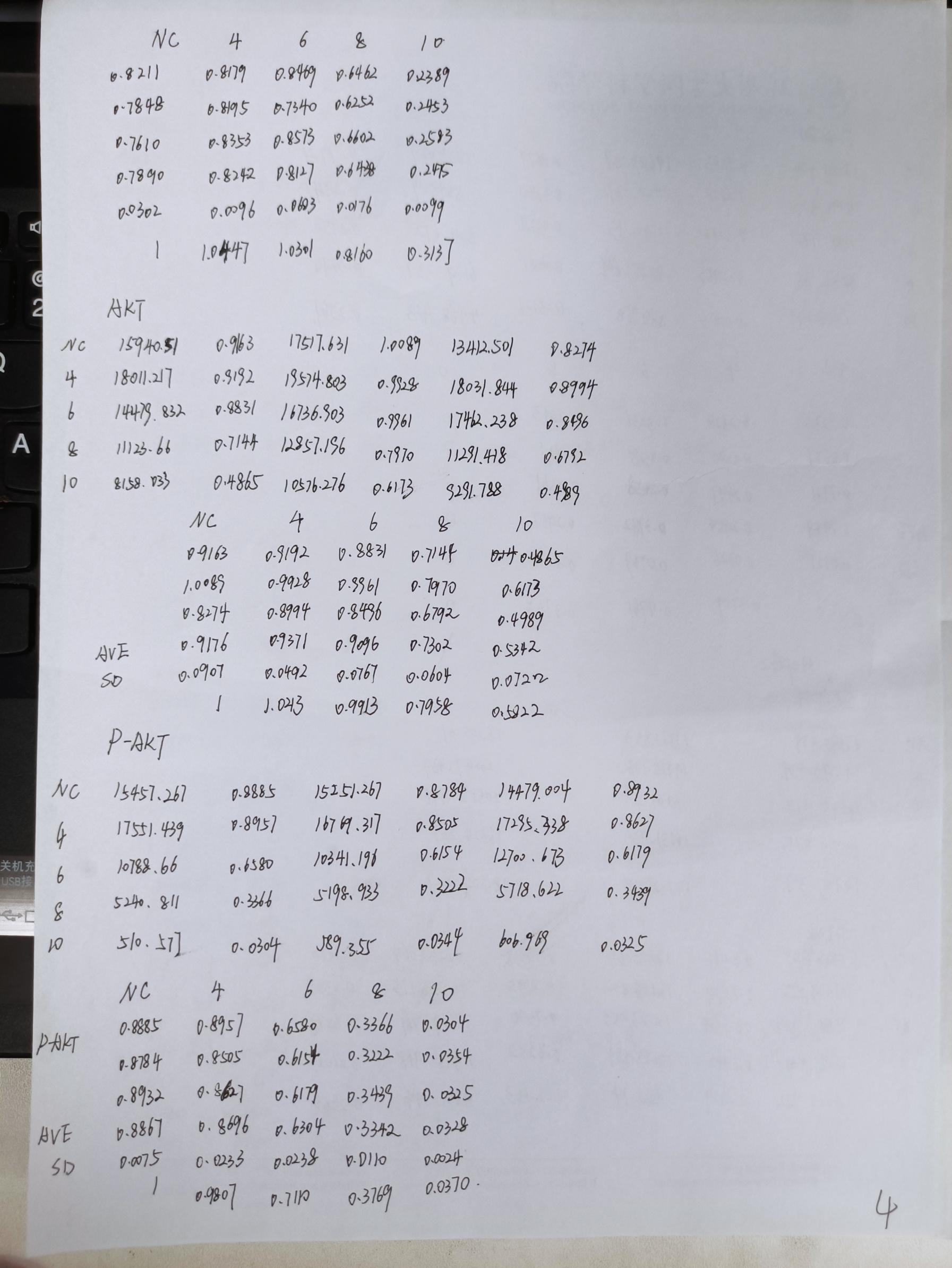

Supplement: Supplementary file 1 [file DataSheet1.ZIP › Original data of Aminoquinol (2)/Original data of Aminoquinol/Western Blot/PI3K-AKT-MTOR.docx]

**TPP**

**CDK4**

**
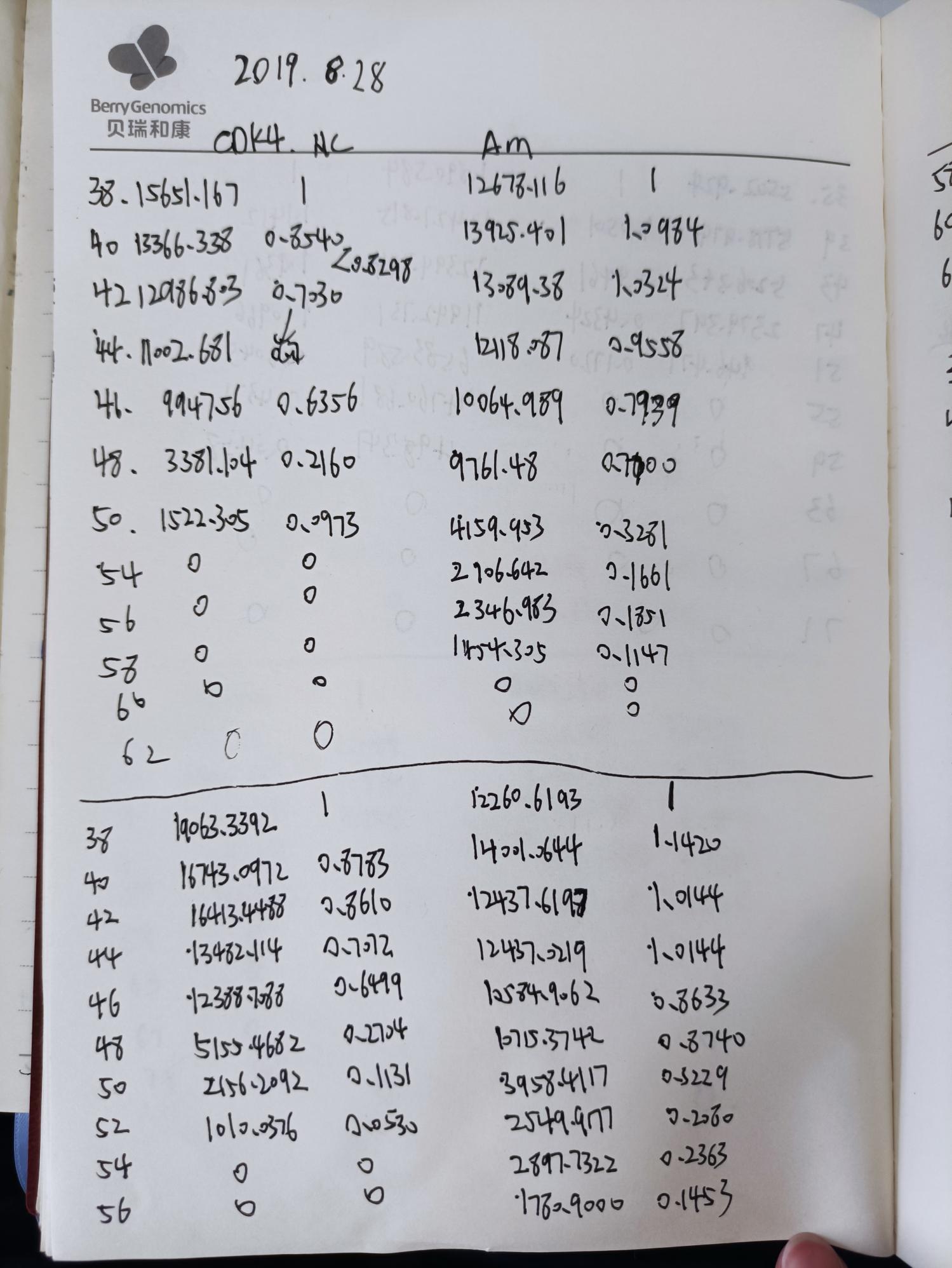
**

**
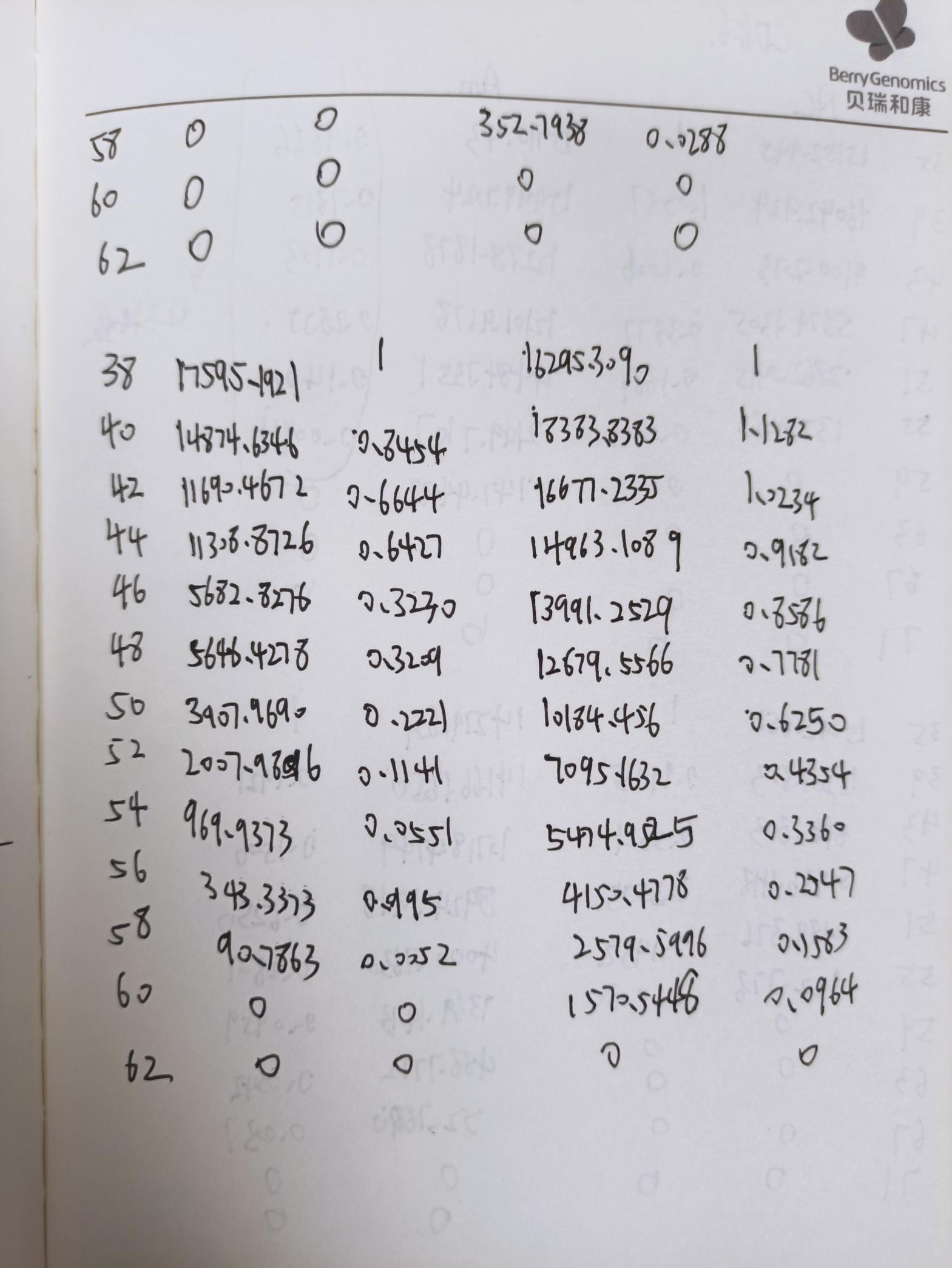
**

**CDK6**

**
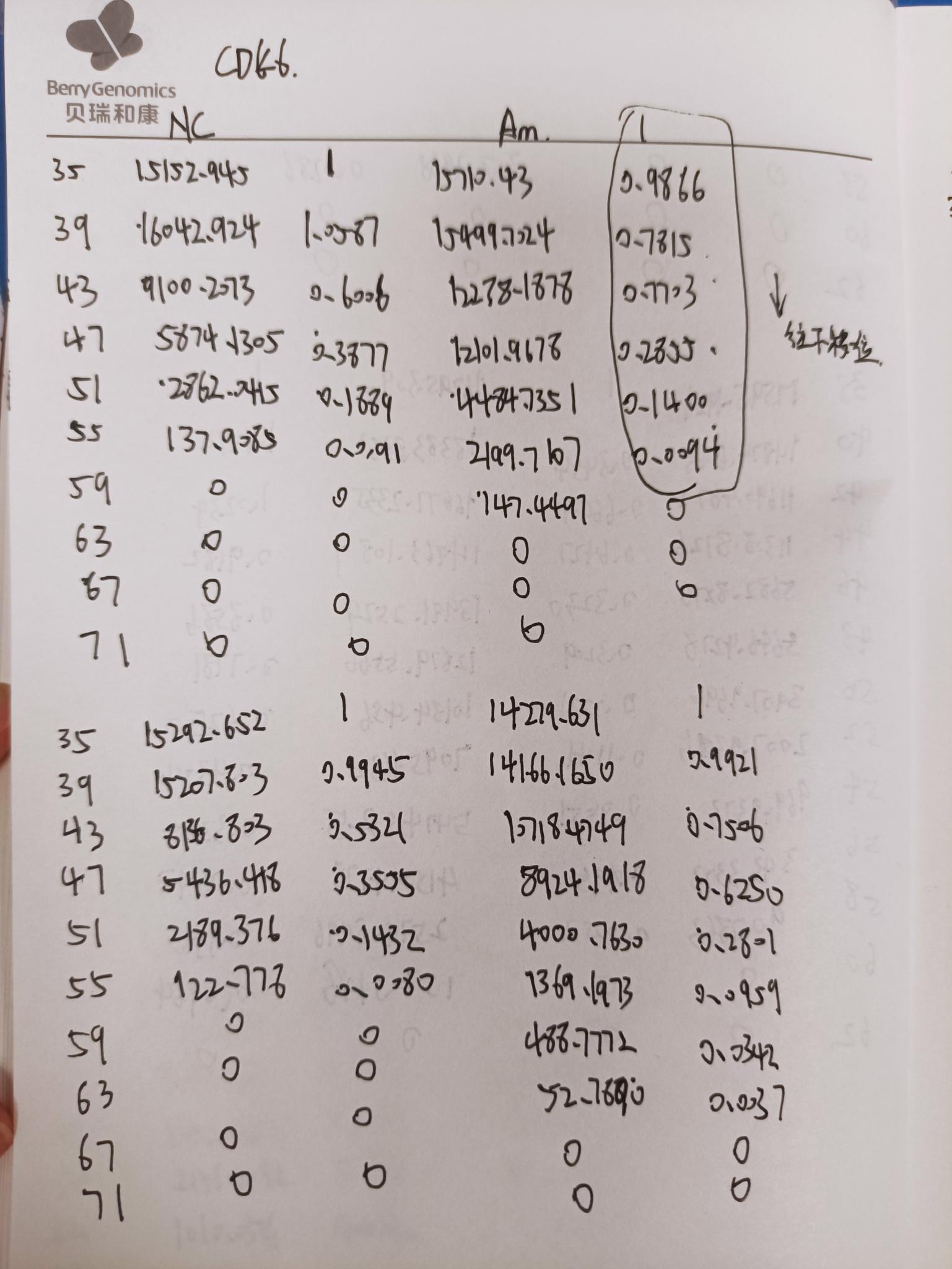

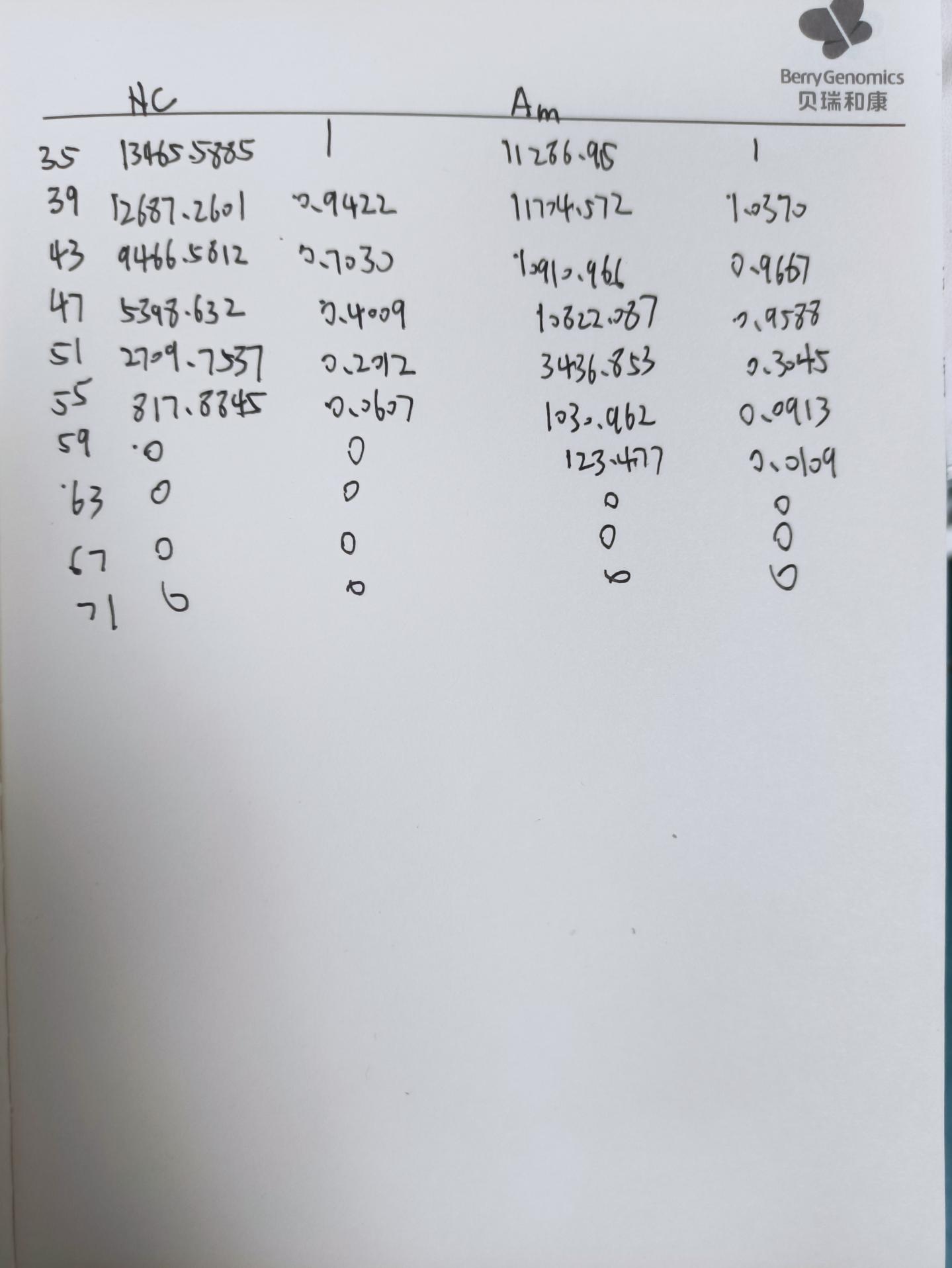
**

**PI3K**

**
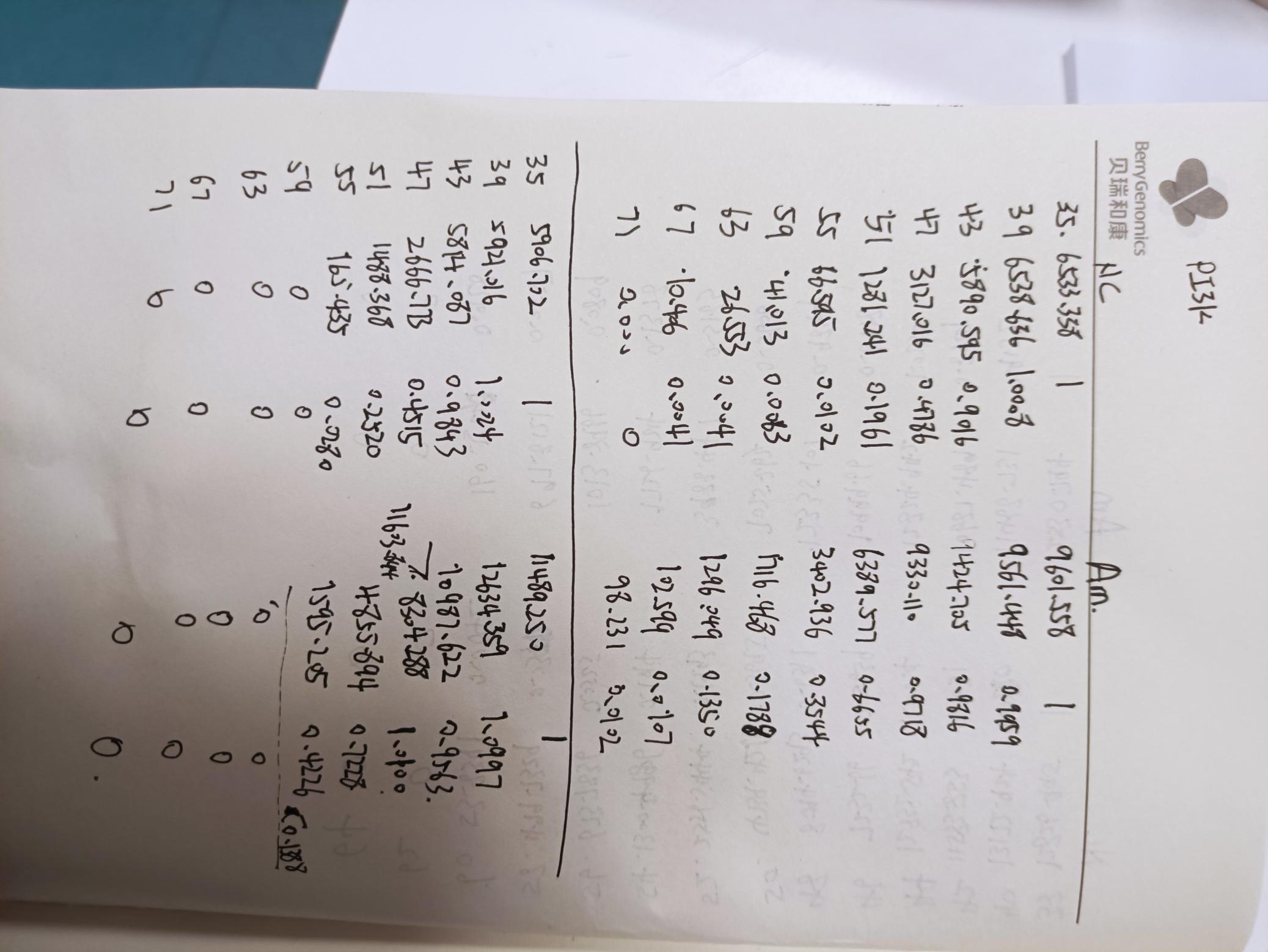

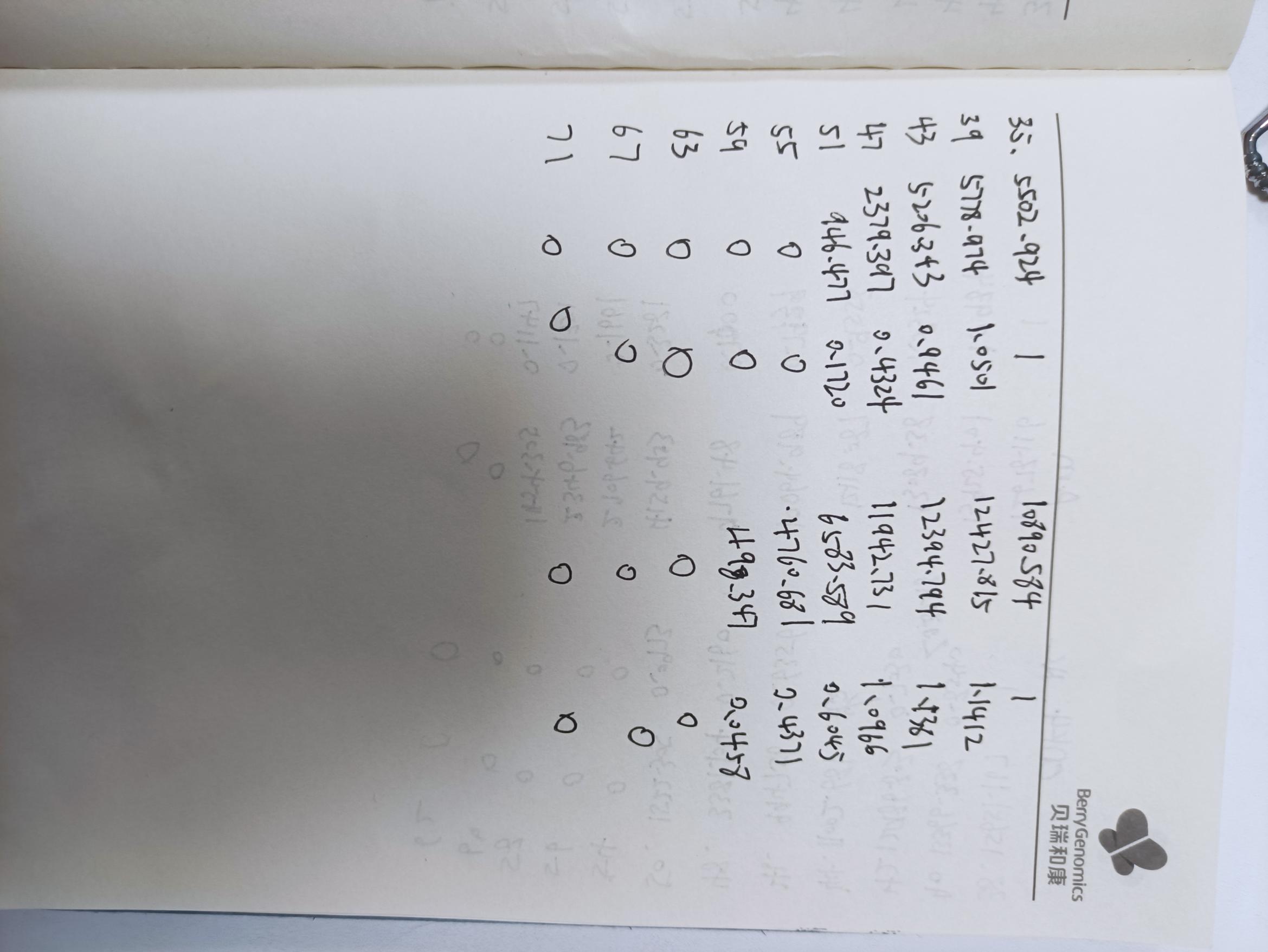
**

**AKT**

**
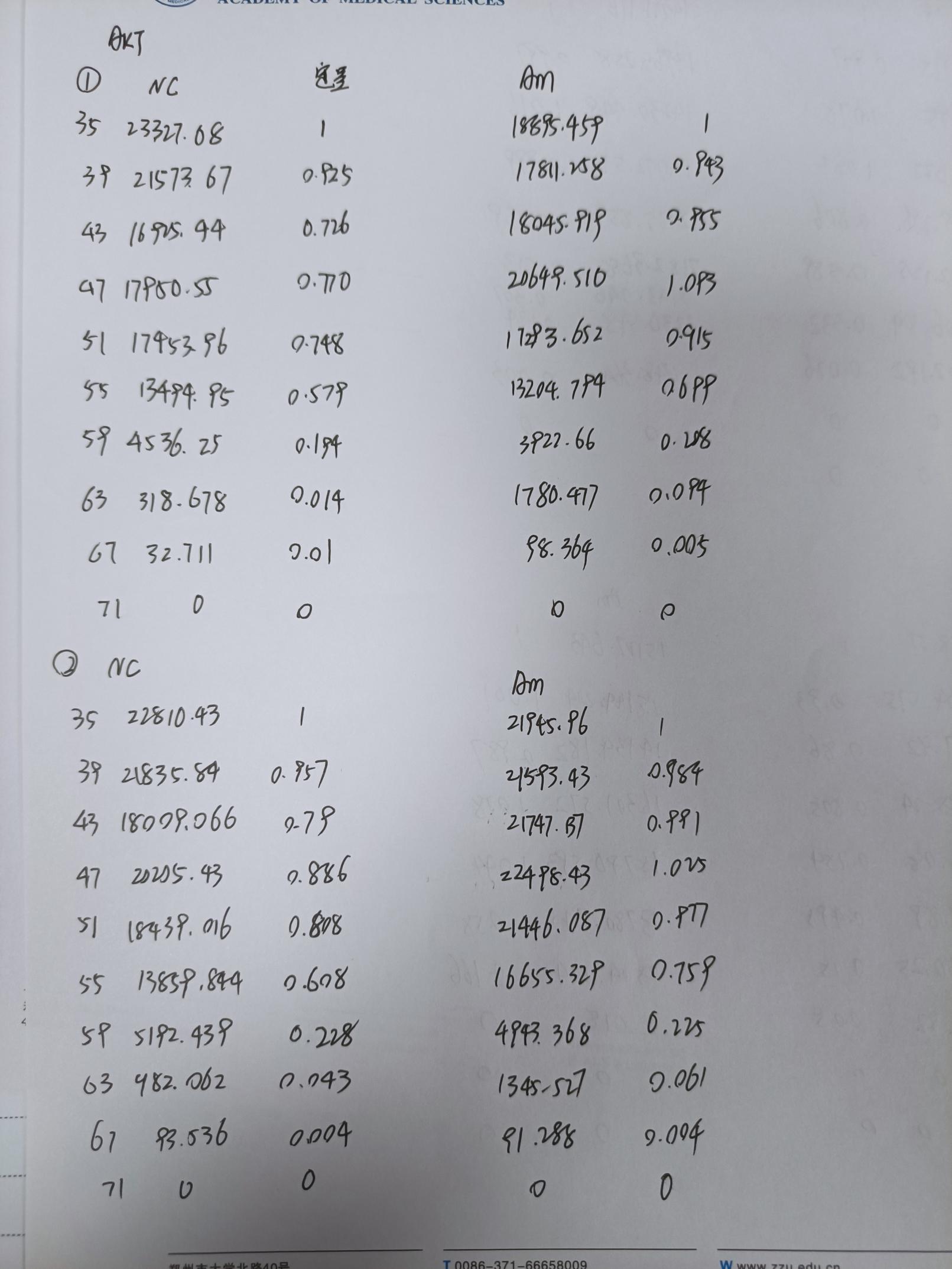

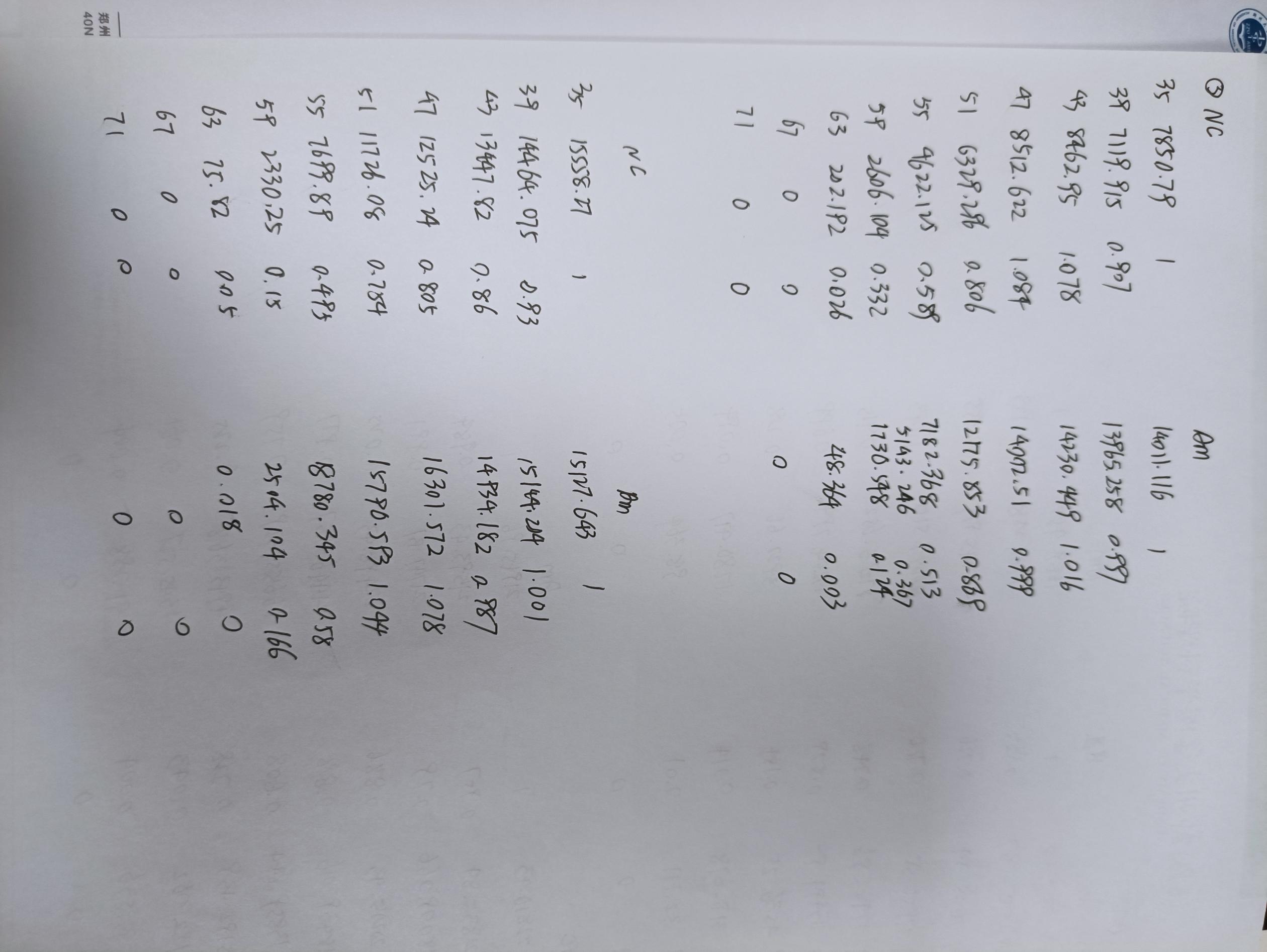
**

1. **AKT**

**
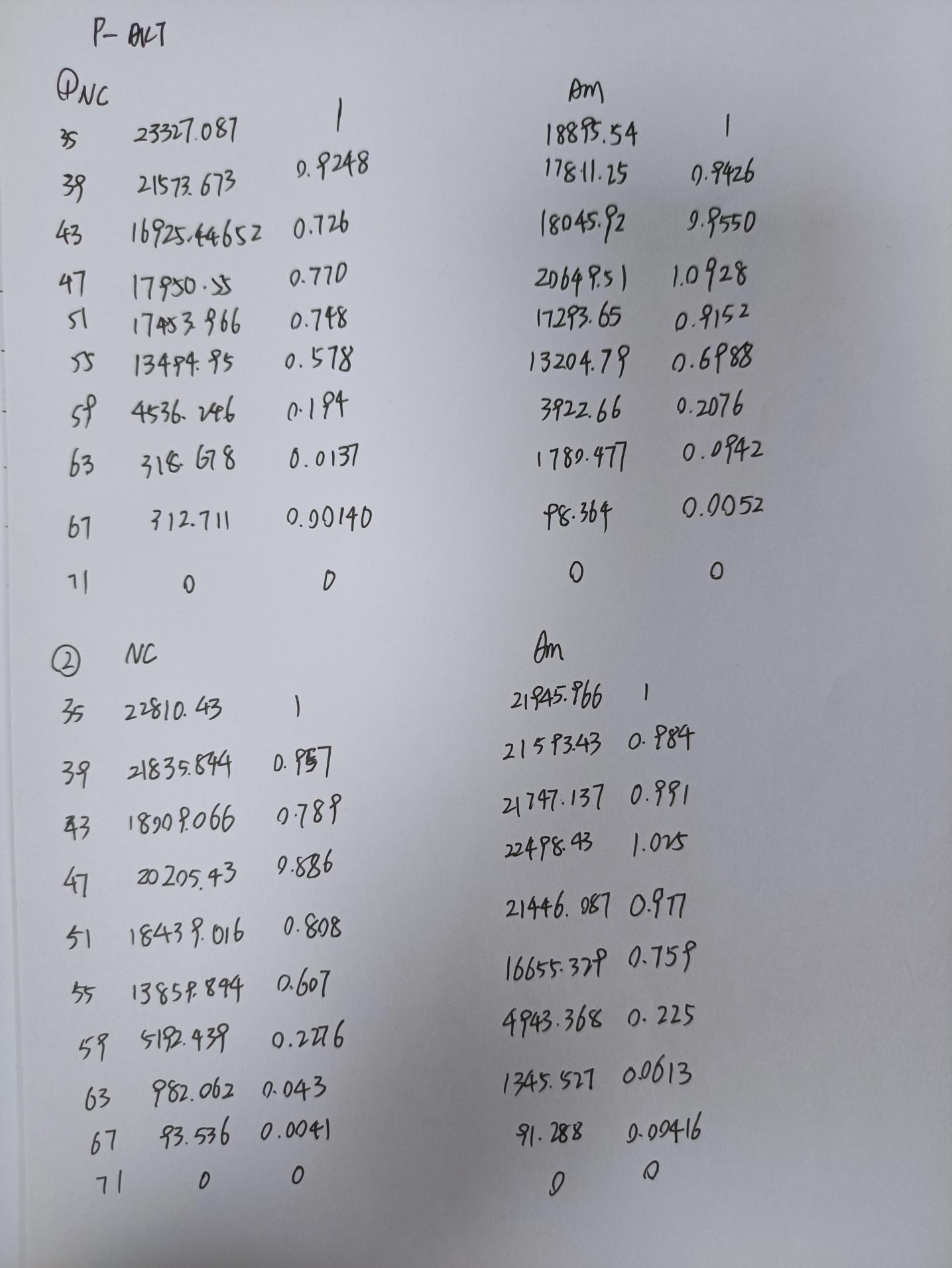

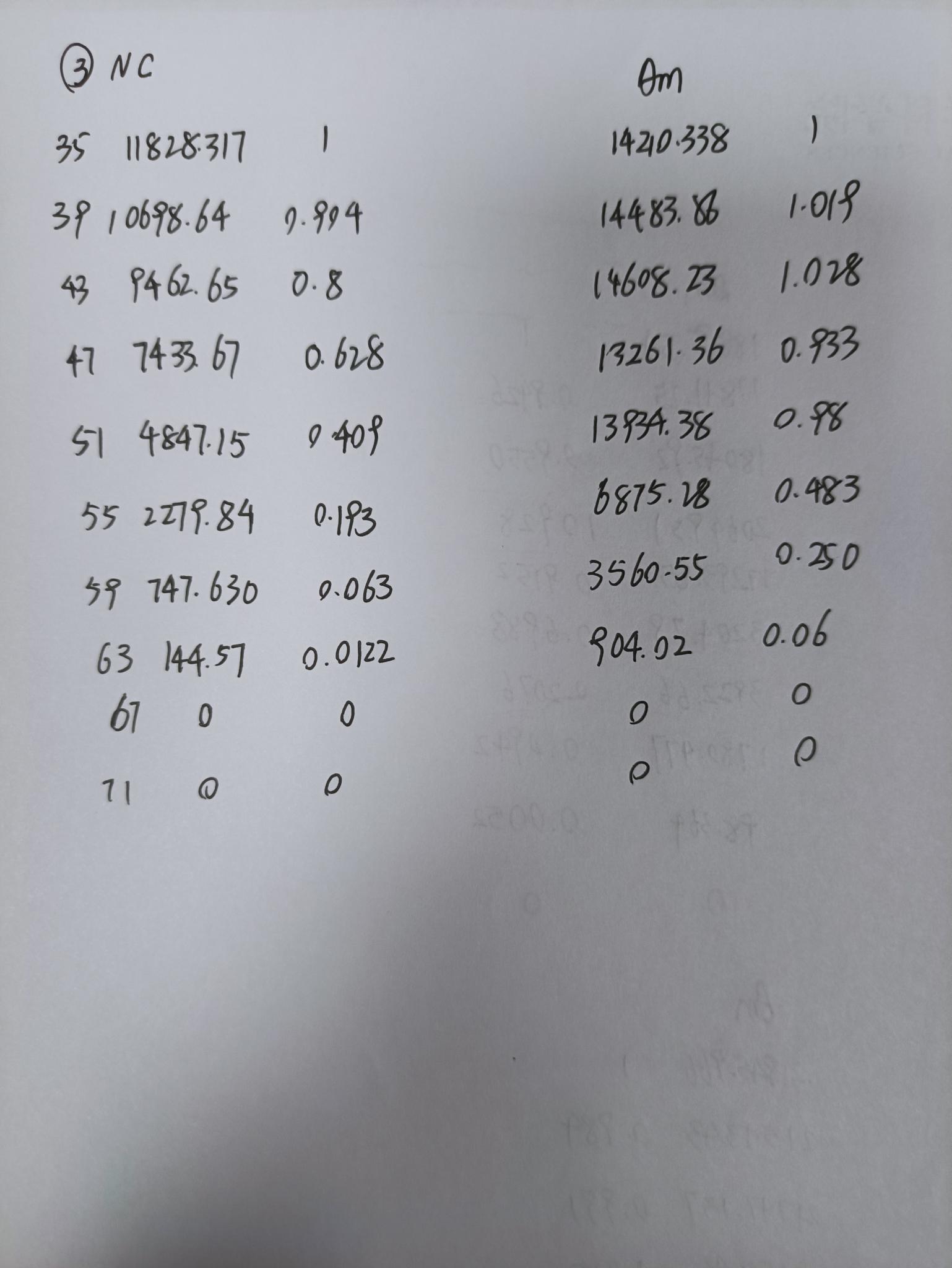
**

**mTOR**

**
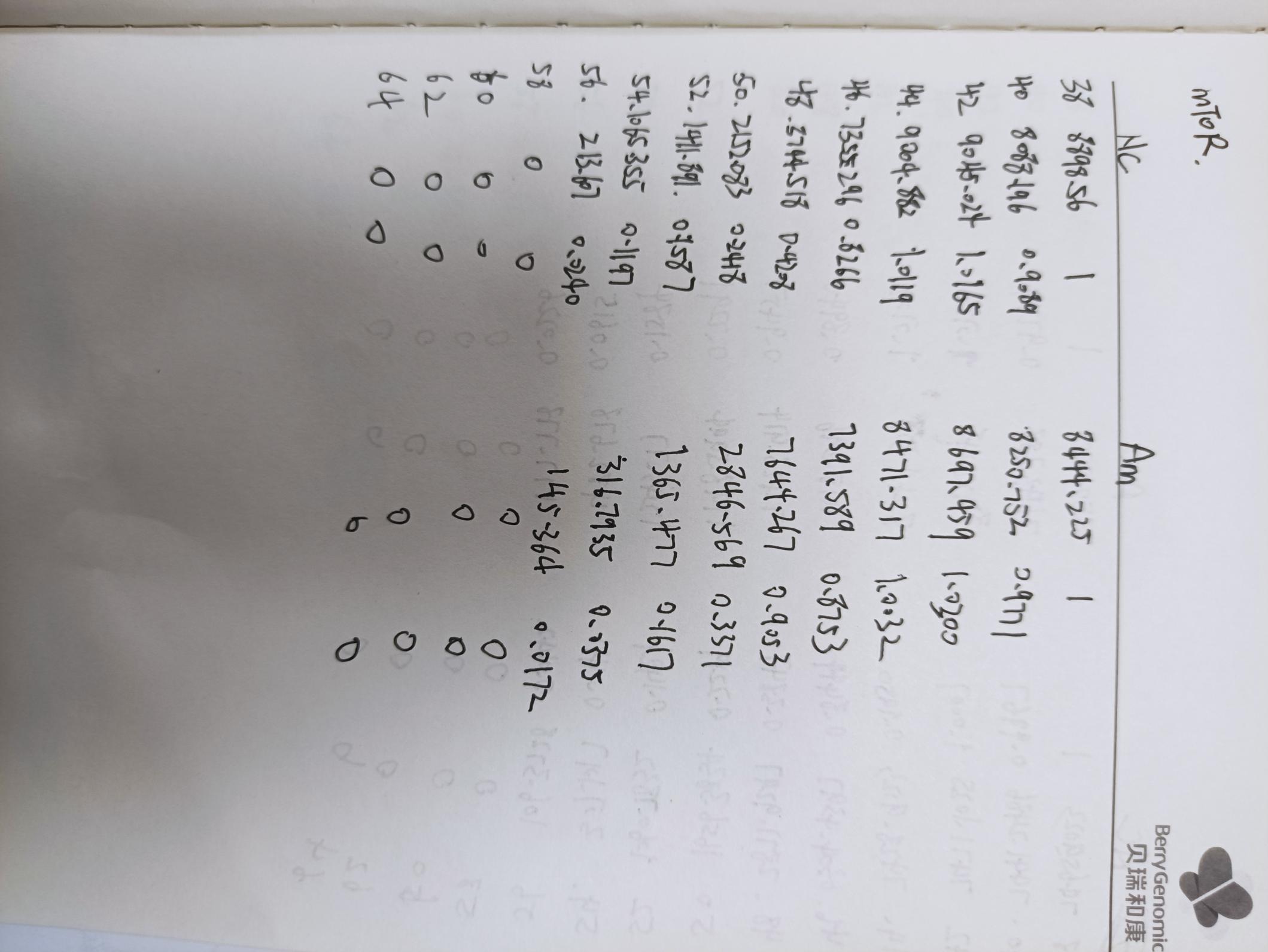

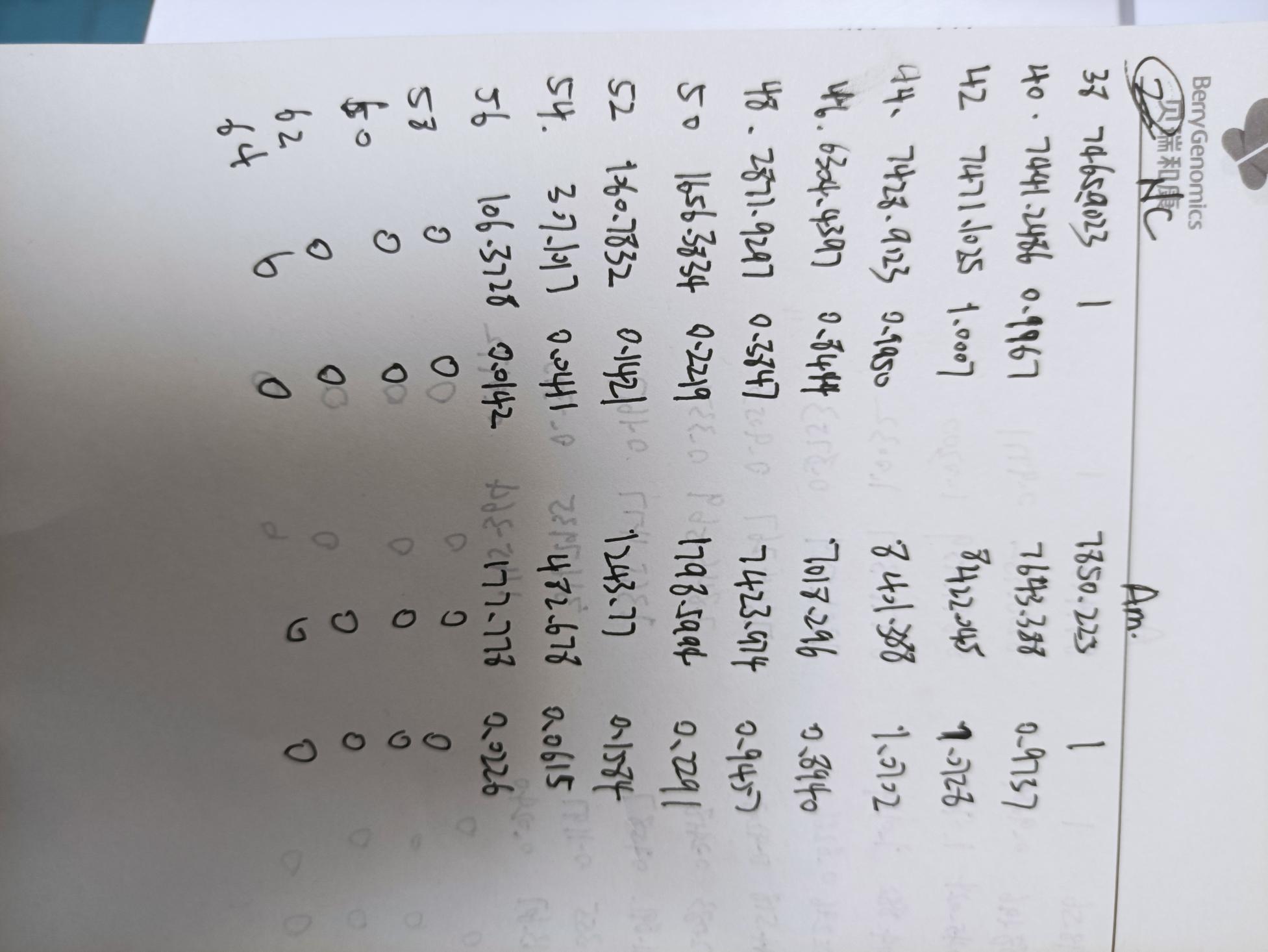

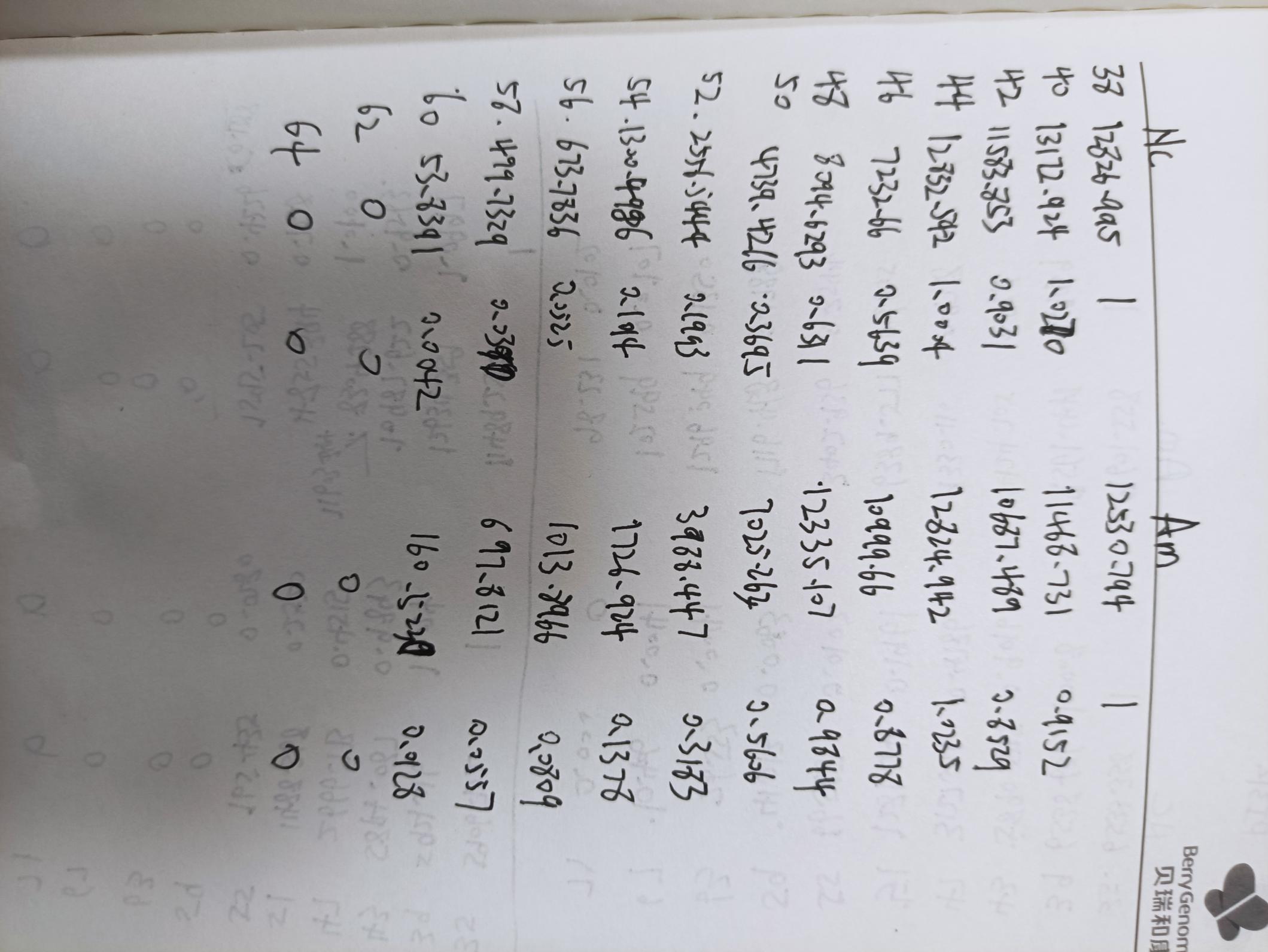
**

Supplement: Supplementary file 1 [file DataSheet1.ZIP › Original data of Aminoquinol (2)/Original data of Aminoquinol/Western Blot/TPP.docx]
