# Supplementary material for: Discovery of a New CDK4/6 and PI3K/AKT Multiple Kinase Inhibitor Aminoquinol for the Treatment of Hepatocellular Carcinoma
Source: Front Pharmacol. 2021 Jul 15;12:691769. doi: 10.3389/fphar.2021.691769 (PMC8320333; doi:10.3389/fphar.2021.691769)

**Supplementary figures**

**Table S1:** The ten highest-scoring compounds were selected based on in silico estimations of binding strength, appropriate molecular weight and other drug-like properties, and complementary matching of molecular shape.

**Table S2-7:** The CI value of aminoquinol (0, 1, 3, 5 uM) in combination with 5-Fu (0, 1, 3, 10, 30, 100 uM), the multi-kinase inhibitor sorfafenib (0, 1, 3, 10, 30, 100 uM), and the PI3K inhibitor LY294002 (0, 1, 3, 10, 30, 100 uM). The Combination Index (CI) data was analyzed by CompuSyn software analysis of the Combination Index (CI).

**Figure S1:** The differential expression level of (A) CDK4, (B) CDK6, (E) PI3K, (F) AKT and (G) mTOR in normal and tumor tissues as assessed in HCC (TCGA data, blue box for normal tissues, n = 50; red box for tumor tissues, n = 371). Kaplan-Meier survival curves of OS based on (C) CDK4, (D) CDK6, (H) PI3K, (I) AKT and (J) mTOR expressions in HCC patients. Data were presented as means ± standard deviation. * *p* < 0.05, ** *p* < 0.01, *** *p* < 0.001, **** *p* < 0.0001, significantly different from the control group.

**Figure S2:** Huh7 cells or (B) HepG2 cells were treated for 72 hrs with aminoquinol (0, 1, 3, 5 uM) in combination with the multi-kinase inhibitor sorfafenib (0, 1, 3, 10, 30, 100 uM), and cell viability determined. The combined effects and the specific combination index (CI) were analyzed by CompuSyn software.

**Figure S3:** Western blot analysis of Huh7 cells treated with indicated concentrations of aminoquinol for 6 hours.

Table S1


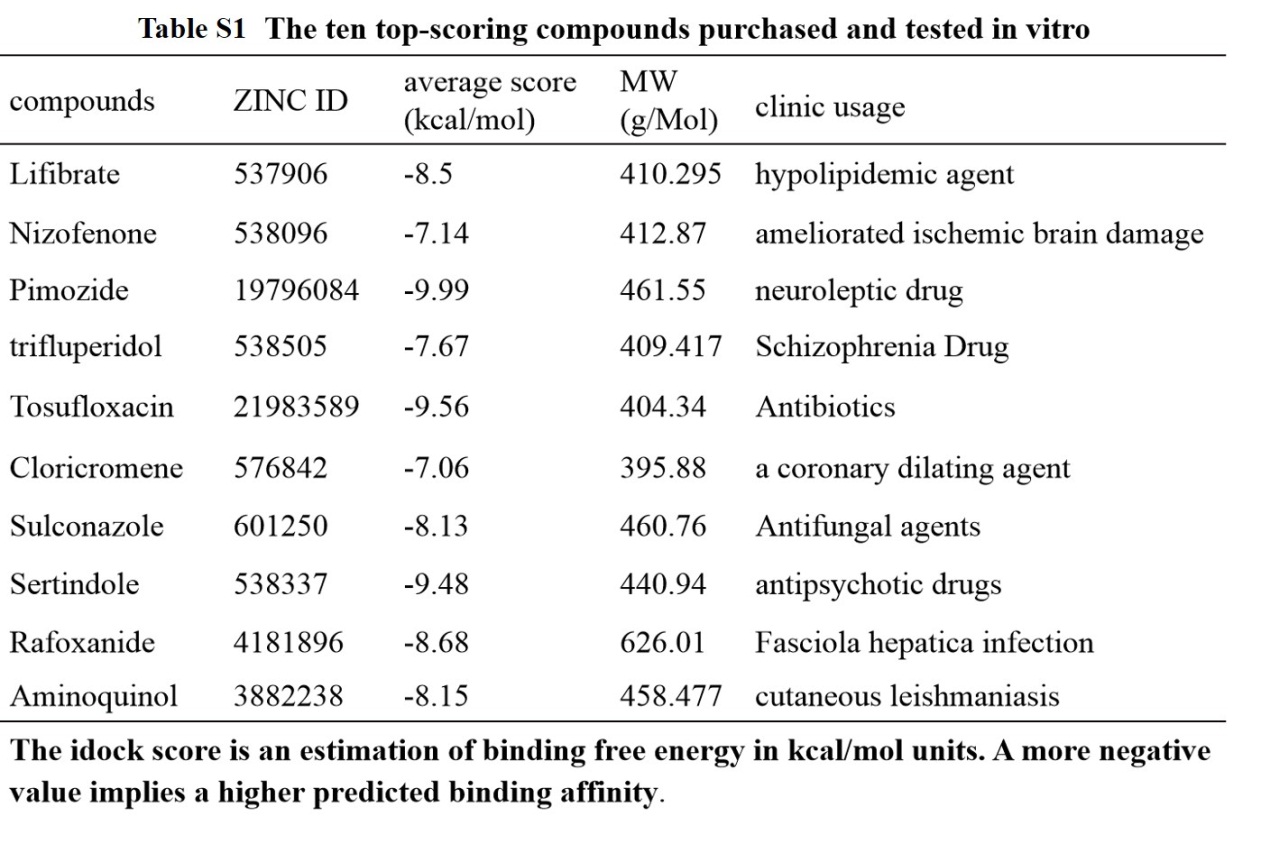


Table S2


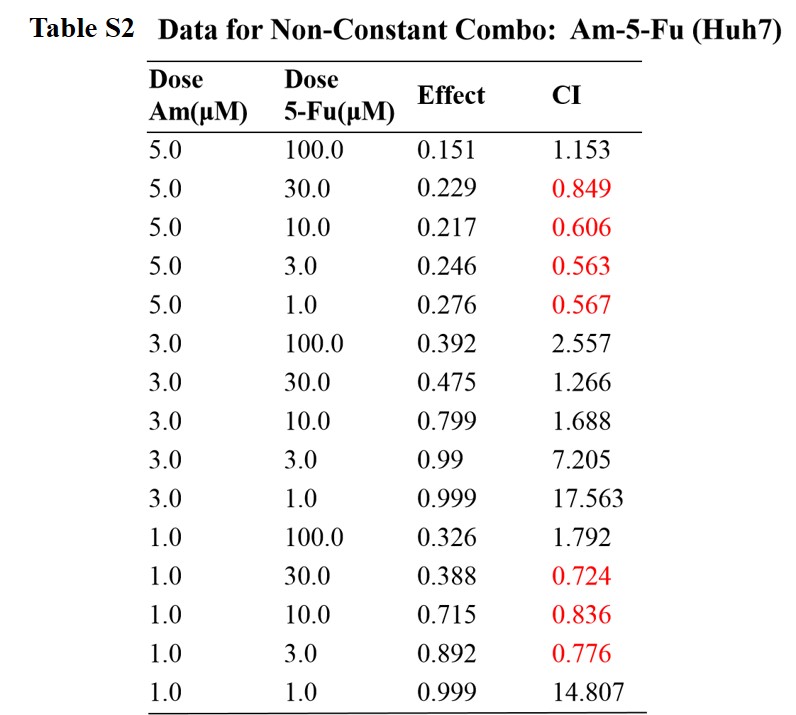


Table S3


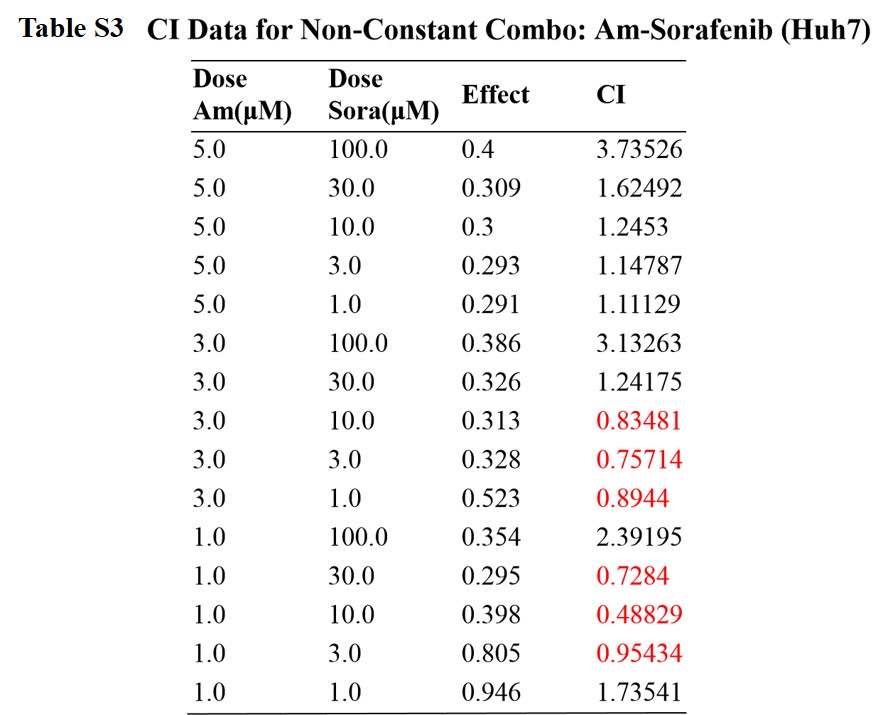


Table S4


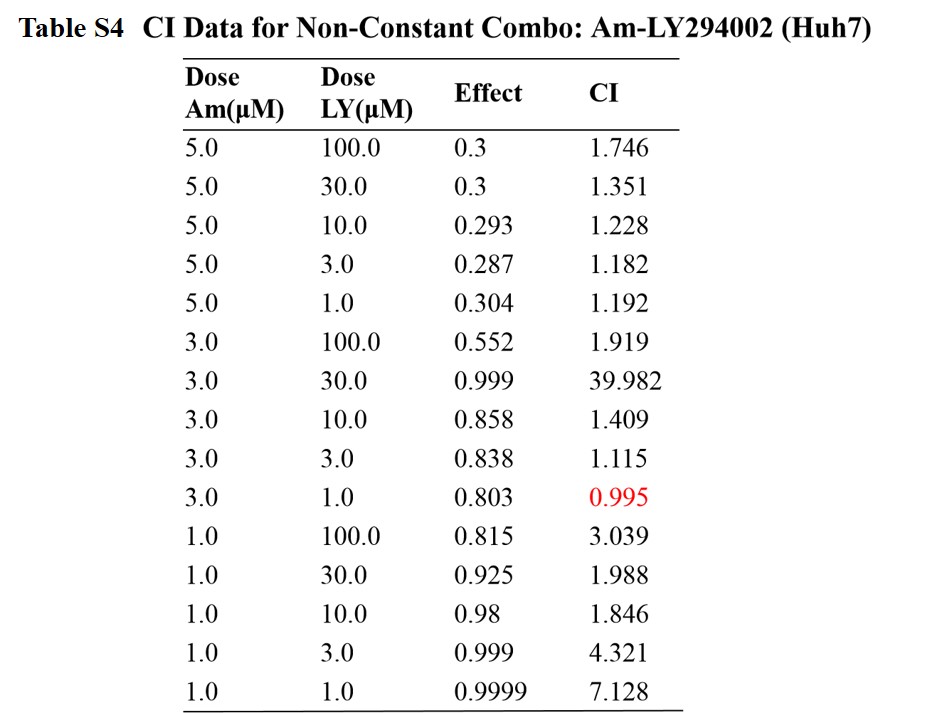


Table S5


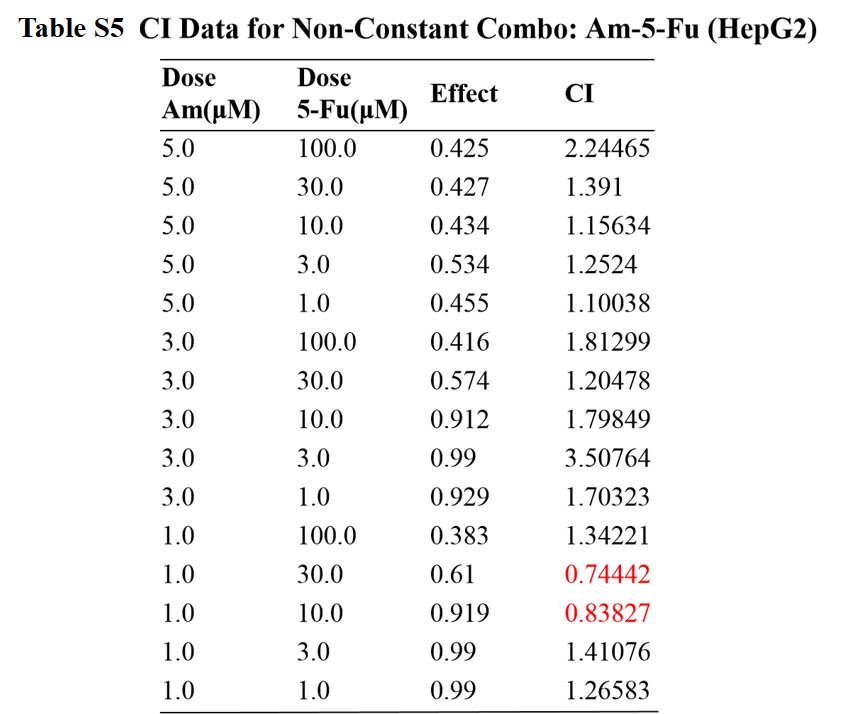


Table S6


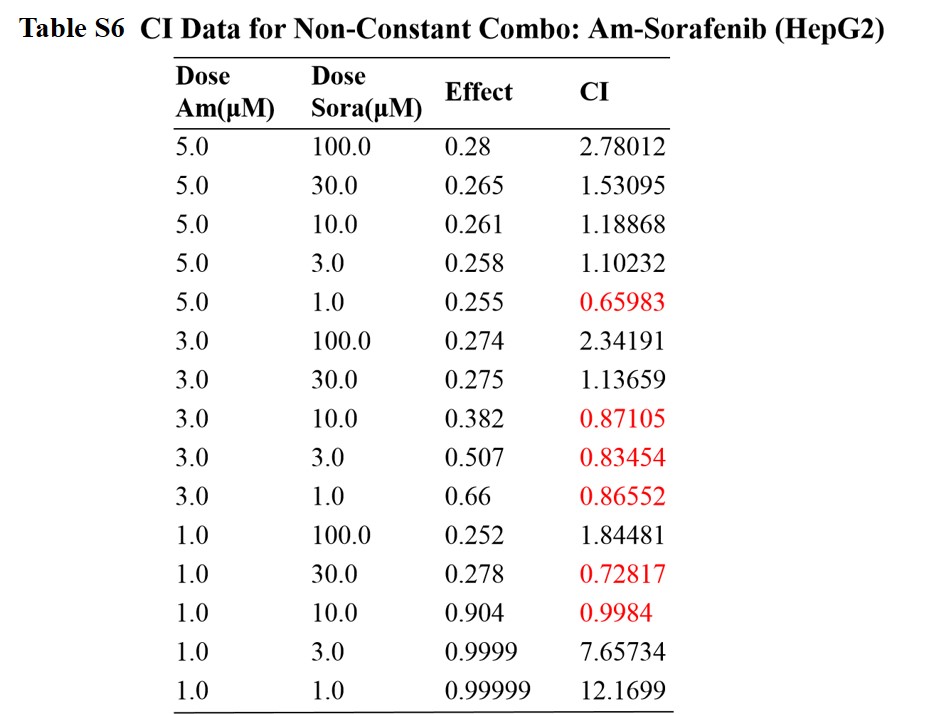


Table S7


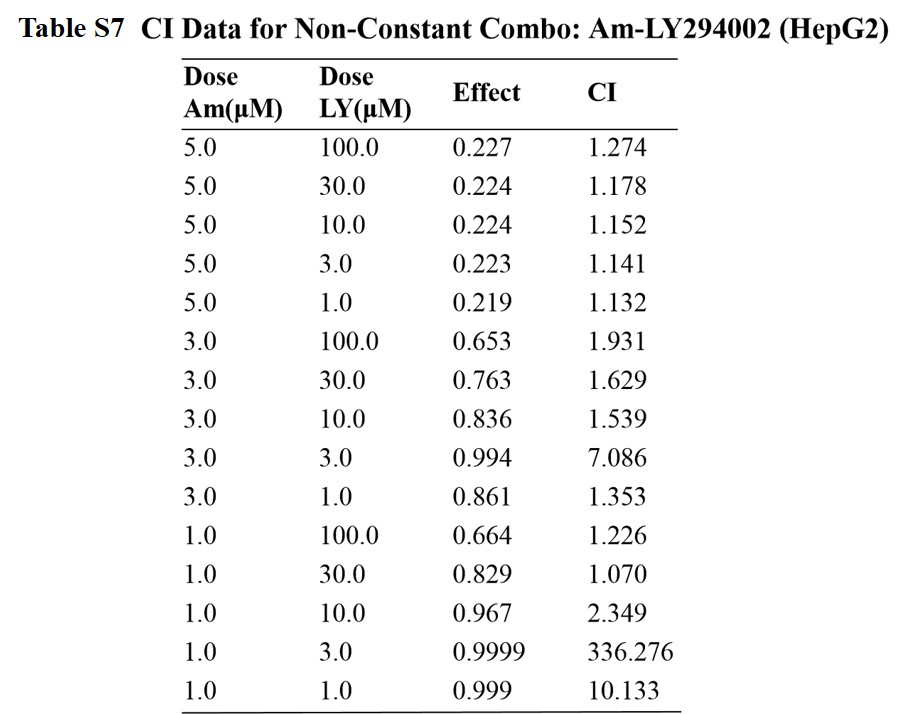


Figure S1


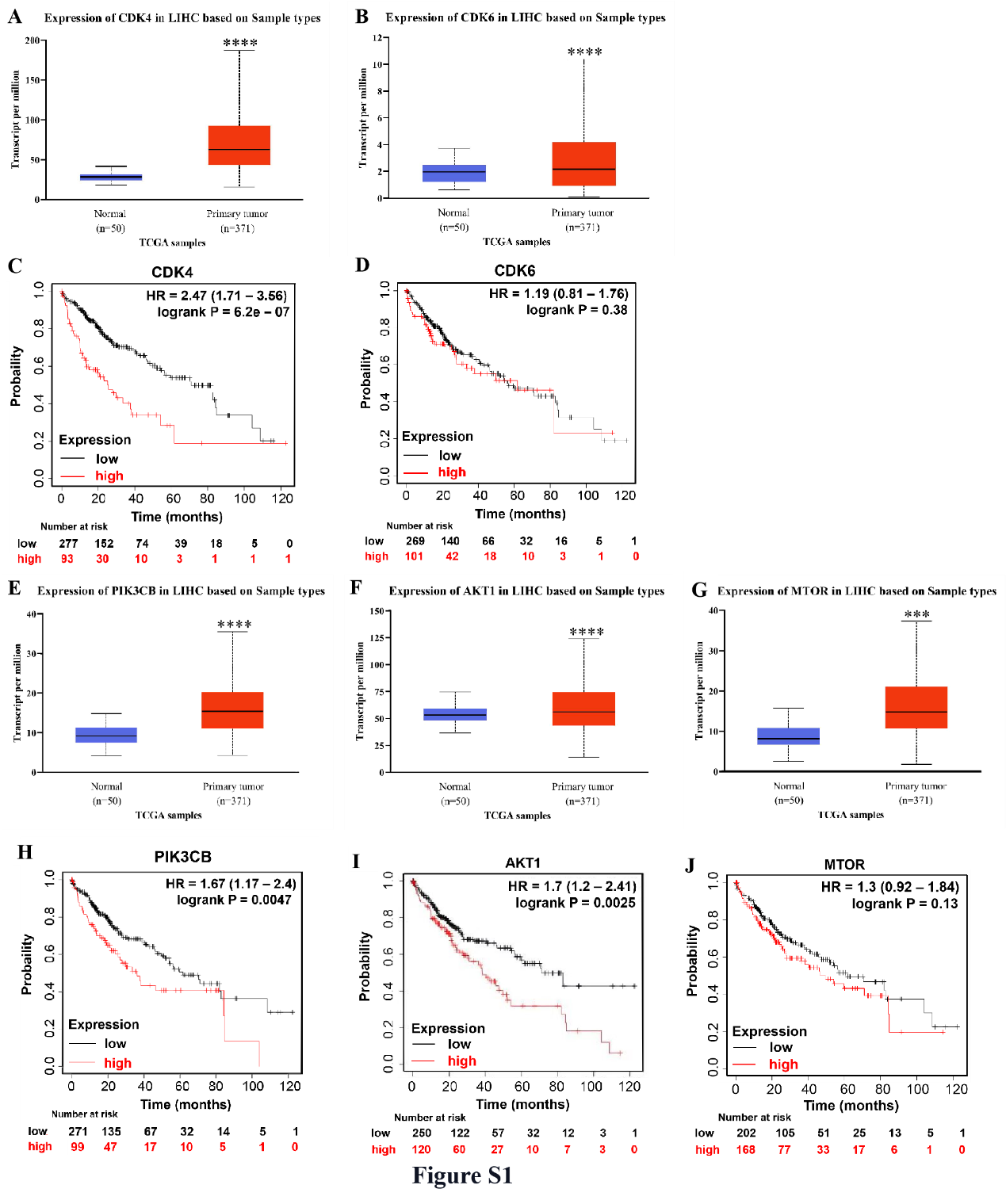


Figure S2


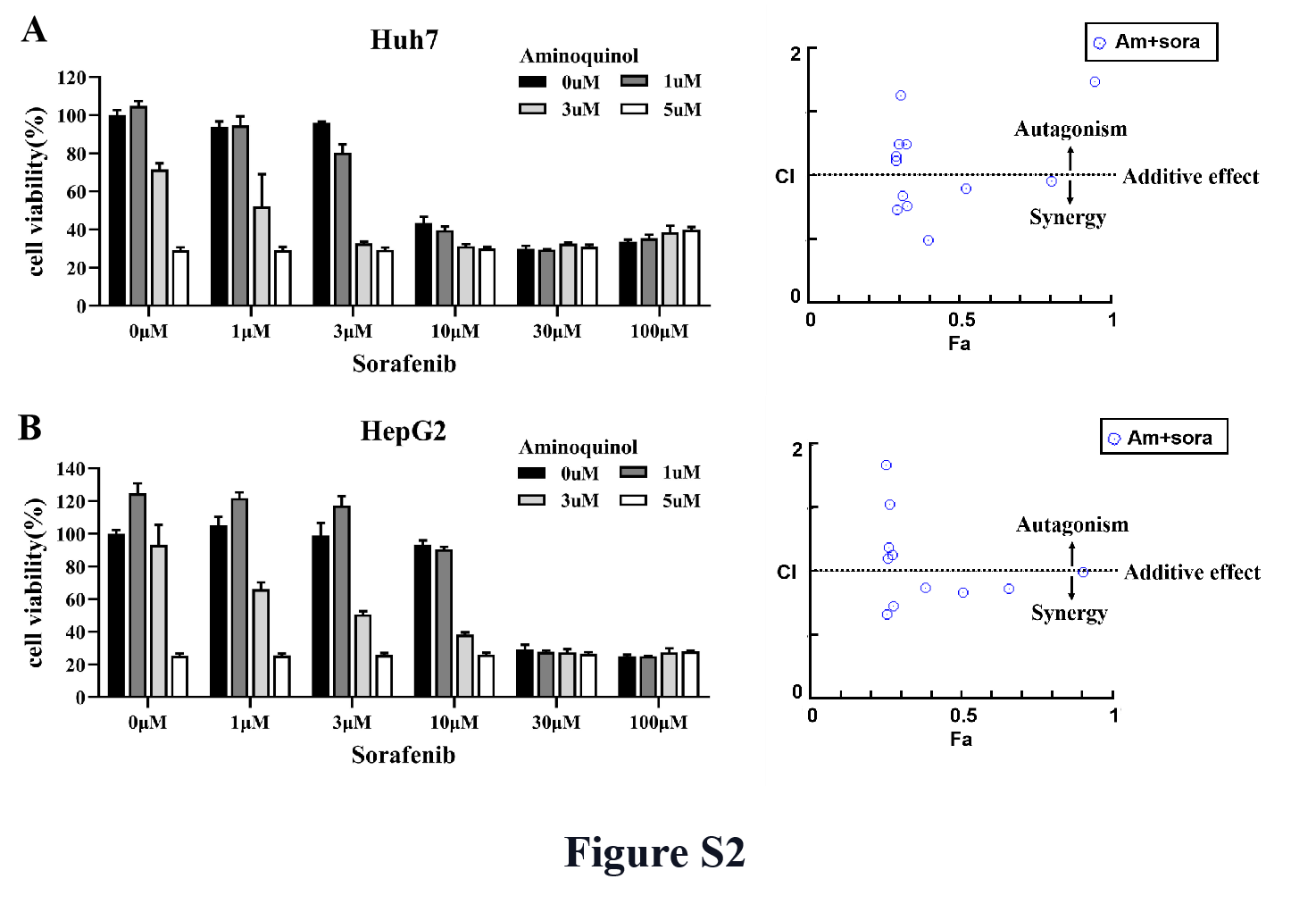


Figure S3


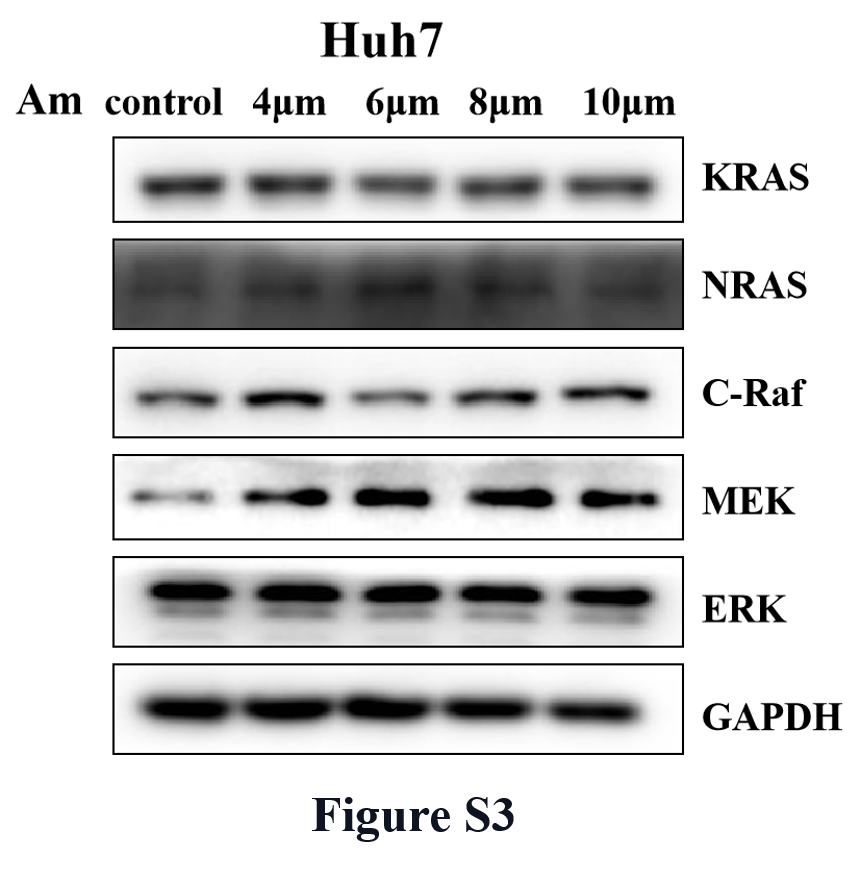

Supplement: Supplementary file 2 [file DataSheet2.docx]
